# Self-Generation in the Context of Inquiry-Based Learning

**DOI:** 10.3389/fpsyg.2018.02440

**Published:** 2018-12-13

**Authors:** Irina Kaiser, Jürgen Mayer, Dumitru Malai

**Affiliations:** ^1^Department of Biology Education, University of Kassel, Kassel, Germany; ^2^Department of Empirical School and Teaching Research, University of Kassel, Kassel, Germany

**Keywords:** generation effect, inquiry-based learning, scientific reasoning skills, control of variables strategy, prior knowledge, self-generation success

## Abstract

Self-generation of knowledge can activate deeper cognitive processing and improve long-term retention compared to the passive reception of information. It plays a distinctive role within the concept of inquiry-based learning, which is an activity-oriented, student-centered collaborative learning approach in which students become actively involved in knowledge construction by following an idealized hypothetico-deductive method. This approach allows students to not only acquire content knowledge, but also an understanding of investigative procedures/inquiry skills – in particular the *control-of-variables strategy (CVS)*. From the perspective of *cognitive load theory*, generating answers and solutions during inquiry-based learning is inefficient as it imposes an intrinsic and extraneous load on learners. Previous research on self-generation of content knowledge in inquiry-based learning has demonstrated that (1) a high cognitive load impairs retention of the generated information, (2) feedback is a fundamental requirement for self-generation of complex content knowledge, (3) self-generation success is key to long-term retention, and (4) generating and rereading place different demands on learners. However, there is still no research on the self-generation of scientific reasoning skills (procedural knowledge) and no knowledge of interaction between the (long-term) retention of these skills with prior knowledge, feedback and self-generation success. That is why this experiment was conducted. The focus of this research is to analyze the distinctive role of self-generation of scientific reasoning skills within the concept of inquiry-based learning and to identify the influence of prior knowledge and self-generation success on short-term and long-term retention. For this purpose, an experiment involving 133 6th and 7th graders was conducted. An inquiry activity that included the self-generation of scientific reasoning skills was compared to an inquiry task that had students simply read information about the experimental design. We used both an immediate and a delayed test to examine which treatment better developed a deeper understanding of CVS and an ability to apply this knowledge to novel problems (transfer). Direct instruction was clearly superior to self-generation in facilitating students’ acquisition of CVS immediately after the inquiry task. However, after a period of 1 week had elapsed, both treatment conditions turned out to be equally effective. A generation effect was only found among students with high self-generation success after a 1-week delay.

## Introduction

### Effectiveness of Inquiry-Based Learning

Inquiry-based learning is a central form of teaching and learning in science classes. It is an activity-oriented, student-centered and collaborative learning approach that has gained more and more prominence in recent years. In inquiry-based learning students become actively involved in knowledge construction by following an idealized hypothetico-deductive method (Hmelo-Silver et al., 2007). Inquiry-based learning classes can take different forms, but have two principles in common: deep active engagement and opportunities to collaborate. Experiments conducted in biology courses in order to gain new knowledge (e.g., analyzing the influence of light on the growth of plants) are a natural setting for inquiry-based learning. Taking a specific scientific phenomenon as a basis, students investigate authentic scientific problems by generating hypotheses, planning and conducting experiments and finally analyzing their data. Not only content knowledge, but also scientific reasoning skills can be acquired during scientific investigations (Klahr and Dunbar, 1988; Klahr, 2000; Mayer and Ziemek, 2006; Mayer, 2007). While content knowledge primarily plays a decisive role in two special phases, including *generating hypotheses* and *analyzing data*, scientific reasoning skills are of great importance for *planning experiments* and *discussing the results*. Inquiry-based scientific investigation is recommended in the National Science Education Standards as a unique science teaching approach focusing on scientific reasoning skills. These include the ability to use a variety of cognitive and laboratory tools of science, plan appropriate investigations, to construct arguments on the basis of evidence and communicate the results of one‘s investigations (National Research Council, 2013). A fundamental part of the inquiry process and a defining element of the scientific endeavor is an understanding of the importance and principles of unconfounded evidence (Chen and Klahr, 1999; Kuhn and Dean, 2005). This foundational skill in scientific reasoning plays a key part in science education and is referred to as *Control of Variables Strategy (CVS)* (Linn et al., 1981; Chen and Klahr, 1999). It describes the ability to design a controlled experiment by keeping extraneous variables constant while investigating a factor/factors of interest. Following this strategy severely limits one’s possible selections from the experiment space consisting of all possible experiments that could be conducted (Klahr and Dunbar, 1988). The full strategy also involves being able to distinguish between confounded and unconfounded experiments in order to assess the validity of scientific claims (Zimmerman et al., 1998). Although previous studies have found that students of all ages have trouble in understanding and applying the concept to scientific inquiries involving cause-and-effect-relationships (Sneider et al., 1984; Ross and Cousins, 1993), it has been shown that once students understood CVS, they adopted the procedure and were able to almost routinely design controlled experiments by varying one independent factor and holding untested variables constant across all conditions (Chinn and Malhotra, 2002). Nevertheless, the application of CVS is influenced by the context and content of the phenomenon encountered (Lawson, 1985). Yet research has shown that an understanding of the principles of unconfounded evidence does not automatically develop without explicit instruction or practice (Sneider et al., 1984; Schwichow et al., 2016). Furthermore, there is some controversy about the most effective approach to teaching CVS. Vollmeyer and Burns claim that students are able to figure out more about the functioning of a system through undirected exploration, thus resulting in richer learning outcomes (Vollmeyer and Burns, 1996), while Klahr and Nigma found evidence that direct instruction is more effective than discovery methods when it comes to teaching CVS (Klahr and Nigam, 2004). While meta-analyses of inquiry-based learning in science have found (relatively modest) positive gains from using inquiry-based learning (Furtak et al., 2012), the theories underlying the effectiveness of inquiry-based learning are still quite controversial (Kirschner et al., 2006; Hmelo-Silver et al., 2007).

### Self-Generation in the Context of Inquiry-Based Learning

Self-generation plays an important role in the concept of inquiry-based learning. However, the unique effect of this factor within the broader concept of inquiry-based learning is still unclear. Supporters of maximum open-endedness (*open inquiry*) have long considered a high degree of open-endedness – involving high self-generation requirements and the withholding of information (e.g., Zion and Mendelovici, 2012) – the optimal strategy for promoting effective learning. Recent findings in cognitive psychology support this approach, arguing that long-term learning effects and knowledge transfer are only possible with high self-generation requirements (Chi, 2009; Bjork and Bjork, 2011). These findings are based on a simple and obvious strategy of human learning: In contrast to passive reading, the active self-generation of knowledge, or the active involvement in knowledge construction, enhances long-term retention. As a result, actively generated information is retrieved more successfully than passively learned information. This phenomenon is referred to as *generation effect* (Slamecka and Graf, 1978; Bertsch et al., 2007). It is a robust, generally valid cognitive psychological finding that consistently occurs with a remarkable effect size (Bertsch et al., 2007). While the mechanisms underlying the effect are still not fully understood, the most widely accepted account is that self-generation enhances cognitive effort, item distinctiveness, and semantic processing (e.g., Hunt and McDaniel, 1993; Steffens and Erdfelder, 1998). The positive effects persist across different kinds of test paradigms for measuring memory (including recognition, cued recall, and free recall), and for various study paradigms, including intentional and incidental learning. Moreover, many studies have demonstrated a generation effect not only for content knowledge, but also for strategies and procedures such as multiplying or adding numbers (McNamara, 1995). In fact, the effects for procedural knowledge are much larger than for linguistic information (Bertsch et al., 2007). This is relevant for inquiry-based learning because its instructional model foresees learning not just with respect to scientific concepts (declarative knowledge), but also scientific reasoning skills (declarative and procedural knowledge) (Klahr and Dunbar, 1988; Klahr, 2000).

Despite the advantages of self-generation, the active construction of knowledge in an authentic learning environment is always coupled with a high investment of cognitive effort – referred to as *cognitive load* (Clark and Linn, 2003).

### Cognitive Load

Processing a large number of elements in working memory at the same time while generating new information and developing a sense of coherence among them may lead to cognitive overload if no guidance is provided (e.g., Sweller and Chandler, 1994; Chen et al., 2016). This might inhibit long-term retention (e.g., Klahr and Nigam, 2004; Kirschner et al., 2006). There are three types of cognitive load: intrinsic, a measure of complexity of the learning material itself; extraneous, the manner in which the tasks are presented; and germane, which serves to facilitate students’ understanding and automation of the information received during instruction into long-term memory (Paas et al., 1994; van Merriënboer and Sweller, 2005).

Authentic learning settings, particularly in science education, entail a high element interactivity and thus high intrinsic cognitive load. As a result, opponents of open inquiry question the long-term effectiveness of an open-ended form of learning (e.g., Klahr and Nigam, 2004; Kirschner et al., 2006). They demonstrate that high self-generation requirements increase learners’ intrinsic and extraneous cognitive load, as greater open-endedness goes hand in hand with a lack of specific instructions. They argue that more guidance should be provided in order to reduce a mental exertion (Sweller et al., 2011). Only the presence of guidance and assistance (*guided/structured inquiry, confirmation inquiry*) can reduce learners’ cognitive load and engender strong long-term learning effects (Kirschner et al., 2006). The higher the complexity of the learning content or the level of element interactivity (intrinsic cognitive load), the more guidance is required (Chen et al., 2016). However, studies on the best balance of giving and withholding guidance and assistance in inquiry learning in order to achieve optimal learning outcomes have arrived at different results (*assistance dilemma*, Koedinger and Aleven, 2007): while providing greater performance assistance (e.g., feedback) during instruction can sometimes improve learning, in other cases making the acquisition of knowledge more difficult during instruction (e.g., by increasing self-generation requirements and reducing feedback) enhances learning outcomes and the transfer of knowledge (Schmidt and Bjork, 1992). Frequent feedback might block the processing of response-produced feedback, meaning that students are not given the chance to learn to identify their own errors and benefit from them (Schmidt et al., 1989). However, recent findings in natural learning environments have confirmed the importance of a mid-level of assistance (e.g., Kirschner et al., 2006; Koedinger and Aleven, 2007; Borek et al., 2009; Chen et al., 2016; Kaiser and Mayer, unpublished). For example, it was shown that students in a pure self-generation condition without any feedback (minimal assistance) recorded higher cognitive load scores than students who continuously received corrective feedback (mid-level assistance) or direct instructions (high assistance). Furthermore, retention as well as self-generation success during the learning process both increased as a result of feedback (Kaiser and Mayer, unpublished). But at the same time, providing direct instructions (e.g., in the form of reading texts) proved to be equally effective as a mid-level of assistance (corrective feedback) (Kirschner et al., 2006; Kaiser and Mayer, unpublished). Thus, feedback is a decisive requirement for the self-generation of complex knowledge. Its important influence on learning outcomes and memory is due to the provision of information that may not have been successfully generated and the resulting decrease in cognitive load. Further experimental studies are needed to detect specific qualitative conditions and quantitative thresholds that can support instructors in selecting the optimal amount of assistance (Koedinger and Aleven, 2007). Moreover, this process is regulated by learner characteristics.

### The Role of Learner Characteristics (Prior Knowledge, Cognitive Abilities, Need for Cognition)

Multiple learner characteristics, such as need for cognition, cognitive abilities, and prior knowledge, have an influence on self-generation in inquiry-based learning. Need for cognition (NFC) is a learner’s tendency to engage in and enjoy effortful cognitive endeavors (Cacioppo and Petty, 1982) and has an influence on whether or not a deeper learning strategy like self-generation is applied. A number of studies have demonstrated that NFC plays a distinctive role in active information processing as a descriptor and predictor, over and above cognitive abilities (e.g., Cacioppo et al., 1996; Cohonner and Mayer, 2018). Recent findings revealed that more than 50% of the variance in performance (success in self-generation) during the inquiry task could be explained by NFC and another important factor – cognitive abilities [measured by school type^1^ (Kaiser and Mayer, unpublished)]. The latter represent one determinant of an individual’s learning capacity in the sense of the *Berlin Model of Intelligence* (Jäger, 1984). Pattern recognition and inductive thinking constitute a necessary basis for all scientific inquiry^2^ (Kuhn et al., 1988). They are special abilities referring not only to detecting patterns, resemblances, or other kinds of regularities, but also applying simple logic in order to predict what will happen next.

Of equal importance are prior knowledge and experience, which can additionally reduce intrinsic cognitive load during the process of self-generation (e.g., experimental investigations) above and beyond feedback. Consequently, students with low prior knowledge are less likely to benefit from self-generation. Firstly, low-knowledge students have fewer opportunities to generate correct information and procedures. This leads to poorer performance in comparison to high-knowledge students (e.g., Siegler, 1991; Shrager and Siegler, 1998). Secondly, the self-generation effect is generally much weaker for the retrieval of unfamiliar material (like non-words) than for familiar material (Lutz et al., 2003). This indicates that the effect of self-generation on performance and retention should be weaker among students who are less familiar with the material. However, there are only a few studies examining the influence of prior knowledge on the generation effect. Most studies deal with non-curriculum-based material that doesn’t require any prior knowledge. Moreover, those studies that do analyze the effects of prior knowledge are often limited to mathematical contexts. Rittle-Johnson and Kmicikewycz‘s study on multiplication problems, for example, demonstrates that students with lower prior knowledge benefited from self-generating answers to problems. They solved more problems correctly across the posttest and retention test than comparable students in the read-from-calculator condition, even on unpracticed problems (Rittle-Johnson and Kmicikewycz, 2008). Hence, while Chen and colleagues showed that explicit instruction is essential for low-knowledge students when it comes to complex material (Chen et al., 2016), these cognitive psychology findings reveal that students’ prior knowledge and intuitions often conflict with new knowledge (Bransford et al., 1999).

### The Role of Self-Generation Success

The effectiveness of inquiry-based learning has generally only been demonstrated for learning outcomes examined after the inquiry session via domain knowledge posttests. In contrast, embedded assessments of the products students create during inquiry (i.e., performance success) have rarely been conducted, and the influence of this data has rarely been analyzed (Lazonder and Harmsen, 2016). Nor has successful self-generation played a significant role in most studies on the generation effect, as they dealt with simple material that could be successfully generated by most learners without any prior knowledge in the encountered domain. Only a very small number of studies have examined the generation effect with respect to complex and scientific content, taking self-generation success into account (e.g., Foos et al., 1994; Richland et al., 2007). Surprisingly, they came to contradictory findings: Foos et al. (1994) pointed out that the effect has generally not been found in conventional settings because the analysis of total test performance rather than just (successfully) generated items masked the effect. He found that the generation effect occurred only for (successfully) generated items but not for non-generated items (Foos et al., 1994). In contrast, Richland and colleagues suggested that performance during learning is “an unreliable predictor of long-term learning and transfer” (Richland et al., 2007, p. 1850). Their investigations of self-generation using educationally relevant science material and an educational software platform (WISE) revealed that students in the generation condition exhibited a higher error rate during learning than those in the read condition, whereas after a delay of 2 days, retention of single fact materials was higher in the generation condition than in the control group. However, they also found a generation effect only for facts that were generated successfully (Richland et al., 2007).

## Research Questions

The goals of the present study are to investigate the effect of the active self-generation of scientific reasoning skills, determine the extent to which students transfer the CVS and generalize it across various contexts, and identify the role of students’ prior knowledge, cognitive abilities, cognitive load and self-generation success in the long-term retention of information generated during inquiry.

For this purpose, an experiment involving 6th and 7th graders was conducted. An inquiry activity that included the self-generation of scientific reasoning skills was compared to an inquiry task that had students simply read information about the experimental design.

The following questions were analyzed:

(Q1) Does self-generation of scientific reasoning skills during inquiry have an effect on long-term retention among 6th and 7th graders (in comparison to reading)?

Although direct instruction is more effective than discovery learning in teaching students CVS (Chen and Klahr, 1999; Klahr et al., 2001; Klahr and Nigam, 2004), skills obtained through self-generation in the discovery learning condition should be more advantageous in the long run, as self-generating the answer rather than simply reading it gives rise to a well-documented beneficial memory effect (Slamecka and Graf, 1978). (H1)

(Q2) How do intrinsic, extraneous and germane cognitive load measured immediately after the learning process differ between the two conditions (self-generation and reading)?

Like all other types of desirable difficulties, self-generation briefly increases learners’ intrinsic and extraneous cognitive load and impedes their learning process, as germane cognitive load is decreased. (H2)

(Q3) Do success in self-generation and perceived cognitive load have an influence on the effect of self-generation?

Generating materials should specifically make a difference for items successfully generated during the learning process (Foos et al., 1994). It has proven to be a reliable predictor of learning outcomes (Kaiser and Mayer, unpublished). (H3)

(Q4) Do individual prerequisites (e.g., prior knowledge, need for cognition, cognitive abilities) affect self-generation success and the learning outcomes in both conditions?

High cognitive abilities and a high need for cognition moderate learners’ self-generation success (Kaiser and Mayer, unpublished). Both performance and retention should be higher among students who have high prior knowledge on the principles and procedures of CVS. (H4)

## Methods

### Participants

An *a priori* power analysis using G^∗^Power (Software G^∗^Power; Faul et al., 2007) with a significance level of *a* = 0.05, a (conservatively estimated) medium effect size of *f* = 0.25, and a desired power of (1 – b) = 0.95 revealed a suggested sample size of *N* = 72. Based on this analysis, 168 German 6th and 7th graders from 7 classes and 5 different schools in Kassel, aged between 11 and 14 years, participated in our study. The five 6th grade classes were part of the “promotion level”^3^, while the two 7th grade classes were within highest-track schools (Gymnasium). Student absenteeism reduced the original sample to 153 students who were present during the introductory session. Out of these, 133 students with an average age of 12.07 years (*SD* = 0.667) completed the task (generation with feedback: 69; control: 64) and the first and second posttest, and 109 students also completed the follow-up 4–6 weeks later (generation with feedback: 58; control: 51). The latter two sample reductions were due to student illness or failure to consent to the use of their data. All data was collected and analyzed anonymously.

### Research Design

The study followed a 2 (learning condition) × 3 (retention interval) mixed factorial design. Two types of encoding formats– self-generation with feedback (GF) vs. reading (R) – served as the independent variables. The dependent variables in the assessment phase comprised the posttest performance immediately after the intervention as well as 1 and 4–6 weeks later. Thus, we contrasted the learning and transfer effects of learning the control-of-variables strategy (CVS) via either self-generation or reading in both the short- and the long-term. In order to control for unexpected text effects the items we used for each test varied.

### Learning Content and Materials

In this unit, students acquired the basic scientific reasoning skills of hypothesizing, experimenting, and evaluating evidence and an understanding of variable control procedures and strategies (CVS).

Since only a few students had prior knowledge of and experiences with control of variables through inquiry activities, they all participated in a uniform computer-based introductory session designed to increase students’ ability to control variables. An illustrative experiment about dragonfly (*Anisoptera*) larva hunting their prey (depending on the prey’s size) simultaneously familiarized them with variable control strategies as well as the concept of behavioral adaptation, which was the learning content for this unit. Thus, the example of dragonfly larva hunting their prey was used to introduce the students to the four-phase method of scientific inquiry: (1) formulate research questions, (2) generate one or more hypotheses, (3) plan and conduct an experiment, and (4) analyze the experiment (describing the data, interpretation, critically evaluating the methods used).

In the laboratory sessions, the students conducted a scientific experiment to investigate a related phenomenon also involving the concept of biological adaptation (structural or behavioral changes that help an organism survive in its environment), which is a disciplinary core idea in the Science Standards (National Research Council, 2013). It was called “The Mystery of Water Fleas’ Migration” (Meier and Wulff, 2014) and dealt with the periodic daily vertical migration of water fleas (*Daphnia magna*), which none of the participating classes had covered previously in class. The students received a research workbook developed by the authors to support their learning process and guide them through the process of a scientific study (hypothesis generation – planning and conducting an experiment – drawing conclusions).

Students in the self-generation condition documented their answers in this workbook. The format in the self-generation condition consisted of 13 short self-generation prompts relating to the independent and dependent variables, control variables, and confounded variables (short answer tasks), e.g., *Example 1*, as well as a cloze (with 130 words and 15 prompts) – retrieving information about the CVS – at the end of the experimental session, e.g., *Example 2* (for all prompts see Supplementary Material: research workbook). In comparison, the research workbooks for the reading condition consisted of direct experimental instructions rather than generation prompts and a reading text instead of a cloze. To ensure that the information was structured in similar way in all encoding formats and to give students the same amount of time for cognitive processing, all prompts and feedback material in the self-generation treatment were derived from the text material in the reading condition.

### Procedure

The experiment included four different phases: a computer-based introduction with subsequent pretest, an inquiry-based learning session with subsequent posttest, a second posttest and a follow-up. In the first session, the students received guided instruction in a computer-based learning environment, and subsequently completed a brief learning session that familiarized them with basic scientific reasoning skills. Information was provided via videos and short reading passages. The instruction lasted 30 min and took place at school. Immediately after this session, the students were given a paper-based prior knowledge assessment test to determine individual differences in their scientific reasoning skills. The test took them about 25 min to complete; no time limit was imposed. In addition, data on the students’ demographics, cognitive abilities and need for cognition, as well as grades in Maths, German and Biology were collected.

One week later, the second phase – the scientific experiment on the adaptation of water fleas – was completed. This module took place in an inquiry-based learning environment in a student lab (the Experimental Biology Lab FLOX at the University of Kassel). The module’s focus was on imparting scientific thinking and scientific reasoning skills through guided experimentation.

At the beginning of the second module, the students within each class were randomly assigned to one of two conditions (self-generation vs. reading the experimental design and methodological discussion) and divided up into small groups (up to 5 students) instructed by specially trained supervisors. Thus, the students already knew one another. Due to organizational reasons, the availability of only one student lab, and a limited amount of experimental materials, rooms and supervisors, it was not feasible to intermix students across classes. The supervisors received detailed scripts with precise information on each inquiry phase in order to ensure that they provided uniform guidance and mentoring to all groups throughout the experiment. In order to get an authentic picture of students’ inquiry skills, supervisors in both conditions were instructed not to answer any questions asking them to explain the scientific reasoning.

Students in both conditions worked on the assignment for approximately 180 min, receiving (general) instructions from their supervisor in two separate rooms. The two encoding formats differed in the amount of information and instructional support provided; however, the total instructional time was equivalent in both conditions. An explicit time for completion was assigned to each task (see Supplementary Material: research workbook). The conditions differed with regard to the procedure during the practice phase as follows: The self-generation group was instructed to actively generate their own experimental plan and appropriately discuss their data using inquiry skills they had acquired in the introductory section, whereas students in the reading condition received a detailed experimental plan and a corresponding discussion of the method they would be using.

The varied use of different classroom formats – individual and group work – made it possible to replicate the self-generation and reading process in a natural setting. This strategy made it possible to let the students in the self-generation condition identify the independent and dependent variables and determine the control variables more than once. First, they individually generated the information by identifying the independent and dependent variables and bringing out first ideas for experimental procedures (scientific reasoning skills: generating hypotheses, aspects: independent variable, dependent variable; Arnold et al., 2014). Then, after they had discussed their tentative ideas in their groups, they collaboratively developed a detailed experimental plan by operationalizing the dependent variable, varying the independent variable in an appropriate way, identifying and controlling various biases and confounds, determining the measurement intervals and number of measurements (scientific reasoning skills: planning experiments, aspects: independent variable, dependent variable, confounded/ nuisance variables, measurement points, repeated measures; Arnold et al., 2014). They received corrective feedback after both phases (after developing their experimental plan, as well as after discussing their data). In contrast, students in the reading condition were told which variables to investigate and followed a short series of prescribed steps similar to a recipe.

The students received corrective feedback from their supervisor in order to ensure that the learning environment remained authentic and to provide them with adequate information, as students perform better during inquiry (i.e., achieve higher performance success) when supported by more specific guidance (Borek et al., 2009; Lazonder and Harmsen, 2016). Nevertheless, the information that could be provided was strictly defined in a workbook of instructions (see Supplementary Material: workbook of instructions for self-generation group), e.g., *Example 3, Example 4*, that all supervisors had to work from. By giving the students the correct responses or instruction on supplementing and/or revising proposed ideas for an experimental plan, the students were able to reject erroneous ideas and use cues to direct their search for others.

Despite the differences, however, the process sequence was identical in both conditions. Thus, all students received hypotheses and appropriate interpretations of their experimental data. They were not instructed to generate any content-related information. Moreover, students in both groups engaged in hands-on activities necessary for carrying out the experiment, as an understanding of the principles of unconfounded evidence cannot develop without explicit instruction or practice (Sneider et al., 1984; Schwichow et al., 2016).

Immediately after the inquiry-based learning session, a questionnaire asking about cognitive load and a posttest measuring scientific reasoning skills were completed by all students in both treatment groups. Both conditions received the same test. The students were not informed in advance that they were going to take the tests to prevent them from preparing for them, thus increasing the likelihood that posttest scores represented the knowledge gained during the experiment. It took them about 30 min to finish both tests; again, no time limits were imposed. One week and 4–6 weeks later, all students answered items on a second, comparable posttest and a follow-up comprising 4–6 anchor items from all three questionnaires, respectively.

### Instruments

The dependent variable, scientific reasoning skills, was tested at three different points, contrasting the learning outcomes of the treatment group (GF – Generation + Feedback) to the control group (R – Reading). Student prerequisites like prior knowledge of scientific reasoning skills, need for cognition (Preckel, 2014) and cognitive abilities (Heller and Perleth, 2000) were also assessed. In addition, the students’ success in self-generation as well as their perceived cognitive load (Cierniak et al., 2009) were measured during and immediately after the experimental unit. All measurements were paper-based.

#### Learning Outcomes

To evaluate the learning outcomes, three questionnaires were developed to assess the acquisition and retention of scientific reasoning skills. After statistical item analyses, the assessment tests consisted of 6 multiple choice items (Janoschek, 2009; Walpuski et al., 2010; Hof, 2011; modified) at each measuring point. All multiple choice items had four possible answer options, of which only one was correct (see Supplementary Material: Posttest 1, 2, 3). In all tests, students had to demonstrate their understanding of CVS, either by choosing an adequate design from a set of confounded and unconfounded experiments, correcting a confounded experiment, or identifying the independent and dependent variable in an unconfounded experiment. To ensure that the two posttests and follow-up were comparable, we used an anchor test. 6 anchor items in posttest 1 and 2 (4 of which were also anchor items of all three questionnaires) provide a baseline for an equating analysis. An anchor item consisted of an uniform description of an experiment (item stem) followed by the prompt to either

Task (1) choose an adequate design from a set of confounded and unconfounded experiments, e.g., “Which second experimental procedure does Amelie need? Mark the correct answer!”
Task (2) identify the independent and dependent variable in an unconfounded experiment, e.g., “What research question did Marggraf investigate by comparing Procedure 1 and Procedure 2?” or
Task (3) correct a confounded experiment, e.g., “Why is the planned experiment not the correct one to test Maren’s hypothesis?”

The tasks challenged the students to reason about the quality of others’ research – to evaluate an adequate design, hypotheses, measurements, data analysis and conclusions. Thus, students were tested with 16 anchor items (+ 2 non-anchored items) on the same scientific knowledge construct and skills at three different measuring points, in each case using two or three versions of Tasks (1) – (3) in 6 different experimental contexts (related to just a few content areas) (see Table 1).

**Table 1:** Anchor and non-anchored items of posttest 1, posttest 2, and follow-up.

Task	Anchor item	Posttest 1	Posttest 2	Follow-Up
(1) Choose an adequate design (AD) from a set of confounded and unconfounded experiments	1 Factors influencing the growth of beans	AD: Sunlight vs. no sunlight	AD: Water vs. no water	AD: Fertilizer vs. no fertilizer
	2 Factors influencing dragonfly larva’s hunting for prey	AD: Colored vs. transparent/white prey	AD: High number of experimental animals	AD: No feeding
(2) Identify the independent variable (IV) and dependent variable (DV) in an unconfounded experiment	3 Factors influencing the sugar production of sugar beets	IV: Temperature DV: Sugar production of sugar beet	IV: Care DV: Sugar production of sugar beet	IV: Soil type DV: Sugar production of sugar beet
	4 Factors influencing fish’s breathing in an aquarium	IV: Number of fishes in an aquarium DV: Fish breathing	IV: Temperature DV: Fish breathing	IV: Aquatic plant DV: Fish breathing
	5 Factors influencing woodlice’s habitat selection	IV: Darkness DV: Preferred habitat of woodlice	IV: Temperature DV: Preferred habitat of woodlice	
	Non-anchored factors influencing backswimmers‘ hunting for prey			IV: Visual stimulus DV: Reaction of backswimmers
(3) Correct a confounded experiment/ identify the disturbance variable (DI)	6 How light influences water fleas’ behavior	DI: Aquatic plant	DI: Feeding of a number of experimental animals	
	Factors influencing effervescent tablets’ release of CO_2_			DI: Water temperature

Given that the test items were formulated slightly differently at different measurement points, test scores can only be compared intertemporally to a limited extent, as any score differences might have also occurred due to the difference in formulation. As a result, the tests at different time points may have had different levels of difficulty. On that account, we adjusted the scores on posttest 2 to the scores on posttest 1 by means of a linear equating function in order to prove the equivalence of the test scores. This was done with the R package equate (Albano, 2016).

Difficulty adjustments of test scores can be conducted as long as the following two conditions are met: equivalence of content and similar statistical specifications between items. In this case, the test scores can be statistically adjusted and compared using the equating procedure (Kolen and Brennan, 2010). We did so using the single group design with data from the same sample.

Item difficulty, internal consistency and discrimination parameters were calculated using SPSS. Item difficulty was appropriate (*p = 0.*58–0.77) and the tests (α = 0.54–0.64) were found to be reliable for a comparing of groups (Lienert and Raatz, 1998). Further, the discrimination parameter were all above rit > 0.30, except of Item 4 and 5 (Posttest 1), Item 1 and 6 (Posttest 2; rit. > 0.21).

#### Learners’ Prerequisites

The students’ need for cognition (Preckel, 2014) and cognitive abilities (Heller and Perleth, 2000) were assessed using valid questionnaires (NFC: *p* = 3.27, α = 0.89, rit > 0.30; CA: *p* = 0.47, α = 0.90, rit > 0.30). the questionnaire for need for cognition contained 17 items (after 2 were excluded) measured on a five-point Likert scale, while the questionnaire for cognitive abilities for 6th graders measured figural inductive reasoning. It comprised 23 items (after 2 were excluded) with five possible answer options, of which only one Was correct, *p* = 0.46, α = 0.91, rit > 0.30. Thus, the students were asked to detect figural analogies (KFT 4–12+ R, Subtest N, Heller and Perleth, 2000). They were given 9 min to answer as many items as possible (see Supplementary Material: questionnaire for cognitive abilities). Immediately after the introductory session, a prior knowledge assessment test was conducted in order to determine individual differences in students’ scientific reasoning skills. the test contained 4 items, which were structured just like the items for posttest 1, 2 and follow-up. They comprised 3 anchor items and 1 non-anchored item (*p* = 0.58, α = 0.56, rit > 0.30) (see Table 2).

**Table 2:** Anchor and non-anchored items of pretest.

Task	Anchor item	Pretest
(1) Choose an adequate design (AD) from a set of confounded and unconfounded experiments	1 Factors influencing the growth of beans	AD: Clay vs. soil

(2) Identify the independent variable (IV) and dependent variable (DV) in an unconfounded experiment	2 Factors influencing dragonfly larva’s hunting of prey	IV: Size of prey DV: Reaction of the dragon fly
	
	5 Factors influencing woodlice’s habitat selection	IV: Humidity DV: Preferred habitat of woodlice
	
	Non-anchored Factors influencing backswimmers‘ hunting of prey	IV: Visual stimulus DV: Reaction of backswimmers

#### Learners’ Success in Self-Generation

Qualitative data encompassing all student responses to self-generation prompts in the inquiry-based environment, such as their experimental designs, methodological discussion, and the final cloze in their research workbooks, was also collected in order to verify treatment effects and analyze the influence of self-generation success on short-term and long-term retention. The data was scored on a scale with a maximum 33 credit points. For *experimental design*, the following factors were evaluated (each ranging between 0 and 2): identifying the independent and dependent variables, designing a controlled experiment by varying one independent variables and holding all untested variables constant across all conditions by identifying and controlling various biases and confounds, and determining the measurement intervals and number of experimental animals (water fleas). For *methodological discussion*, the following data was analyzed (ranging from 0 to 2): ensuring equal control conditions and the reason for it, the use of LED light and more than 10 water fleas as well as the reasons for this, preventing certain external influences (incidence of light, collisions with the desk, noise) and the reasons for this, and the need for and duration of a habituation period for the water fleas (for further information, see Supplementary Material: coding scheme).

An interrater reliability analysis using the Kappa statistic was performed to determine consistency among two independent raters. Interrater agreement was assessed on 1346 pairs of observations. The interrater reliability was found to be Cohen’s κ = 0.94 (*p* < 0.001). This indicates almost perfect agreement (Landis and Koch, 1977).

#### Learners’ Cognitive Load

The students’ perceived cognitive load (Cierniak et al., 2009; modified) was measured in both conditions immediately after the experimental unit. The questionnaire consisted of 5 items (after 1 was excluded) evaluated on a six-point Likert scale (1 = low to 6 = high) (*p* = 2.12, α = 0.60, rit > 0.30). Intrinsic and extraneous load were both measured via 2 items, e.g., “How hard was it for you to understand the experiment?” and “How hard was it for you to work with the research workbook?,” while germane load was determined using only 1 item, “How much effort did you need to put into learning today?” Item 6 “How strongly did you concentrate while learning today?” was excluded (see Supplementary Material: questionnaire for cognitive load).

### Data Analysis

Statistical analyses based on classical test theory were conducted using the software SPSS and Mplus (Muthén and Muthén, 2008) in order to identify differences between groups, learners of different abilities and the influences of learners’ characteristics on learning outcomes.

All results were significant at the 0.05 level unless otherwise stated. Pairwise comparisons were Bonferroni-corrected or Welch-adjusted. Partial eta squared ($\eta_{p}^{2}$) and Cohen’s *d* are reported as measures of effect size for all ANOVAs and all *t*-tests, respectively.

## Results

### Learning Outcome – Reading Versus Generating and Short-Term Versus Long-Term Retention (H1)

**FIGURE 1:**
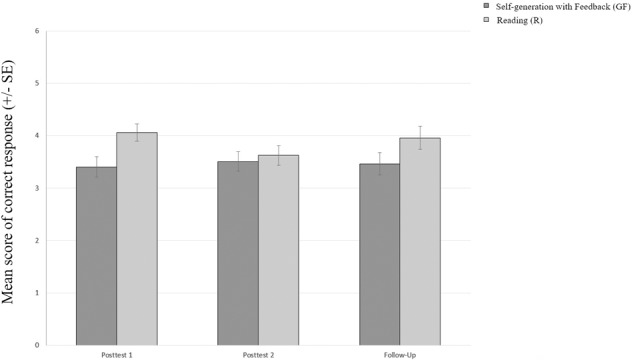
Mean score of correct responses by treatment and time.

The results were analyzed in a 2 (condition: self-generation vs. reading) x 3 (retention interval: immediate vs. after a 1-week delay vs. after a 4–6-week delay) ANOVA with repeated measures. There was no main effect of retention interval, but there was a main effect for condition, *F*(1,107) = 4.32, *p* = 0.040, $\eta_{p}^{2}$ = 0.039. Students in the reading condition performed better than students in the self-generation treatment. No significant interaction between retention interval and condition was found. A 2 × 2 repeated measures ANOVA with only two retention intervals was also calculated, for two reasons: First, eliminating the third measurement point increased statistical power, and second and more importantly, we could not control for external influences over the final 4–6 weeks. As we conducted the experiment under authentic school conditions, there was a high probability that the biology teachers in the participating classes continued the unit on inquiry-based learning in subsequent biology lessons, as they participated in our project for educational reasons. Ultimately, a 2 × 2 ANOVA revealed similar results: There was again no main effect of retention interval. This time, there were no statistically significant differences between the different conditions either. The interaction between retention interval and condition was again not significant, *F*(1,131) = 3.72, *p* = 0.056, $\eta_{p}^{2}$ = 0.028. But in accordance with our expectations, the (R) condition (P1: *M* = 4.06, *SD* = 1.31) outperformed the (GF) condition (P1: *M* = 3.41, *SD* = 1.62) at the first measurement point, (T1), *t*(125.58) = 2.58, *p* = 0.011, *d* = 0.44. However, the performance differences disappeared after a week (see Figure 1). These results suggest that the benefit of reading depends on the retention interval.

In contrast to the previous experiment on self-generation of content knowledge in inquiry-based learning (Kaiser and Mayer, unpublished), retention of scientific reasoning skills significantly declined over 1 week in the (R) condition, with a forgetting rate of 11%, (P2: *M* = 3.63, *SD* = 1.49), *t*(63) = 2.27, *p* = 0.027, *d* = 0.31), but remained stable in the (GF) condition over 1 week, with a forgetting rate of -3%, (P2: *M* = 3.51, *SD* = 1.55) (see Figure 1). Likewise, there was no difference between the treatments after a retention interval of 4–6 weeks.

The randomization controls identified no significant differences between conditions with respect to students’ demographic data, grades, need for cognition or cognitive abilities. These results suggest successful random treatment assignment.

### Learners’ Cognitive Load (H2)

**FIGURE 2:**
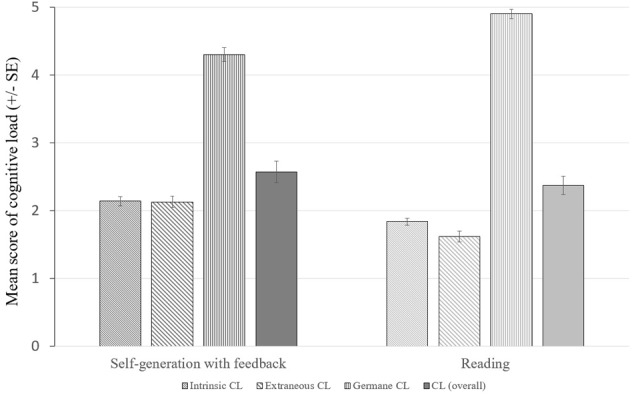
Mean score of (intrinsic, extraneous, germane, overall) cognitive load by treatment (self-generation with feedback (GF), reading (R)).

Overall cognitive load turned out to be significantly higher in the (GF) condition (*M* = 2.569, *SD* = 0.548) compared to the (R) condition (*M* = 2.369, *SD* = 0.408), *t*(125.27) = 2.29, *p* = 0.024, *d* = 0.39. Differential analyses revealed that intrinsic and extraneous load were significantly affected by self-generation, IL: (GF: *M* = 2.16, *SD* = 0.705, R: *M* = 1.84, *SD* = 0.643), *t*(131) = 2.64, *p* = 0.009, *d* = 0.45, EL: (GF: *M* = 2.14, *SD* = 0.865, R: *M* = 1.62, *SD* = 0.547), *t*(116.07) = 4.12, *p* < 0.001, *d* = 0.70 and accompanied by a significant reduction in germane load, (GF: *M* = 4.24, *SD* = 1.29, R: *M* = 4.94, *SD* = 1.08), *t*(131) = -3.32, *p* = 0.001, *d* = 0.58 (see Figure 2).

### Learners’ Success in Self-Generation (H3)

In order to assess the role of self-generation success, the experimental plan and appropriate discussion generated by the students was analyzed to ascertain how much information was successfully generated how often by each individual student (total score = 33; *M* = 17.5, *SD* = 6.13, *Mdn* = 19, *Max* = 27, *Min* = 0). A repeated measures ANOVA revealed that students with high success in self-generation (total score ≥ 19, Mediansplit) outperformed students with low self-generation success immediately after inquiry-based learning, and after a 1-week and 4–6-week delay, *F*(1,55) = 18.01, *p* < 0.001, $\eta_{p}^{2}$ = 0.247.

Comparing the test scores of only the highly successful students with students in the reading condition after a 1-week delay did reveal a significant difference (*t*(97) = 2.03, *p* = 0.045, *d* = 0.43). However, this is an unfair comparison, as it compares only the high achievers in the self-generation condition with the average of both high and low-performing students in the reading condition. To improve the comparison, we matched the highly successful students in the self-generation condition with a group of similar students in the reading condition by means of their grades in biology, mathematics and German. Again, the results were analyzed as a 2 (condition: self-generation vs. reading) × 3 (retention interval: immediate vs. after a 1-week delay vs. after a 4–6-week delay) ANOVA with repeated measures. There was no main effect of retention interval or condition. Nor was an interaction found between retention interval and condition. However, *post hoc* pairwise comparisons revealed a significant difference after a 1-week delay, (GF_highsuccess_: *M* = 4.23, *SD* = 1.26, R_match_: *M* = 3.50, *SD* = 1.50), *t*(65) = 2.15, *p* = 0.035, *d* = 0.53. However, although students in the self-generation condition outperformed students in the reading condition after a 1-week delay, the differences had dissipated after 4–6 weeks.

### Learners’ Prerequisites (H4)

**FIGURE 3:**
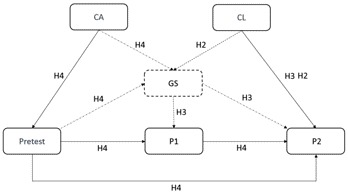
Theoretical path model of expected influences of learners’ prerequisites on short-term and long-term retention in both treatments. P1 = immediate test; P2 = 1-week-delayed test; Pretest = prior knowledge on scientific reasoning skills (CVS); CA = cognitive abilities; CL = cognitive load; GS = self-generation success in (GF) treatment; H1–4 = Hypothesis 1–4.

A manifest path model (see Figure 3) was used to analyze the complex interactions between variables concerning the learning process, the immediate and delayed tests, and learners’ prerequisites (Eid et al., 2017). This method was useful to apply due to our use of longitudinal survey data, which allows the causality of the relations between variables to be defined by referring to the point in time at which each measurement occurred. We considered multiple variables in the path model, specifying several dependent variables as well as dependent and independent variables at the same time (Eid et al., 2017). We also controlled for indirect correlations between variables (i.e., mediation effects) in the path model (Geiser, 2011).

The path model also allowed us to model and test our theoretical assumptions. We assumed that students who scored high on the pretest would also achieve higher scores on the immediate and delayed tests. This relation was expected to be valid for all students. Pretest outcomes should primarily be an effect of students’ cognitive abilities. Furthermore, the students for whom completing the tests involved high cognitive effort were expected to have lower test scores in the long run and vice versa (see Figure 3).

Table 3 presents the results of four path models. They were defined for the treatment group (GF), the treatment group without the variable for self-generation success (GF’), the control group (R), and both groups taken together (B). The models for the GF’, R and B groups had all the same variables and were directly comparable. The GF model had an extra variable that was only measured in this treatment group, namely self-generation success. This variable and its associations are highlighted in Figure 3 using dashed-pointed lines.

**Table 3:** Empirical path models of significant influences of learners’ prerequisites on short-term and long-term retention.

	Pretest				GS				P1				P2			
	GF	GF’	R	B	GF	GF’	R	B	GF	GF‘	R	B	GF	GF’	R	B
Pretest					0.45**	–	–	–	0.29**	0.45***	0.38**	0.34***	0.31**	0.40***		29^∗∗∗^
GS						–	–	–	0.36**				0.27*			
CA	0.33**	0.23**		0.23**		–	–	–								
CL						–	–	–			–0.35**	–0.21**	–0.23**	–0.26**		–0.17*
P1	–	–	–	–	–	–	–	–	–	–	–	–		0.21*		0.20*
*R*^2^	0.10	0.10	0.05	0.05	0.22*	–	–	–	0.35***	0.25***	0.40**	0.27***	0.46***	0.42***	0.07	0.35***

*P1 = immediate test; P2 = 1-week-delayed test; Pretest = prior knowledge on scientific reasoning skills (CVS); CA = cognitive abilities; CL = cognitive load; GS = self-generation success; GF: the model for the treatment condition (GF) with GS as a variable; GF’: the model for the treatment condition (GF) without GS as a variable, R: the model for the treatment condition (R); B: the combined model for both treatment conditions. ^∗^*p* < 0.05.*

The independent variables are on the left; the dependent variables for the four path models are on top. The fit values for all models were RMSEA < 0.01; *p*(RMSEA < 0.01), CFI = 1,000; SRMR < 0,001. All models were saturated with a degree of freedom of “0,” making it impossible to test the structure of covariance (Geiser, 2011). For each dependent variable, the amount of explained variance (*R*^2^) is reported.

In the GF model, two variables, self-generation success and pretest score, have the greatest influence on short-term (P1) and long-term (P2) retention. All beta values for these variables are moderate and range between 0.27 and 0.36. The variables for cognitive load and cognitive ability have no influence on short-term retention when controlling for self-generation success. Cognitive load only has an influence on long-term retention. Additionally, higher pretest scores predicted higher scores for self-generation success (β = 0.45^∗∗^).

In the treatment group without the self-generation success variable (GF‘), the pretest variable has the largest effect on test performance (β_P1_ = 0.45^∗∗∗^; β_P2_ = 0.40^∗∗∗^). There is also a correlation between scores on the first and the second posttest (β = 0.21^∗^). Cognitive abilities have a positive effect on pretest scores (β = 0.23^∗∗^), while cognitive load has a negative effect on posttest 2 scores (β = -0.26^∗∗^).

In the control group (R), the pretest (β = 0.38^∗∗^) and cognitive load (β = -0.35^∗∗^) variables were found to have an effect on posttest 1. None of the variables in the model had an impact on posttest 2.

All effects found in the separate R and GF‘ models can also be observed in group B (control and treatment conditions taken together). There is an effect of cognitive load on test scores at posttest 1 and 2 (β_P1_ = -0.21^∗∗^; β_P2_ = -0.17^∗^) and of cognitive ability on the pretest (β = 0.23^∗∗^). Moreover, a correlation between posttest 1 and posttest 2 can be observed (β = 0.20^∗^). Just as in the other models without the self-generation success variable, the pretest variable has the biggest effect on test scores (β_P1_ = 0.34^∗∗∗^; β_P2_ = 0.29^∗∗∗^) (see Table 3).

Students’ NFC and grades in Maths, German and Biology did not serve as a predictor for performance or test scores in any condition.

Parallel to the path analysis, multilevel analyses were conducted with the R packages lme4 (Bates et al., 2015), lmerTest and lsmeans (Lenth, 2016) in the R environment, version 3.4.4 (R Developmental Core Team, 2018), in order to determine differences between (R) and (GF) when controlling for group effects. The independent variable was the encoding format (generation with feedback, reading); the dependent variable was scores on the three tests measuring students’ achievement (P1, P2, P3). The groups students worked with were controlled for to remove variation in the dependent variable resulting from group effects. We still found significant differences between the two treatments immediately after inquiry (in favor of reading), P1: *Est.* = 0.663 (*SE* = 0.256)^∗^, and no differences in subsequent assessment measures, P2: *Est.* = 0.125 (*SE* = 0.313), P3: *Est.* = 0.438 (*SE* = 0.356), when controlling for group effects.

## Discussion

### Learning Outcomes – Reading Versus Generating and Short-Term Versus Long-Term Retention (Q1)

This study sought to analyze the distinctive role of self-generation of scientific reasoning skills and crucial requirements for the effectiveness of self-generation in inquiry-based learning. Therefore, we compared an inquiry activity that included the self-generation of scientific reasoning skills to an inquiry task that had students simply read information about the experimental design and an appropriate methodological discussion.

We hypothesized that students who engaged in the self-generation of scientific reasoning skills during inquiry would have an advantage on the delayed test, while students in the (R) condition would outperform students in the (GF) group on the immediate test. With respect to the focal skill of designing unconfounded experiments in simple contexts, the results for short-term retention confirmed our first hypothesis as well as other studies in which direct instruction was clearly superior to discovery learning in facilitating students’ acquisition of CVS after instruction (Chen and Klahr, 1999; Klahr et al., 2001; Klahr and Nigam, 2004). However, no differences were detected between the two treatments after a 1-week delay. Students in both treatment conditions achieved similar test scores after a period of 1 week had elapsed. However, whereas students in the (R) condition had already forgot almost 11%, retention did not decline in the self-generation treatment within a week. Thus, similar to the testing effect, self-generation reduces the rate of forgetting more than reading after 1 week (Roediger and Karpicke, 2006). However, no comparable results could be detected after 4–6 weeks.

These results indicate that inquiry-based instruction via self-generation prompts is less effective than direct instruction within inquiry when it comes to short-term retention, while from a long-term perspective, the self-generation of scientific reasoning skills is equal to the learning that results from direct instruction in terms of absolute values. However, with regard to the sustainability of knowledge, self-generation can be more effective and efficient in teaching students CVS. In this respect, the well-documented (long-term) effectiveness of inquiry-based learning (Furtak et al., 2012) can at least partially be ascribed to the generation effect (Slamecka and Graf, 1978; Schmidt and Bjork, 1992; Bertsch et al., 2007), although its full effectiveness only becomes apparent when self-generation is successful. As successful self-generation requires more assistance and guidance when the complexity of the learning content or the level of element interactivity (intrinsic cognitive load) becomes high (Chen et al., 2016), feedback plays a pivotal role in inquiry-based learning (Lazonder and Harmsen, 2016; Kaiser and Mayer, unpublished).

Although the encoding formats self-generation with feedback and reading led to different outcomes immediately after the inquiry task, they were equally effective after a 1-week delay. These findings – which were contrary to our expectations and previous laboratory results – can be explained by (1) the specific learning environment (inquiry-based learning), (2) the complexity of the learning content, and (3) the heterogeneity of the students.

(1) An important aspect of this study is that it was carried out in a natural setting. Consequently, students in both treatment conditions were instructed to conduct experiments (lab work) via a hands-on activity. This may have overshadowed the learning differences between the two conditions. Despite being aware of this potential bias, we had made a deliberate choice in favor of a hands-on activity in the interest of authenticity (Hmelo-Silver et al., 2007). In retrospect, this decision may have been enough for students to generate scientific reasoning skills even when they were not prompted to do so (Metcalfe and Kornell, 2007). Ultimately, even an apparently passive task on the instructional level can induce students to actively generate information on the cognitive level (Renkl, 2015), especially when they find themselves in an active learning environment. Renkl further points out that there is no perfectly pure form of inquiry-based learning in the sense of a minimum or maximum generation requirement (Renkl, 2009). Even receptive forms of learning like reading require connections to be generated on the basis of the content of the text and the learner‘s prior knowledge in order to achieve an understanding of the text (Renkl, 2015). A further aspect of the natural inquiry setting was the collaborative form of learning (teamwork) – which is a common practice in conventional lessons – causing communication among students in the two conditions about the information they had generated and read, respectively. In contrast to laboratory studies – which rely on the individual completion of generation or reading tasks – it cannot be guaranteed that all students successfully generate all information in a natural setting. However, in the end this is a central prerequisite for the generation effect (see section “Learners’ Success in Self-Generation”).
(2) Finally, it must be considered that scientific learning content is always complex and conceptual, whereas the generation effect has been detected in simple and low coherent contents (word pairs, antonyms, rhymes, etc.).
(3) The heterogeneity of the students is a crucial element of natural settings. Low and high-knowledge students profit very differently from both conditions (see Sections “Learners’ Success in Self-Generation” and “Learners’ Prerequisites”).

Therefore, it may be concluded that the generation effect does not appear in the context of a natural, curriculum-based and thus complex setting (like inquiry-based learning) with the same strength as in laboratory studies that rely on simple learning material and a more homogenous group of probands when self-generation success is not obtained.

### Learners’ Cognitive Load (Q2)

The differences and similarities in learning outcomes in the two treatment conditions and in both tests (immediate and delayed) can partially be explained by comparing the students‘ perceived cognitive load and interpreting the influences of student prerequisites on learning outcomes in the path model:

(1) Authentic learning situations in science education entail a high element interactivity and thus a high cognitive load. Students‘ intrinsic and extraneous cognitive load were lower in the (R) treatment condition than in the (GF) group. In the former group, causal relationships were explicitly provided and did not need to be generated or fixed in writing in the column provided in the research workbook. Thus, these students could benefit from a significantly higher germane cognitive load, which facilitated their understanding (Paas et al., 1994; van Merriënboer and Sweller, 2005).
(2) However, although the comparatively lower cognitive load in the reading group had a positive short-term effect on retention, while self-generation briefly increased learners’ cognitive load and impeded their learning process, this advantage did not hold in the long-run. This was in accordance with our expectations and previous findings (Bjork and Bjork, 2011).
(3) The significantly higher cognitive load of students in the self-generation condition during the learning unit had a measurable long-term (negative) effect. This could explain why we failed to find the expected GF advantage during the second measurement point.

Thus, for inquiry learning, it can be concluded that even in the long run, the retention of generated information is influenced by a perceived high cognitive load during knowledge acquisition.

### Learners’ Success in Self-Generation (Q3)

A path analysis revealed that performance success in self-generation turned out to be a reliable predictor of learning outcomes, in contrast to Richland and colleagues’ findings (Richland et al., 2007). Thus, learners who achieved high self-generation scores were able to retrieve more information immediately after instruction, and particularly after a 1-week delay, than low achievers. This was partly due to their significantly lower intrinsic cognitive load in contrast to their unsuccessful peers. These results underline the fact that cognitive processes during the generation of apparently complex knowledge differ from processes during the generation of apparently simple material (Chen et al., 2016). In the end, students with high self-generation success – and thus low intrinsic cognitive load – were able to store and retrieve more information than students with high intrinsic cognitive load and low self-generation success. A comparison of highly successful students in the self-generation condition with an equal number of students in the reading conditions matched according to grades in biology, mathematics and German revealed a generation effect with a medium effect size of *d* = 0.53. This result supports Foos’ assertion that the generation effect occurs only for successfully generated information (Foos et al., 1994). Information that was generated incorrectly or not at all by students in the self-generation treatment could not successfully be integrated into an existing knowledge construct, and thus correctly stored in long-term memory. As a result, the students failed to retrieve this information immediately and after a 1-week-delay. Thus, designing the learning environment in a way that facilitates students’ generation of correct information seems to be an essential condition for effective self-generation in a natural setting – like inquiry. This can be achieved by ensuring sufficient guidance and prior knowledge.

Given that a preexisting knowledge base is an important requirement for success in self-generation (Chen et al., 2016), it is not surprising that success in self-generation was moderated by students’ prior knowledge of scientific inquiry skills.

### Learners’ Prerequisites (Q4)

Our results highlight the important role of prior knowledge, self-generation success and cognitive load for the effectiveness of self-generation for long-term retention in a natural setting. The path model revealed that performance after reading benefited from low cognitive load and high prior knowledge, while students’ prior knowledge and self-generation success were decisive for short-term retention after self-generation with feedback.

Students’ long-term retention of read information was not moderated by any of the tested variables (KFT, CL, grades) – not even by prior knowledge. In contrast, prior knowledge, cognitive load and self-generation success had a crucial influence on the long-term retention of scientific reasoning skills in the (GF) condition. High prior knowledge improved self-generation success and test performance, while low prior knowledge led to poor performance and poor retention. In turn, students’ prior knowledge was influenced by their cognitive abilities. This highlights the importance of the ability to link preexisting knowledge with new information, generate new inferences and therefore reduce a large number of interacting elements to only a few chunks. Thus, expertise – cognitive abilities and existing knowledge – can have a substantial impact on element interactivity and cognitive load (Chen et al., 2016), even though no direct influence of expertise on cognitive load was found in this study.

Apart from this, it could be clearly demonstrated that students with low prior knowledge were less likely to benefit from self-generation, because such students had fewer opportunities to generate correct information and procedures and thus achieve high self-generation success. Since a novice could hardly be expected to immediately be able to identify independent and dependent variables, control for various biases and confounds, determine the measurement intervals and number of measurements, solutions for novices consist of many elements. And a high element interactivity is always associated with a very high intrinsic cognitive load. Hence, students with disconnected knowledge on scientific reasoning tended to isolate new information and quickly forget the information they had learned, while students who developed integrated understandings of CVS were able to add new information using the knowledge integration process (Clark and Linn, 2003). This in turn led to higher performance during the learning process – in contrast to low-knowledge students, who were not able to diminish their intrinsic cognitive load by recognizing the appropriate variables (e.g., Siegler, 1991; Shrager and Siegler, 1998).

## Conclusion

In the end, this study can broaden the research base on the generation effect. We were able to demonstrate that the long-term effectiveness of self-generation in a natural setting depends on several critical, interrelated factors: students’ self-generation success, prior knowledge and cognitive load. Thus, self-generation with feedback represents a desirable difficulty in the educational context of inquiry-based learning under certain conditions. Students with low prior knowledge tend to become mentally overstrained, when trying to generate new inference in a natural setting like inquiry-based learning. As they are unable to handle the information overload, reading represents the better learning strategy since causal relationships are explicitly described. In the short run, the retention of read information can be influenced through adaptation to students’ prior knowledge and a consequent reduction of cognitive load. However, it provides no advantage compared to self-generation and has no influence in the long run. Self-generation even leads to better long-term learning outcomes when self-generation success can be ensured. For this reason, integrating feedback into inquiry-based learning represents a helpful strategy for improving long-term retention because it is related to self-generation success (Kaiser and Mayer, unpublished). At the same time, several other regulatory factors (e.g., prior knowledge, cognitive load) can be consciously deployed to increase learning outcomes.

By stressing regulatory influences, this research also has clear educational implications: Instructors should analyze where students stand with regard to their expertise development in inquiry. Detecting students’ level of expertise development allows instruction and additional support to be flexibly adapted to individual students’ needs. Reading represents a better learning strategy for students with low prior knowledge, whereas a high level of expertise in inquiry facilitates the effectiveness of self-generation. Knowing students’ level of expertise and providing them with appropriate instruction helps students reduce their cognitive load and allows new information to properly be linked with previous knowledge. Promoting the process of knowledge integration leads to higher self-generation success. Ultimately, it is self-generation success that is the key to higher long-term retention (compared to reading).

Future studies should test how self-generation success can be increased in inquiry-based learning. Two regulatory factors can be analyzed: the need for a sufficient amount of prior knowledge (e.g., worked examples) as a subsequent factor and an appropriate amount of assistance (e.g., scaffolding) as an accompanying factor for self-generation.

## Data Availability Statement

The raw data supporting the conclusions of this manuscript will be made available by the authors, without undue reservation, to any qualified researcher.

## Ethics Statement

For the reported study, no ethics approval was required per the guidelines of the University of Kassel or national guidelines. We conducted the study in line with the recommendations of the ethics committee of the University of Kassel and with approval of the “Ministry of Education and Cultural Affairs, Hesse, Germany (Hessisches Kultusministerium)” (cf. Education Act of Hesse, section 84). The parents of all participants gave written informed consent in accordance with the Declaration of Helsinki.

## Author Contributions

JM developed the basic idea for the present study and supervised the project. IK was responsible for the study design and data collection procedure, conducted the analyses (SPSS), and wrote the manuscript in consultation with JM. DM contributed to the analysis and writing of the path modeling and equating procedures. All authors contributed to the final version of the manuscript.

## Conflict of Interest Statement

The authorsdeclare that the research was conducted in the absence of any commercial or financial relationships that could be construed as a potential conflict of interest. The handling Editor declared a shared affiliation, though no other collaboration, with the authors at time of review.

## Footnotes

1 The German school system is a three-tiered system in which students are divided into three different tracks depending on their cognitive abilities and educational goals: (1) *Gymnasium* for high academic achievers throughout elementary school headed for college, (2) *Realschule* for the next step down, students headed for average or better white-collar positions, and (3) *Hauptschule* for the bottom tier, generally aimed at the trades and blue-collar jobs.

2 It is therefore often measured in research on inquiry learning (e.g., Klos et al., 2008; Nehring et al., 2014) in order to predict learning capacity – for example via KFT 4–12 + R, Subtest N (Heller and Perleth, 2000).

3 In some German federal states, Grades 5 and 6 are combined to form educational units known as the “promotion level” (*Förderstufe*) before students begin one of the three secondary school tracks in Grade 7: Hauptschule, Realschule or Gymnasium.

## Supplementary Material

FIGURE S1 Questionnaire cognitive abilities.

FIGURE S2 Questionnaire_need for cognition.

FIGURE S3 Questionnaire_cognitive load.

FIGURE S4 Pretest.

FIGURE S5 Posttest_1.

FIGURE S6 Posttest_2.

FIGURE S7 Posttest_3.

FIGURE S8 Coding_scheme.

FIGURE S9 Research workbook_GF.

FIGURE S10 Research workbook_R.

FIGURE S11 Supervisor workbook for instructions_GF.

FIGURE S12 Supervisor workbook for instructions_R.

TABLE S1 Example_anchor item.

TABLE S2 Example 1, Example 2, Example 3, and Example 4.
