# Supplementary material for: Self-Generation in the Context of Inquiry-Based Learning
Source: Front Psychol. 2018 Dec 13;9:2440. doi: 10.3389/fpsyg.2018.02440 (PMC6315139; doi:10.3389/fpsyg.2018.02440)
Supplement: FIGURE S1 — Questionnaire cognitive abilities. [file Image_1.pdf]

Trage hier deinen Namen ein: ______________________________________________

✂-----------------------------------------------------------------------------------------------------------

*(Dieser Teil wird später abgetrennt)*

In diesem Fragebogen siehst du eine Reihe von Figuren. Du sollst herausfinden, was an den Figuren oder Zeichnungen gleich ist. Bitte bearbeite die Aufgaben so, wie in den Beispielen gezeigt:

**BEISPIEL 1**

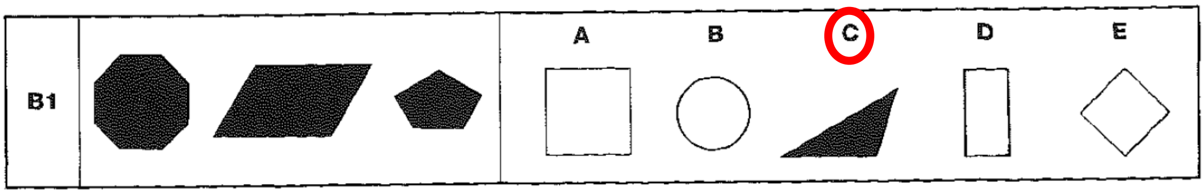


**BEISPIEL 2**

**Welche Antwort ist hier richtig?**

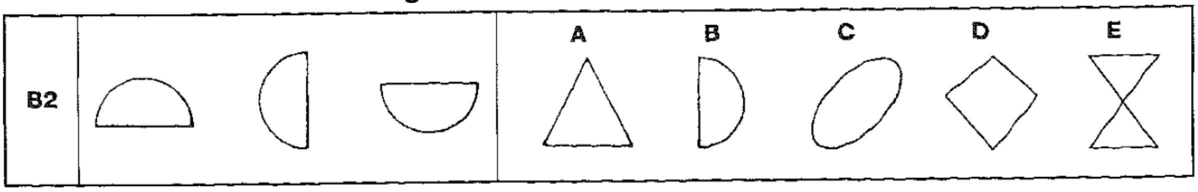


**BEISPIEL 3**

**Und hier?**

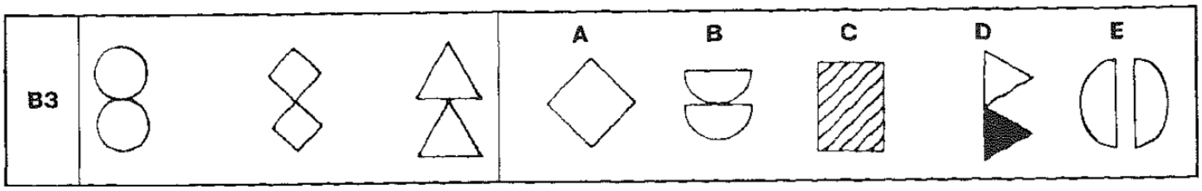

| | | |
|---|---|---|
| 1 | 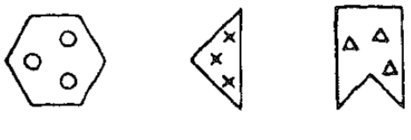 | 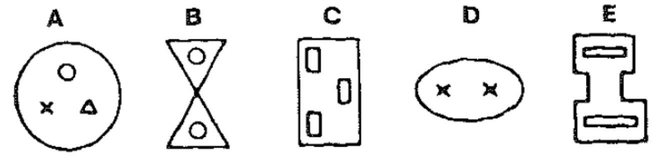 |
| 2 | 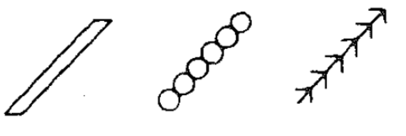 | 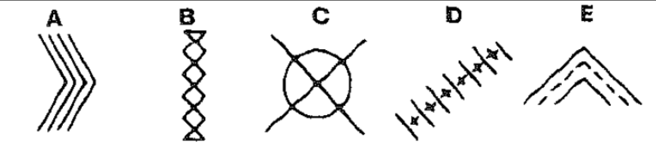 |
| 3 | 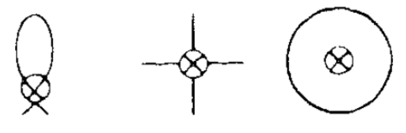 | 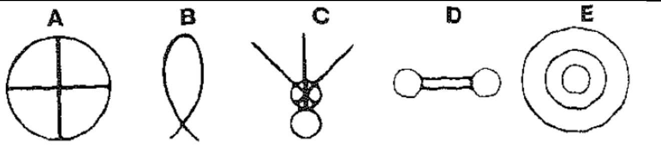 |
| 4 | 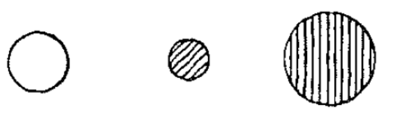 | 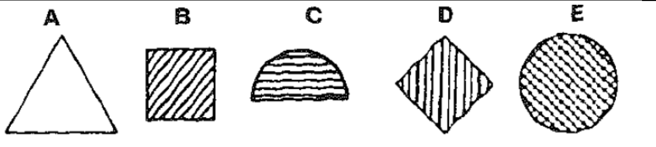 |
| 5 | 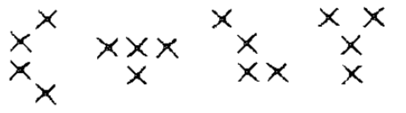 | 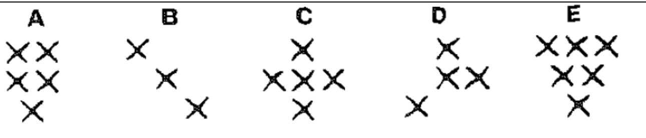 |
| 6 | 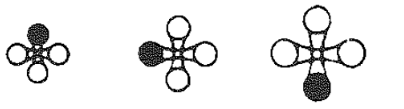 | 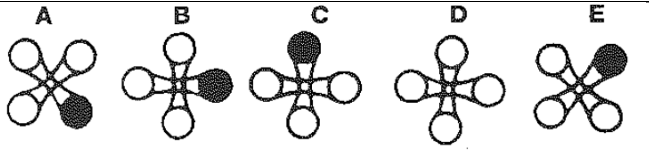 |
| 7 | 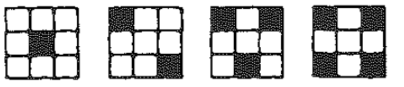 | 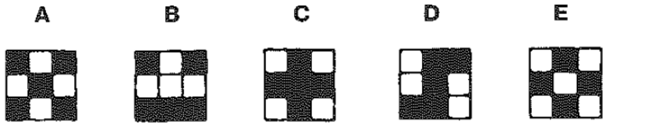 |
| 8 | 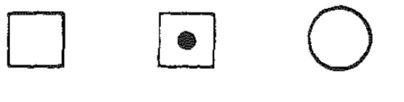 | 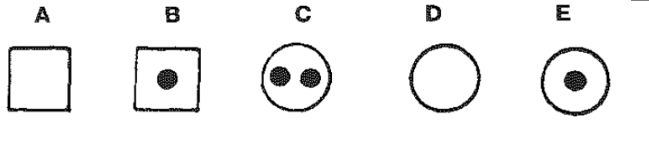 |
| 9 | 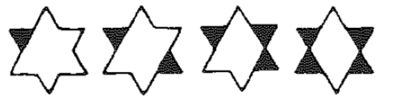 | 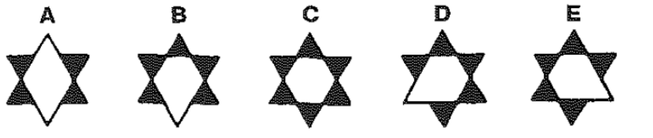 |
| 10 | 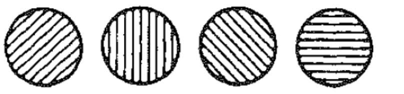 | 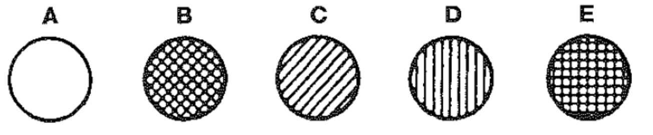 |
| 11 | 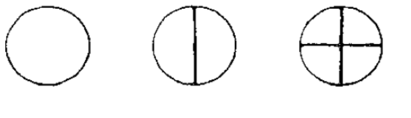 | 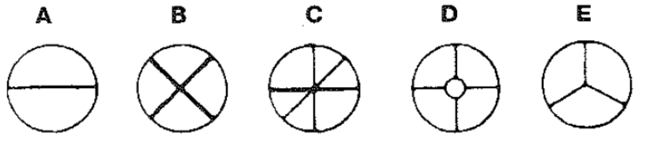 |

| | | |
|---|---|---|
| 12 | 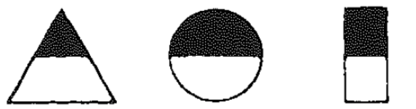 | A B C D E 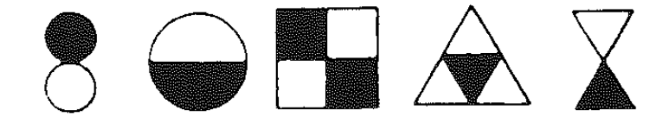 |
| 13 | 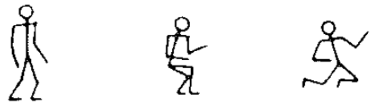 | A B C D E 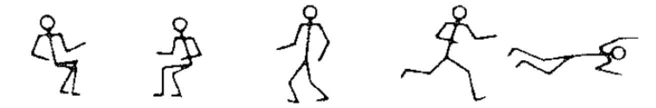 |
| 14 | 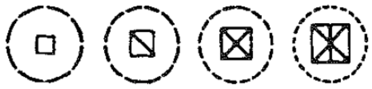 | A B C D E 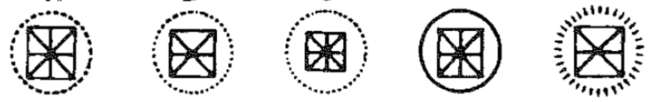 |
| 15 | 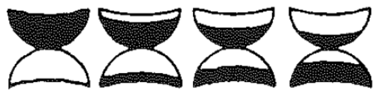 | A B C D E 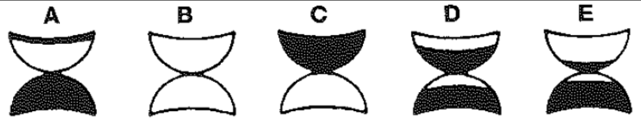 |
| 16 | 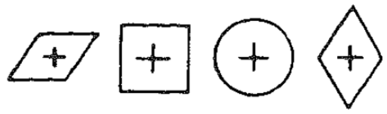 | A B C D E 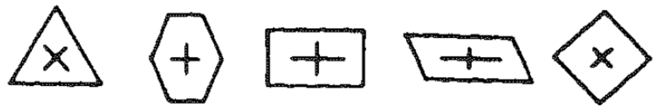 |
| 17 | 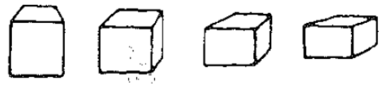 | A B C D E 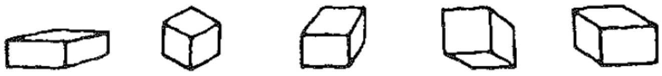 |
| 18 | 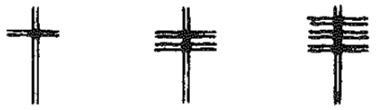 | A B C D E 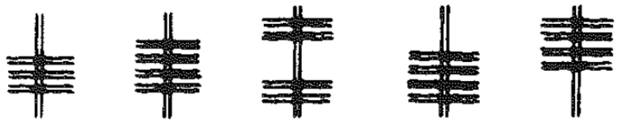 |
| 19 | 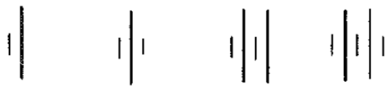 | A B C D E 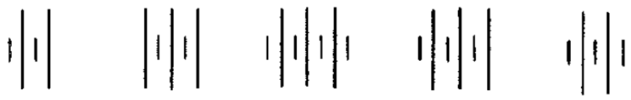 |
| 20 | 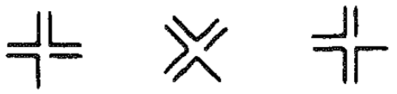 | A B C D E 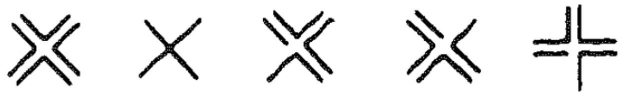 |
| 21 | 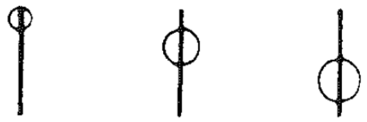 | A B C D E 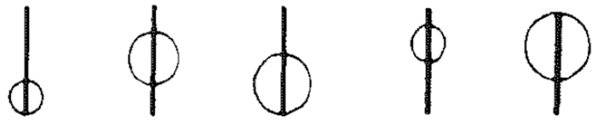 |
| 22 | 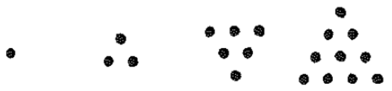 | A B C D E 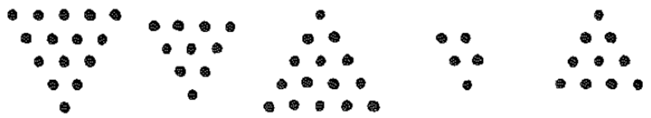 |

| | | |
|---|---|---|
| 23 | 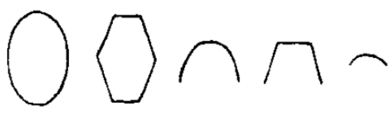 | 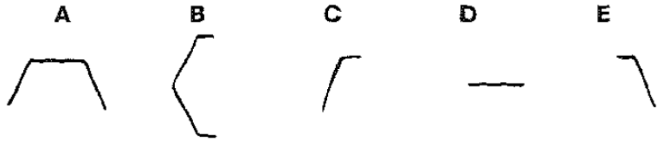 |
| 24 | 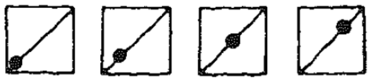 | 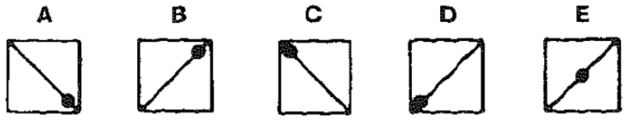 |
| 25 | 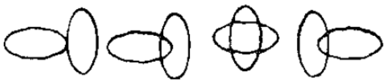 | 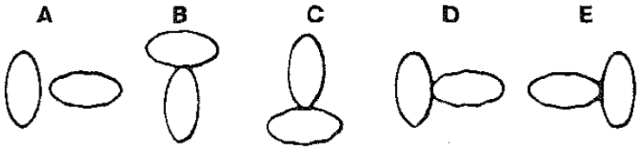 |

---

## NUN HAST DU ES FAST GESCHAFFT!

## VIELEN DANK FÜR DEINE MITARBEIT!

---
