# Supplementary material for: Self-Generation in the Context of Inquiry-Based Learning
Source: Front Psychol. 2018 Dec 13;9:2440. doi: 10.3389/fpsyg.2018.02440 (PMC6315139; doi:10.3389/fpsyg.2018.02440)
Supplement: FIGURE S2 — Questionnaire_need for cognition. [file Image_2.pdf]

Trage hier deinen Namen ein: ____

✂--

(*Dieser Teil wird später abgetrennt*)

ZUM SCHLUSS.....

noch ein Fragebogen. Mit den Fragebogen erfassen wir, wie sehr euch Denken Spaß bereitet. Eure Antworten sind für das Projekt der Universität Kassel sehr wichtig. Bearbeitet daher alle Fragen eigenständig und sehr sorgfältig.

Hier findest du 19 Aussagen, bei denen du für dich selbst einschätzen sollst, wie sehr dir Denken Spaß bereitet. Mache pro Aussage immer nur ein Kreuz.

1. Es macht mir viel Spaß, mir Lösungen für Probleme auszudenken.

☐	☐	☐	☐	☐
1	2	3	4	5
trifft überhaupt nicht zu				trifft ganz genau zu

2. Ich würde lieber eine wichtige Aufgabe lösen, die schwierig ist und Nachdenken erfordert, als eine Aufgabe, die zwar wichtig ist, aber nicht viel Nachdenken erfordert.

☐	☐	☐	☐	☐
1	2	3	4	5
trifft überhaupt nicht zu				trifft ganz genau zu

3. Ich mag Situationen, in denen ich mit gründlichem Nachdenken etwas erreichen kann.

☐	☐	☐	☐	☐
1	2	3	4	5
trifft überhaupt nicht zu				trifft ganz genau zu

4. Ich mag keine Situationen, in denen ich mich auf mein Denken verlassen muss, um etwas zu erreichen.

☐	☐	☐	☐	☐
1	2	3	4	5
trifft überhaupt nicht zu				trifft ganz genau zu

5. Für mich ist es besonders schön, wenn ich eine wichtige Aufgabe erledigt habe, die viel Nachdenken erfordert hat.

☐	☐	☐	☐	☐
1	2	3	4	5
trifft überhaupt nicht zu				trifft ganz genau zu

auf der nächsten Seite geht’s weiter...

6. Ich würde lieber etwas tun, bei dem ich wenig nachdenken muss, als etwas, bei dem ich viel nachdenken muss.

☐ 1 trifft überhaupt nicht zu | ☐ 2 | ☐ 3 | ☐ 4 | ☐ 5 trifft ganz genau zu

7. Ich mag es nicht, angestrengt und stundenlang nachzudenken.

☐ 1 trifft überhaupt nicht zu | ☐ 2 | ☐ 3 | ☐ 4 | ☐ 5 trifft ganz genau zu

8. Ich denke nur nach, wenn ich muss.

☐ 1 trifft überhaupt nicht zu | ☐ 2 | ☐ 3 | ☐ 4 | ☐ 5 trifft ganz genau zu

9. Nachdenken macht mir keinen Spaß.

☐ 1 trifft überhaupt nicht zu | ☐ 2 | ☐ 3 | ☐ 4 | ☐ 5 trifft ganz genau zu

10. Ich mag keine Situationen, in denen ich intensiv über etwas nachdenken muss.

☐ 1 trifft überhaupt nicht zu | ☐ 2 | ☐ 3 | ☐ 4 | ☐ 5 trifft ganz genau zu

11. Ich habe es gern, wenn mein Leben voller kniffliger Aufgaben ist, die ich lösen muss.

☐ 1 trifft überhaupt nicht zu | ☐ 2 | ☐ 3 | ☐ 4 | ☐ 5 trifft ganz genau zu

12. Ich mag komplizierte Probleme lieber als einfache Probleme.

☐ 1 trifft überhaupt nicht zu | ☐ 2 | ☐ 3 | ☐ 4 | ☐ 5 trifft ganz genau zu

auf der nächsten Seite geht's weiter…

13. Gibt es ein Problem, dann genügt es mir, einfach die Lösung zu kennen: Mir ist es nicht wichtig, die Gründe für die Lösung des Problems zu verstehen.

☐ 1 ☐ 2 ☐ 3 ☐ 4 ☐ 5

trifft überhaupt nicht zu — trifft ganz genau zu

14. Mir genügt, dass etwas funktioniert und mir ist egal, wie oder warum es funktioniert.

☐ 1 ☐ 2 ☐ 3 ☐ 4 ☐ 5

trifft überhaupt nicht zu — trifft ganz genau zu

15. Ich sage mir oft, dass man gut und lange nachdenken muss, um die beste Lösung für ein Problem zu finden.

☐ 1 ☐ 2 ☐ 3 ☐ 4 ☐ 5

trifft überhaupt nicht zu — trifft ganz genau zu

16. Ich erledige gerne Aufgaben, bei denen man viel nachdenken muss.

☐ 1 ☐ 2 ☐ 3 ☐ 4 ☐ 5

trifft überhaupt nicht zu — trifft ganz genau zu

17. Ich bin jemand, der sehr gerne nachdenkt.

☐ 1 ☐ 2 ☐ 3 ☐ 4 ☐ 5

trifft überhaupt nicht zu — trifft ganz genau zu

18. Ich denke gerne über ein Problem nach, selbst wenn ich weiß, dass mein Nachdenken an dem Problem nichts ändern wird.

☐ 1 ☐ 2 ☐ 3 ☐ 4 ☐ 5

trifft überhaupt nicht zu — trifft ganz genau zu

19. Wenn ich mir in den Kopf setze, die Lösung für ein schwieriges Problem zu finden, schaffe ich das auch oft.

☐ 1 ☐ 2 ☐ 3 ☐ 4 ☐ 5

trifft überhaupt nicht zu — trifft ganz genau zu

NUN HAST DU ES GESCHAFFT! VIELEN DANK FÜR DEINE MITARBEIT!
