# Supplementary material for: Self-Generation in the Context of Inquiry-Based Learning
Source: Front Psychol. 2018 Dec 13;9:2440. doi: 10.3389/fpsyg.2018.02440 (PMC6315139; doi:10.3389/fpsyg.2018.02440)
Supplement: FIGURE S3 — Questionnaire_cognitive load. [file Image_3.pdf]

Trage hier deinen Namen ein: ____

✂--

(Dieser Teil wird später abgetrennt)

ZUNÄCHST noch ein paar Fragen, die uns helfen, das FLOX-Projekt zu bewerten. Hier sollst du einschätzen, wie anstrengend und anspruchsvoll das Experimentieren in deinem Forscherteam heute war. Mache pro Aussage immer <u>NUR EIN</u> Kreuz, für das, was am ehesten auf dich zutrifft:

1. Wie schwer war es für dich das Experiment zu verstehen?

☐	☐	☐	☐	☐	☐
1<br>einfach	2	3	4	5	6<br>sehr schwer

2. Wie schwer war es für dich wie ein Forscher zu arbeiten?

☐	☐	☐	☐	☐	☐
1<br>einfach	2	3	4	5	6<br>sehr schwer

3. Wie schwer war es für dich mit dem Forscherheft zu arbeiten?

☐	☐	☐	☐	☐	☐
1<br>einfach	2	3	4	5	6<br>sehr schwer

4. Wie schwer war es für dich den Arbeitsaufträgen im Forscherheft zu folgen?

☐	☐	☐	☐	☐	☐
1<br>einfach	2	3	4	5	6<br>sehr schwer

5. Wie sehr musstest du dich heute beim Lernen anstrengen?

☐	☐	☐	☐	☐	☐
1<br>Wenig	2	3	4	5	6<br>sehr

6. Wie stark hast du dich heute beim Lernen konzentriert?

☐	☐	☐	☐	☐	☐
1<br>wenig	2	3	4	5	6<br>sehr

NUN HAST DU ES FAST GESCHAFFT! VIELEN DANK FÜR DEINE MITARBEIT!

Zum Schluss….

noch drei kurze Fragen, um einzuschätzen, wie schwierig du die Aufgaben in diesem Testheft findest. Bitte versuche, dich so genau wie möglich einzuschätzen. Mache immer nur ein Kreuz pro Frage.

1. Wie schwierig war es für dich die Aufgaben zu verstehen?

☐	☐	☐	☐	☐	☐
1	2	3	4	5	6
einfach					sehr schwierig

2. Wie schwierig war es für dich mit diesem Testheft zu arbeiten?

☐	☐	☐	☐	☐	☐
1	2	3	4	5	6
einfach					sehr schwierig

3. Wie sehr hast du dich bei der Bearbeitung der Aufgaben angestrengt?

☐	☐	☐	☐	☐	☐
1	2	3	4	5	6
Wenig					sehr

NUN HAST DU ES FAST GESCHAFFT! VIELEN DANK FÜR DEINE MITARBEIT!
