# Supplementary material for: Self-Generation in the Context of Inquiry-Based Learning
Source: Front Psychol. 2018 Dec 13;9:2440. doi: 10.3389/fpsyg.2018.02440 (PMC6315139; doi:10.3389/fpsyg.2018.02440)
Supplement: FIGURE S4 — Pretest. [file Image_4.pdf]

Liebe Schülerinnen und Schüler,

Im Folgenden erhaltet ihr Aufgaben, um zu überprüfen, wie gut ihr euch mit wissenschaftlichen Experimenten bereits auskennt. Versucht die Aufgaben so gut es geht zu lösen. Es ist sehr wichtig, dass ihr euch beim Bearbeiten der Aufgaben große Mühe gebt. Bitte beantwortet jede Aufgabe selbständig und so gut ihr könnt.

Bevor ihr jedoch gleich die Fragen beantwortet, brauchen wir noch ein paar Informationen von euch:

Personendaten

Geschlecht	weiblich ☐		männlich ☐
Alter	Jahre		
Schulform	Gymnasium ☐	Realschule ☐	Hauptschule ☐
Noten im letzten Zeugnis	Biologie	Mathematik	Deutsch
Warst du mit deiner Klasse schon bei FLOX?	☐ Nein	Ja : ☐ Wasserfloh	☐ Achatschnecken

06	12	06			

(*hier müsst ihr nichts eintragen*)

Hier findest du kurze Aufgaben zur Großlibellenlarve und zum Experimentieren. Einige der Aufgaben werden dir sicherlich leicht fallen, andere sind etwas schwerer.
Falls du dir bei einer Aufgabe unsicher sein solltest, versuche trotzdem eine Antwort zu geben.

Bitte bearbeitet die Aufgaben so, wie es in den folgenden Beispielen gezeigt wird.

Bei Aufgaben wie in BEISPIEL 1 sollst du immer **NUR EIN** Kreuz setzen.

BEISPIEL 1

Wasserflöhe besitzen ….
✎ Kreuze an!

- ☐ ein Antennenpaar
- ☒ zwei Antennenpaare
- ☐ drei Antennenpaare
- ☐ vier Antennenpaare

Falls du es dir anders überlegt hast und deine Antwort ausbessern möchtest, dann mache dies folgendermaßen:

Wasserflöhe besitzen ….
✎ Kreuze an!

- ☐ ein Antennenpaar
- ■ zwei Antennenpaare
- ☒ drei Antennenpaare
- ☐ vier Antennenpaare

Bei 2 Aufgaben in diesem Heft musst du kurze Antwortsätze formulieren. Diese Aufgaben sehen aus wie **BEISPIEL 2**:

BEISPIEL 2

Warum gehören Wasserflöhe zu den Krebstieren und nicht zu den Insekten (wie Flöhe)?

Erkläre in 2-3 Sätzen

Insekten besitzen nur 6 Beine, Wasserflöhe haben aber 10.
Sie besitzen keine harte Schale.

Nun kannst du umblättern und mit der Bearbeitung der Aufgaben beginnen

Versuche mit Großlibellenlarven

Großlibellenlarven leben im Teich und fressen Mückenlarven, Wasserflöhe, ja sogar Kaulquappen. Prof. Plankton möchte herausfinden, wodurch Großlibellenlarven bei ihrem Beutefang beeinflusst werden können. Dazu setzt er zwei unterschiedliche Attrappen(Nachbildungen) einer Mückenlarve in ein Wasserbecken. Dann gibt er zehn Großlibellenlarven hinein und beobachtet:

Prof. Planktons Versuchsansatz

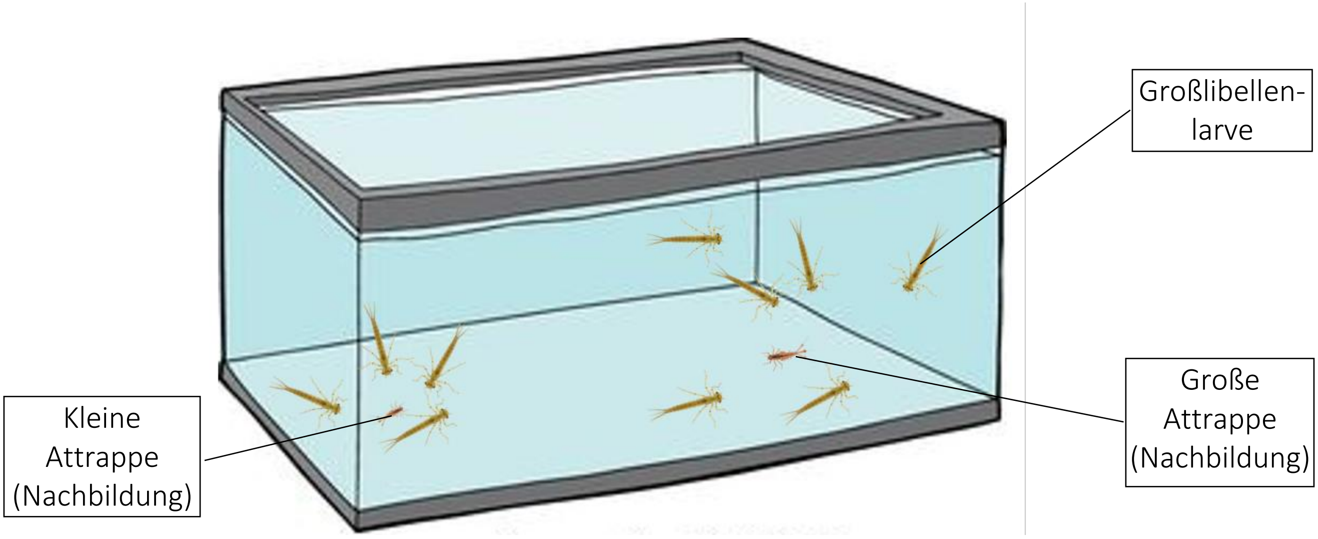

Aufgabe 1

Welche Vermutung möchte Prof. Plankton überprüfen? *(P_Gro_G_MC)*

✎ **Kreuze an!**

☐ Großlibellenlarven bevorzugen Kaulquappen als Beute.

☐ Großlibellenlarven bevorzugen rote Beutetiere.

☐ Großlibellenlarven bevorzugen große Beutetiere.

☐ Großlibellenlarven bevorzugen Mückenlarven als Beute.

Aufgabe 2

✎ **Erkläre in 1-2 Sätzen, warum Prof. Plankton mehrere Großlibellenlarven für sein Experiment nutzt.**

(P_Gro_G_O)

Pflanzenwachstum

Amelie möchte herausfinden, welcher Boden für das Wachstum von Bohnen am besten geeignet ist. Sie vermutet, dass Bohnen in Gartenerde besser wachsen als in Lehmboden. Um ihre Vermutung zu überprüfen, nimmt sie zwei Töpfe und befüllt sie mit zwei Arten von Boden. Sie pflanzt mehrere Bohnen in beide Töpfe und stellt sie in ihrem Zimmer auf die Fensterbank über die Heizung. Amelie weiß, dass Pflanzen Wasser und Mineralstoffe für ihr Wachstum benötigen. Daher gießt und düngt sie die Bohnen wöchentlich.

Amelies Versuchsansätze

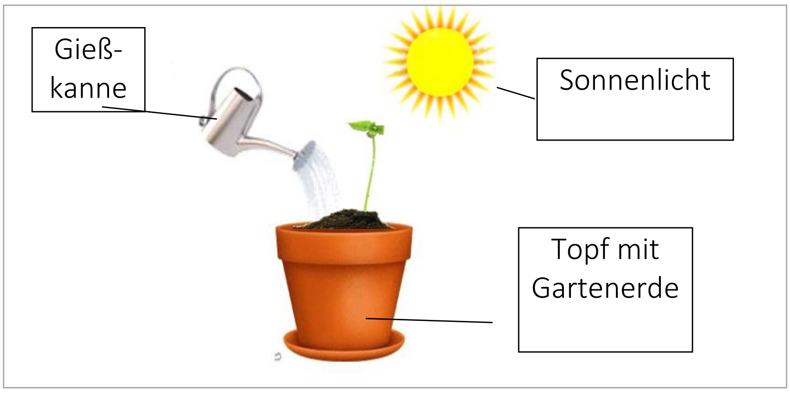

Aufgabe 3

Welchen zweiten Versuchsansatz benötigt Amelie? ✎ Kreuze an! *(P_Pfl_B_MC)*

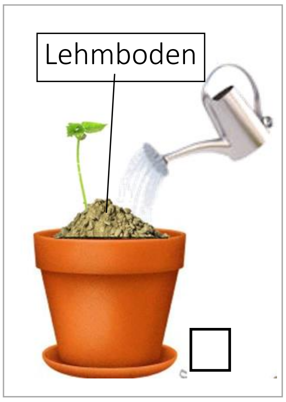

☐

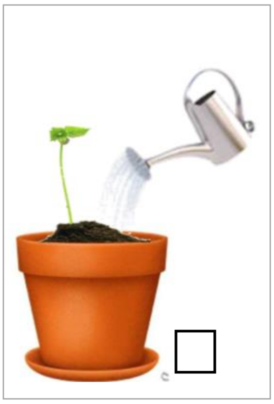

☐

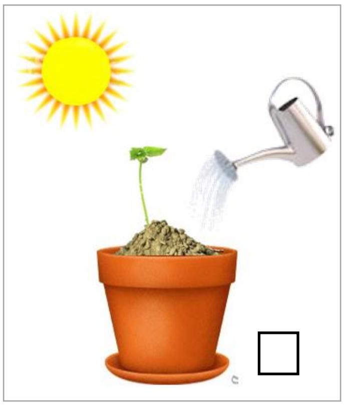

☐

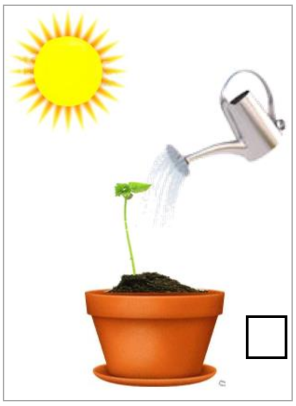

☐

Aufgabe 4

✎ Gib an, <u>was</u> in dem Experiment gemessen wird. *(P_Pfl_B_O)*

Erläutere in 1-2 Sätzen, <u>wie</u> gemessen werden kann.

Versuche mit Asseln

Um seine Vermutung bezüglich des Lebensraums der Asseln zu überprüfen, befüllt Jonas ein Gefäß mit Sand. Eine Hälfte befeuchtet er mit etwas Wasser. In die Mitte des Gefäßes setzt er mehrere Asseln und deckt das Gefäß abschließend mit einer dunklen Folie ab.

Jonas Versuchsansatz

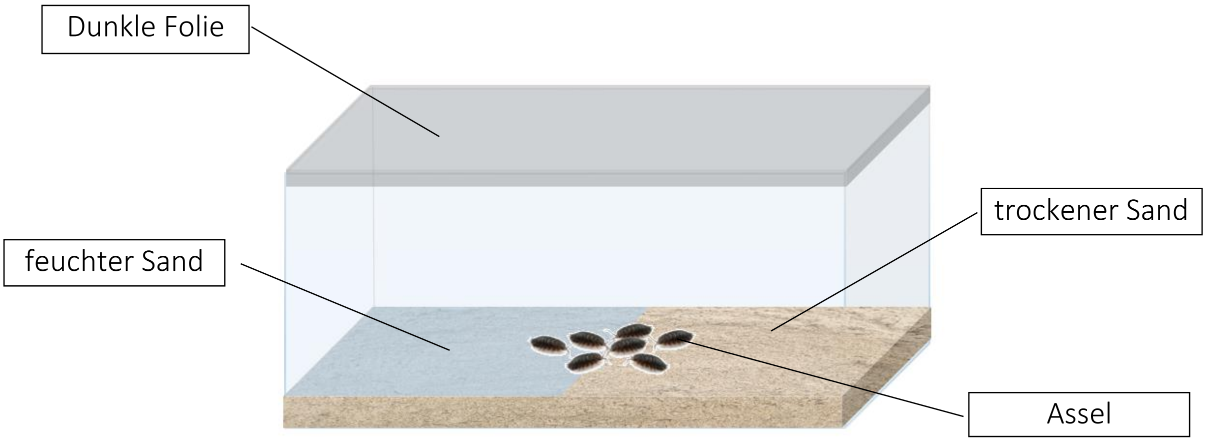

Aufgabe 5

Welche Vermutung möchte Jonas überprüfen? *(P_Ass_F_MC)*

✎ Kreuze an!

☐ Die Folie erzeugt unterschiedliche Lichtverhältnisse im Gefäß.

☐ Asseln bevorzugen dunkle Lebensräume.

☐ Asseln bevorzugen feuchte Lebensräume.

☐ Die Temperatur in den beiden Gefäßhälften unterscheidet sich.

Aufgabe 6

✎ **Erkläre in 1-2 Sätzen, warum die Asseln genau in der Mitte des Gefäßes platziert werden müssen.**

(P_Ass_F_O)

Versuche mit Rückenschwimmern

Rückenschwimmer sind im Wasser lebende Insekten, die unter anderem Larven anderer Insekten fangen und aussaugen. Laura macht ein Experiment mit Rückenschwimmern in einem Wasserbecken. In einem ersten Versuch präsentiert sie mehrmals (fünfmal) einem Rückenschwimmer gut sichtbar die Attrappe(Nachbildung) einer Insektenlarve. Dabei achtet sie darauf, dass sie stets zuerst die Attrappe und dann erst den Rückenschwimmer ins Wasserbecken setzt. Sie notiert ihre Beobachtungen.

In einem anderen Versuch erzeugt sie durch ein Stück Draht Bewegungen im Wasser. Auch diesen Vorgang wiederholt sie fünfmal und hält ihre Beobachtungen fest.

Sie kommt zu folgenden Ergebnissen:

Versuch	Gegenstand im Wasserbecken	Vorgang	Beobachtetes Verhalten des Rückenschwimmers
1	Attrappe eines Beutetiers	Attrappe wird der Wasserwanze gut sichtbar präsentiert	Rückenschwimmer zeigt bei allen fünf Durchführungen keine Reaktion
2	Draht	Draht ruft Erschütterungen im Wasser hervor	Rückenschwimmer schwimmt bei vier von fünf Durchführungen blitzartig zur Erschütterungsstelle

Aufgabe 7

Welche Frage will Laura mit ihrem Experiment klären? ✏ Kreuze an! *(P_Rü_1_MC)*

☐ Spielt die Dicke des Drahts eine Rolle bei der Reaktion des Rückenschwimmers?

☐ Bemerkt der Rückenschwimmer seine Beute durch das Sehen oder durch die Wahrnehmung der Wasserbewegung?

☐ Erkennt der Rückenschwimmer den Unterschied zwischen einer Attrappe und einem echten Beutetier?

☐ Spielt die Größe der Attrappe eine Rolle bei der Reaktion des Rückenschwimmers?

Aufgabe 8

✏ Erkläre in 1-2 Sätzen, warum Laura die Vorgänge mehrmals durchführt statt jeweils nur einmal.

(P_Rü_1_O)

BEANTWORTE DIE NÄCHSTEN 6 AUFGABEN NUR, WENN DU DAS LERNPROGRAMM VON PROF. PLANKTON IN DER SCHULE BEARBEITET HAST!

(1) Was fressen Großlibellenlarven neben Mückenlarven noch? *(P_Vor1)*
✎ Kreuze an!

Libellenlarven fressen ...
- ☐ Schilfpflanzen.
- ☐ Grünalgen.
- ☐ Wasserflöhe.
- ☐ Mücken.

(2) **Wodurch wird der Beutefangreflex der Libellenlarve ausgelöst?** *(P_Vor2)*
✎ Kreuze an!

Der Beutefangreflex der Libellenlarve wird ausgelöst durch...
- ☐ Duftstoffe der Beute.
- ☐ Laute der Beute.
- ☐ die Farbe der Beute.
- ☐ alle drei Faktoren (Duftstoffe, Laute, Farbe).

(3) **Welche Beutetiere fangen Libellenlarven bevorzugt?** *(P_Vor3)*
✎ Kreuze an!

Libellenlarven fressen ...
- ☐ überwiegend dunkle/farbige Mückenlarven.
- ☐ überwiegend helle/farblose Mückenlarven.
- ☐ farbige und farblose Mückenlarven zu gleichermaßen.
- ☐ Mückenlarven mit bestimmtem Farbmuster.

(4) Die Farbe der Beute hat einen Einfluss auf den Beutefangreflex der Großlibellenlarve. Wie wurde das im Experiment mit der Großlibellenlarve kontrolliert/sichergestellt? *(P_CVS_K)*
✎ Kreuze an!

Dies wurde kontrolliert, indem...
- ☐ man die Libellenlarven zwischen einem dunklen/farbigen und einem hellen/ farblosen Beutetier wählen ließ.
- ☐ man mehrere Libellenlarven im Experiment einsetzte.
- ☐ man die Libellenlarven vorher fütterte.
- ☐ man den Libellenlarven eine Eingewöhnungszeit im Wasserbecken gab.

(5) Warum wurden mehrere Versuchstiere im Experiment eingesetzt? *(P_CVS_W)*

✐ Kreuze an!

Es wurden mehrere Versuchstiere eingesetzt,...

- ☐ um ausschließen, dass die Versuchsergebnisse zufällig zustande kommen.
- ☐ da sich Großlibellenlarven nur in Gruppen wohl fühlen und in der Natur nicht anders anzutreffen sind.
- ☐ da Großlibellenlarven sich allein ganz anders verhalten als in Gruppen.
- ☐ um zu kontrollieren, dass die Farbe den Beutefangreflex auslöst.

(6) Welche Störgrößen können die Ergebnisse des Experiments verfälscht haben? *(P_CVS_S)*

✐ Kreuze an!

Die Ergebnisse können durch folgende Störgrößen beeinflusst worden sein: Durch ...

- ☐ die Verwendung von zwei Attrappen, die sich in einer Eigenschaft (Farbe) unterscheiden.
- ☐ den Einsatz mehrerer Libellenlarven.
- ☐ die Fütterung einiger Versuchstiere.
- ☐ eine Eingewöhnungszeit im Wasserbecken für alle Versuchstiere.

NUN HAST DU ES FAST GESCHAFFT!

ÜBERPRÜFE NOCH EINMAL, OB DU ALLE 4 BZW. 6 SEITEN BEARBEITET HAST!

VIELEN DANK FÜR DEINE MITARBEIT!
