# Supplementary material for: Self-Generation in the Context of Inquiry-Based Learning
Source: Front Psychol. 2018 Dec 13;9:2440. doi: 10.3389/fpsyg.2018.02440 (PMC6315139; doi:10.3389/fpsyg.2018.02440)
Supplement: FIGURE S5 — Posttest_1. [file Image_5.pdf]

Liebe Schülerinnen und Schüler,

Im Folgenden erhaltet ihr Aufgaben, um zu überprüfen, wie aufmerksam ihr im Lernprogramm zugehört habt und wie gut ihr euch mit wissenschaftlichen Experimenten bereits auskennt. Versucht die Aufgaben so gut es geht zu lösen. Es ist sehr wichtig, dass ihr euch beim Bearbeiten der Aufgaben große Mühe gebt. Bitte beantwortet jede Aufgabe selbständig und so gut ihr könnt.

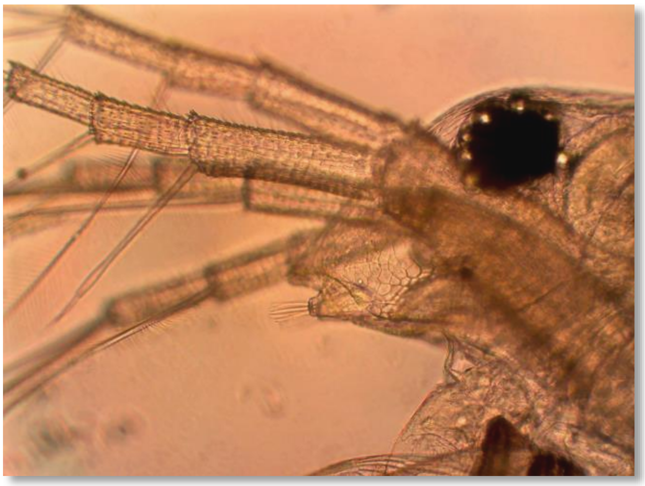

06	12	06			

(*hier müsst ihr nichts eintragen*)

Hier findest du kurze Aufgaben zum Experimentieren. Einige der Aufgaben werden dir sicherlich leicht fallen, andere sind etwas schwerer.
Falls du dir bei einer Aufgabe unsicher sein solltest, versuche trotzdem eine Antwort zu geben.

Bitte bearbeitet die Aufgaben so, wie es in den folgenden Beispielen gezeigt wird.

Bei Aufgaben wie in BEISPIEL 1 sollst du immer NUR EIN Kreuz setzen.

BEISPIEL 1

Wasserflöhe besitzen ....
✎ Kreuze an!

- ☐ ein Antennenpaar
- ☒ zwei Antennenpaare
- ☐ drei Antennenpaare
- ☐ vier Antennenpaare

Falls du es dir anders überlegt hast und deine Antwort ausbessern möchtest, dann mache dies folgendermaßen:

Eine Vermutung mit einer passenden Begründung nennt man...
✎ Kreuze an!

- ☐ ein Antennenpaar
- ■ zwei Antennenpaare
- ☒ drei Antennenpaare
- ☐ vier Antennenpaare

Bei einigen Aufgaben in diesem Heft musst du kurze Antwortsätze formulieren. Diese Aufgaben sehen aus wie **BEISPIEL 2**:

BEISPIEL 2

Warum gehören Wasserflöhe zu den Krebstieren und nicht zu den Insekten (Flöhe)?

Erkläre in 2-3 Sätzen

Insekten besitzen nur 6 Beine, Wasserflöhe haben aber 10.
Sie besitzen keine harte Schale.

Nun kannst du umblättern und mit der Bearbeitung der Aufgaben beginnen

Versuche mit Wasserflöhen

Wenn die Sonne auf die Oberfläche eines Sees scheint, tauchen Wasserflöhe in tiefere Schichten ab. Maren vermutet, dass Wasserflöhe Licht meiden. Um ihre Vermutung zu überprüfen, gibt Maren nach der Fütterung der Wasserflöhe einige in ein Wasserbecken. Das Becken platziert sie auf einem ebenen Untergrund. Im Anschluss dunkelt sie einen Bereich des Beckens mit schwarzer Pappe ab. Die andere Seite des Gefäßes beleuchtet sie mit einer LED-Lampe, die Licht aussendet, jedoch nicht das Wasser erwärmt.

Marens Versuchsansatz

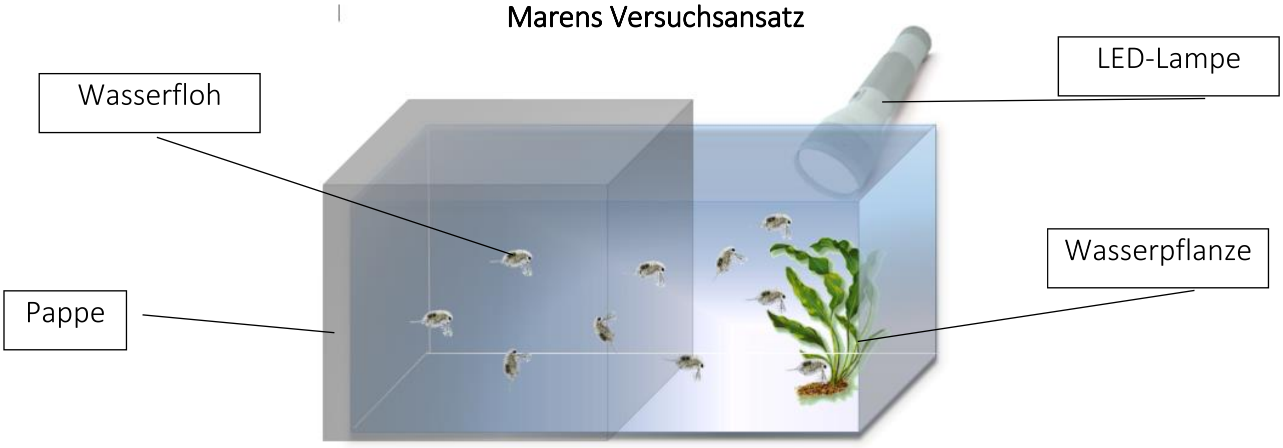

Aufgabe 1

Warum ist das geplante Experiment nicht das Richtige, um Marens Vermutung zu überprüfen?

✎ Kreuze an! *(E1_Was_P_MC)*

☐ Die Wasserflöhe dürfen nicht gefüttert werden.

☐ Die Wasserflöhe dürfen nicht beleuchtet werden.

☐ Die Wasserpflanze kann die Wasserflöhe in ihrer Reaktion beeinflussen.

☐ Das LED-Licht kann die Reaktion der Wasserflöhe beeinflussen.

Aufgabe 2

✎ **Erkläre in 1-2 Sätzen, warum eine LED-Lampe statt einer gewöhnlichen Taschenlampe verwendet werden muss.** *(E1_Was_P_O)*

Pflanzenwachstum

Amelie möchte herausfinden, welche Lichtverhältnisse für das Wachstum von Bohnen am besten geeignet sind. Sie vermutet, dass Bohnen im Sonnenlicht besser wachsen als im Schatten. Um ihre Vermutung zu überprüfen, pflanzt mehrere Bohnen in zwei Töpfe mit Gartenerde. Sie stellt einen Topf auf die Fensterbank ins Sonnenlicht und einen Topf an einen schattigen Platz auf ihrem Bücherregal. Amelie weiß, dass Pflanzen Wasser und Mineralstoffe für ihr Wachstum benötigen. Daher gießt und düngt sie die Bohnen wöchentlich.

Amelies Versuchsansätze

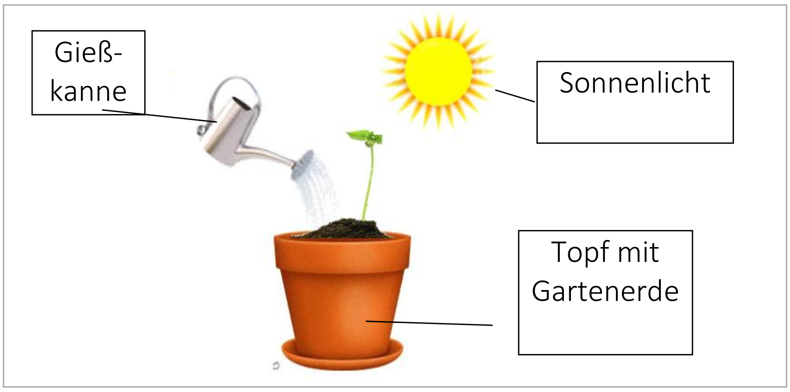

Aufgabe 3

Welchen zweiten Versuchsansatz benötigt Amelie? Kreuze an! *(E1_Pfl_L_MC)*

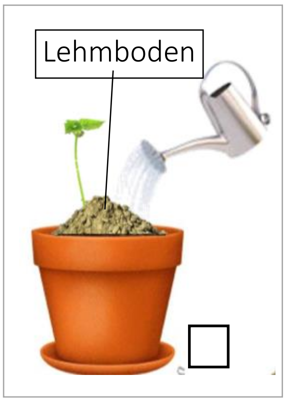

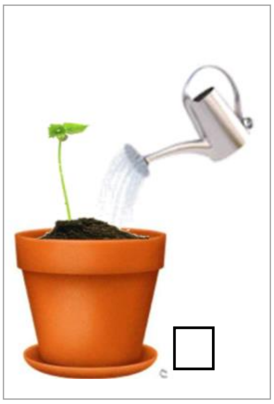

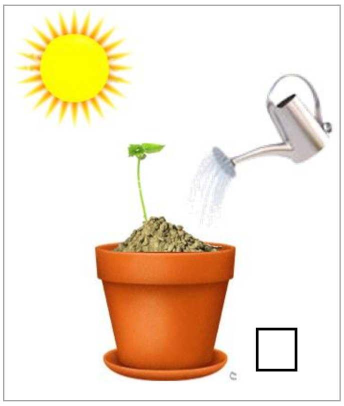

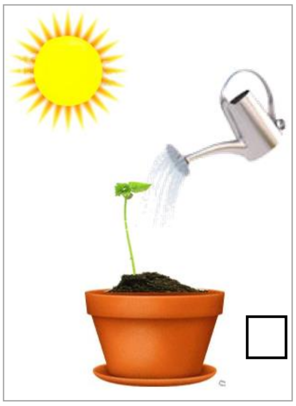

Aufgabe 4

Gib an, <u>was</u> in dem Experiment getestet wird (Testgröße). *(E1_Pfl_L_O1)*

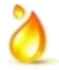 :

Gib an, <u>was</u> in dem Experiment gemessen wird (Messgröße). *(E1_Pfl_L_O2)*

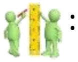 :

Versuche mit Asseln

Um seine Vermutung bezüglich des Lebensraums der Asseln zu überprüfen, befüllt Jonas ein Gefäß mit Sand. In die Mitte des Gefäßes setzt er mehrere Asseln und deckt abschließend die Hälfte des Gefäßes mit einer dunklen Folie ab.

Jonas Versuchsansatz

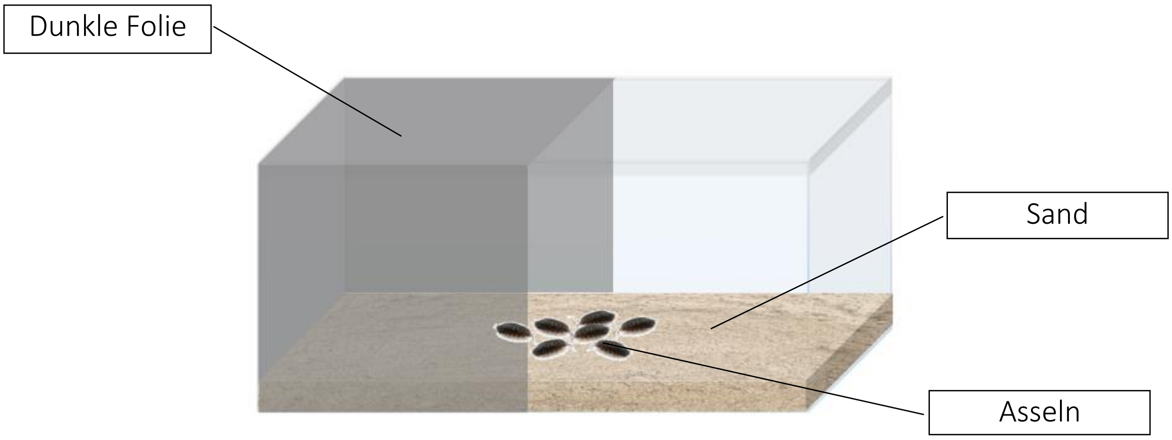

___________________________________Aufgabe 5___________________________________

Welche Vermutung möchte Jonas überprüfen? *(E1_Ass_L_MC)*

✎ Kreuze an!

☐ Asseln bevorzugen feuchte Lebensräume

☐ Asseln bevorzugen dunkle Lebensräume.

☐ Asseln bevorzugen trockene Lebensräume

☐ Asseln bevorzugen kühle Lebensräume.

___________________________________Aufgabe 6___________________________________

✎ Erkläre in 1-2 Sätzen, warum mehrere Asseln im Experiment eingesetzt werden müssen. *(E1_Ass_L_O)*

Süße Zuckerrüben

Der Forscher Andreas Sigimund Marggraf (1709-1782) versuchte besonders zuckerhaltige Zuckerrüben zu züchten, indem er verschiedene Bedingungen beim Anbau veränderte. In einer Versuchsreihe verglich er die folgenden Ansätze:

Marggrafs Versuchsansätze

	Ansatz 1	Ansatz 2	Ansatz 3
Bodentyp	Schwarzerde	Schwarzerde	Schwarzerde
Durchschnittliche Tagestemperatur	20°C	17°C	20°C
Wasser	Tägliche Bewässerung	Tägliche Bewässerung	Tägliche Bewässerung
Pflege	Intensiv	Intensiv	Intensiv
Sonneneinstrahlung	hoch	hoch	mäßig

Aufgabe 7

Welche Fragestellung hat Marggraf mit dem Vergleich von Ansatz 1 und Ansatz 2 untersucht?

Kreuze an! *(E1_Zu_1 _MC)*

☐ Hat die Wassermenge einen Einfluss auf die Zuckerproduktion?

☐ Hat der Bodentyp einen Einfluss auf die Zuckerproduktion von Zuckerrüben?

☐ Hat die Pflege einen Einfluss auf die Zuckerproduktion von Zuckerrüben?

☐ Hat die Temperatur einen Einfluss auf die Zuckerproduktion von Zuckerrüben?

Aufgabe 8

Erkläre, welche Fragestellung mit Ansatz 1 und 3 untersucht werden kann. *(E1_Zu_1 _O)*

Versuche mit Fischen

Lea führt ein Experiment zur Atmung von Fischen durch. Dazu füllt sie drei Wasserbecken mit je 5l Wasser und je einer Wasserpflanze. In Wasserbecken 1 und 3 setzt sie jeweils einen Goldfisch, in Wasserbecken 2 setzt sie drei Goldfische. In Wasserbecken 1 und 2 beträgt die Wassertemperatur 20°C, in Wasserbecken 3 beträgt sie 10°C. Nach einiger Zeit beobachtet Lea, wie häufig die Fische pro Minute atmen. Das erkennt sie daran, wie schnell sich die Kiemendeckel der Fische bewegen.

Leas Versuchsansätze

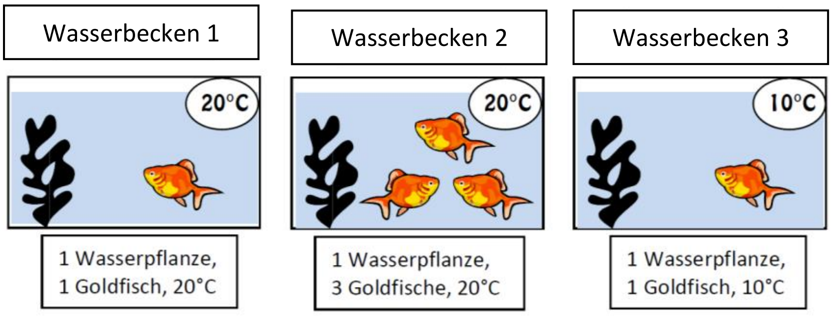

Aufgabe 9

Welche Fragstellung kann Lea mit dem Vergleich von Wasserbecken 1 und 2 untersuchen?

✎ Kreuze an! *(E1_Fi_1 _MC)*

☐ Hat die Wassermenge einen Einfluss auf die Atmung?

☐ Bei welcher Temperatur atmen Fische besonders schnell?

☐ Bei welcher Wassermenge atmen Fische besonders schnell?

☐ Hat die Anzahl der Fische einen Einfluss auf die Atmung?

Aufgabe 10

✎ **Erkläre, welche Fragestellung mit Ansatz 1 und 3 untersucht werden kann.** *(E1_Fi_1 _O)*

(11) Welche Aussage trifft auf Grünalgen zu? *(E1_Alg)*

✏ Kreuze an!

Grünalgen sind die Nahrung der Wasserflöhe und wachsen ...

- ☐ in der oberen Wasserschicht .
- ☐ in der unteren Wasserschicht.
- ☐ in allen Wasserschichten.
- ☐ am Grund des Teichs.

(12) In welcher Wasserschicht jagen die Fressfeinde der Wasserflöhe? *(E1_Fre)*

✏ Kreuze an!

Die Fressfeinde der Wasserflöhe jagen in...

- ☐ allen Wasserschichten.
- ☐ der oberen Wasserschicht .
- ☐ der unteren Wasserschicht.
- ☐ nur am Ufer.

(13) Welche Aussage trifft auf die ultraviolette Strahlung in einem See zu? *(E1_UV)*

✏ Kreuze an!

Die ultraviolette Strahlung ist in der...

- ☐ oberen Wasserschicht am höchsten.
- ☐ unteren Wasserschicht am höchsten.
- ☐ mittleren Wasserschicht am höchsten.
- ☐ in allen Schichten gleich hoch.

---

(14) Was fressen Großlibellenlarven neben Mückenlarven noch? *(E1_Vor1)*

✏ Kreuze an!

Libellenlarven fressen ...

- ☐ Wasserflöhe.
- ☐ Mücken.
- ☐ Schilfpflanzen.
- ☐ Grünalgen.

(15) Die Farbe der Beute hat einen Einfluss auf den Beutefangreflex der Großlibellenlarve. Wie wurde das im Experiment mit der Großlibellenlarve kontrolliert/sichergestellt? *(E1_CVS_K)*

✏ Kreuze an!

Dies wurde kontrolliert, indem...

- ☐ man mehrere Libellenlarven im Experiment einsetzte.
- ☐ man den Libellenlarven eine Eingewöhnungszeit im Wasserbecken gab.
- ☐ man die Libellenlarven vorher fütterte.
- ☐ man die Libellenlarven zwischen einem dunklen/farbigen und einem hellen/ farblosen Beutetier wählen ließ.

Zum Schluss….

noch drei kurze Fragen, um einzuschätzen, wie schwierig du die Aufgaben in diesem Testheft findest. Bitte versuche, dich so genau wie möglich einzuschätzen. Mache immer nur ein Kreuz pro Frage.

1. Wie schwierig war es für dich die Aufgaben zu verstehen?

☐	☐	☐	☐	☐	☐
1<br>einfach	2	3	4	5	6<br>sehr schwierig

2. Wie schwierig war es für dich mit diesem Testheft zu arbeiten?

☐	☐	☐	☐	☐	☐
1<br>einfach	2	3	4	5	6<br>sehr schwierig

3. Wie sehr hast du dich bei der Bearbeitung der Aufgaben angestrengt?

☐	☐	☐	☐	☐	☐
1<br>Wenig	2	3	4	5	6<br>sehr

NUN HAST DU ES FAST GESCHAFFT!
ÜBERPRÜFE NOCH EINMAL, OB DU ALLE 7 SEITEN BEARBEITET HAST! VIELEN DANK FÜR DEINE MITARBEIT!
