# Supplementary material for: Self-Generation in the Context of Inquiry-Based Learning
Source: Front Psychol. 2018 Dec 13;9:2440. doi: 10.3389/fpsyg.2018.02440 (PMC6315139; doi:10.3389/fpsyg.2018.02440)
Supplement: FIGURE S6 — Posttest_2. [file Image_6.pdf]

Liebe Schülerinnen und Schüler,

Im Folgenden erhaltet ihr Aufgaben, um zu überprüfen, wie gut ihr euch mit wissenschaftlichen Experimenten auskennt. Versucht die Aufgaben so gut es geht zu lösen. Es ist sehr wichtig, dass ihr euch beim Bearbeiten der Aufgaben große Mühe gebt. Bitte beantwortet jede Aufgabe selbständig und so gut ihr könnt.

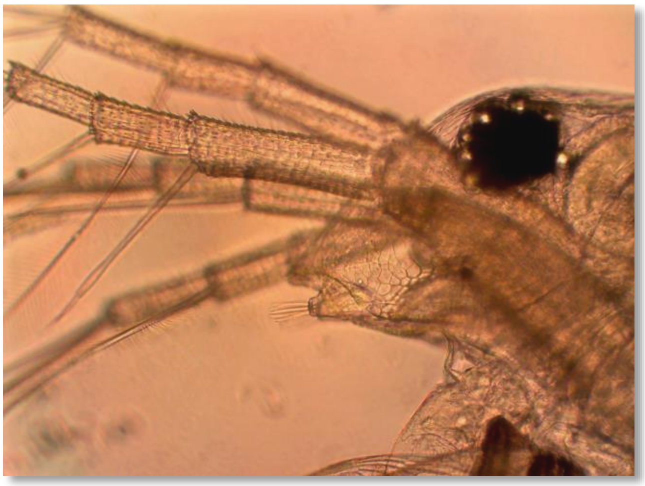

**06**	12	06			

(*hier müsst ihr nichts eintragen*)

Hier findest du kurze Aufgaben zum Experimentieren. Einige der Aufgaben werden dir sicherlich leicht fallen, andere sind etwas schwerer.
Falls du dir bei einer Aufgabe unsicher sein solltest, versuche trotzdem eine Antwort zu geben.

Bitte bearbeitet die Aufgaben so, wie es in den folgenden Beispielen gezeigt wird.

Bei Aufgaben wie in BEISPIEL 1 sollst du immer **NUR EIN** Kreuz setzen.

BEISPIEL 1

Wasserflöhe besitzen ….
✎ Kreuze an!

☐ ein Antennenpaar
☒ zwei Antennenpaare
☐ drei Antennenpaare
☐ vier Antennenpaare

Falls du es dir anders überlegt hast und deine Antwort ausbessern möchtest, dann mache dies folgendermaßen:

Eine Vermutung mit einer passenden Begründung nennt man...
✎ Kreuze an!

☐ ein Antennenpaar
■ zwei Antennenpaare
☒ drei Antennenpaare
☐ vier Antennenpaare

Bei einigen Aufgaben in diesem Heft musst du kurze Antwortsätze formulieren. Diese Aufgaben sehen aus wie **BEISPIEL 2**:

BEISPIEL 2

Warum gehören Wasserflöhe zu den Krebstieren und nicht zu den Insekten (Flöhe)?

Erkläre in 2-3 Sätzen

Insekten besitzen nur 6 Beine, Wasserflöhe haben aber 10.
Sie besitzen keine harte Schale.

Nun kannst du umblättern und mit der Bearbeitung der Aufgaben beginnen

Versuche mit Wasserflöhen

Wenn die Sonne auf die Oberfläche eines Sees scheint, tauchen Wasserflöhe in tiefere Schichten ab. Maren vermutet, dass Wasserflöhe Licht meiden. Um ihre Vermutung zu überprüfen, gibt Maren nach der Fütterung einiger Wasserflöhe zehn in ein Wasserbecken. Das Becken platziert sie auf einem ebenen Untergrund. Im Anschluss dunkelt sie einen Bereich des Beckens mit schwarzer Pappe ab. Die andere Seite des Gefäßes beleuchtet sie mit einer LED-Lampe, die Licht aussendet, jedoch nicht das Wasser erwärmt.

Marens Versuchsansatz

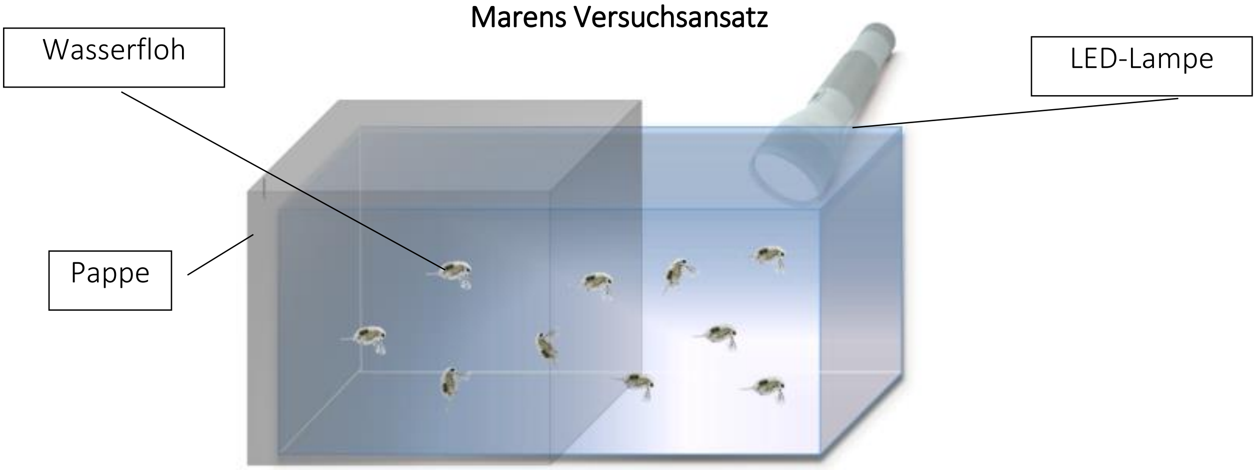

Aufgabe 1

Warum ist das geplante Experiment nicht das Richtige, um Marens Vermutung zu überprüfen?

✎ Kreuze an! *(E2_Was_F_MC)*

☐ Die Wasserflöhe dürfen alle nicht gefüttert werden.

☐ Die Größe des hellen Bereichs kann die Wasserflöhe in ihrer Reaktion beeinflussen.

☐ Die Wasserflöhe dürfen alle nicht beleuchtet werden.

☐ Das LED-Licht kann die Reaktion der Wasserflöhe beeinflussen.

Aufgabe 2

✎ **Erkläre in 1-2 Sätzen, warum genau die Hälfte des Gefäßes mit dunkler Pappe abgedeckt wird.**

(E2_Was_F_O)

Pflanzenwachstum

Amelie möchte herausfinden, wieviel Wasser für das Wachstum von Bohnen am besten geeignet ist. Sie vermutet, dass Bohnen besser wachsen, wenn sie häufig gegossen werden. Um ihre Vermutung zu überprüfen, nimmt sie zwei Töpfe und pflanzt in beide Töpfe mehrere Bohnen. Sie stellt sie in ihrem Zimmer auf die Fensterbank über die Heizung. Amelie weiß, dass Pflanzen Mineralstoffe für ihr Wachstum benötigen. Daher düngt sie die Bohnen wöchentlich.

Amelies Versuchsansätze

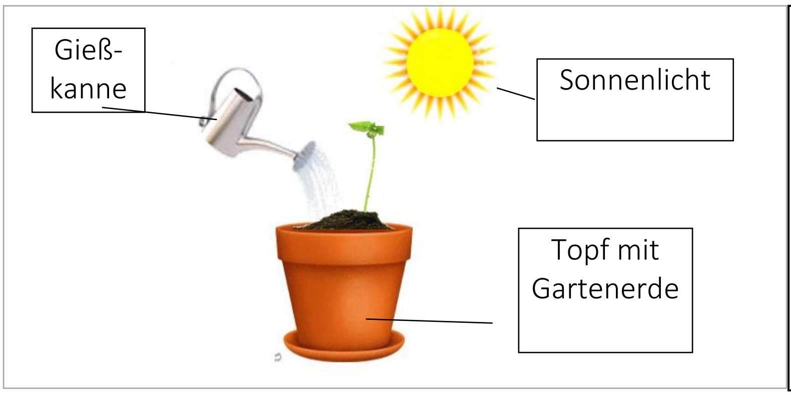

Gießen: täglich
Düngen: 1-mal in der Woche

Aufgabe 3

Welchen Kontrollansatz benötigt Amelie? ✎ Kreuze an! *(E2_Pfl_W_MC)*

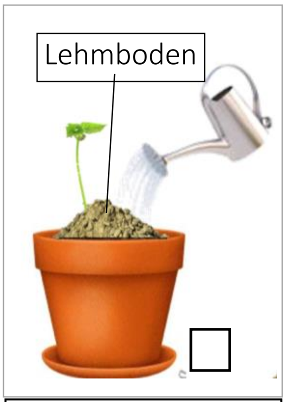

☐

Gießen: täglich
Düngen: 1-mal in der Woche

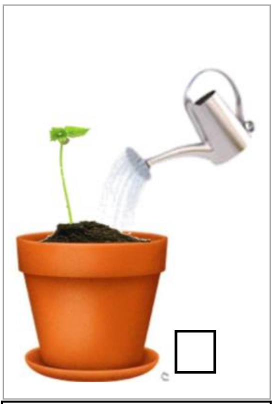

☐

Gießen: täglich
Düngen: 1-mal in der Woche

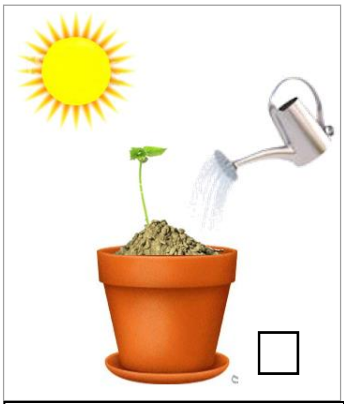

☐

Düngen und Gießen:
1- mal in der Woche

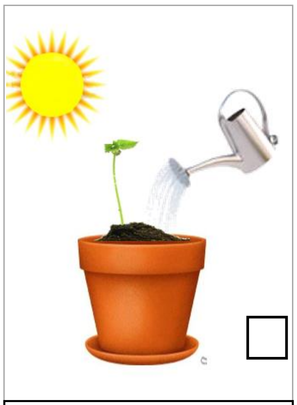

☐

Gießen und Düngen: 1-mal in der Woche

Aufgabe 4

✎ Gib an, <u>was</u> in dem Experiment gemessen wird (Messgröße). *(E2_Pfl_W_O)*

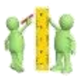 :

Versuche mit Asseln

Um seine Vermutung bezüglich des Lebensraums der Asseln zu überprüfen, befüllt Jonas ein Gefäß mit Sand. Er befestigt auf der rechten Seite einen Heizstab. In die Mitte setzt er mehrere Asseln und deckt abschließend das Gefäß mit einer dunklen Folie ab.

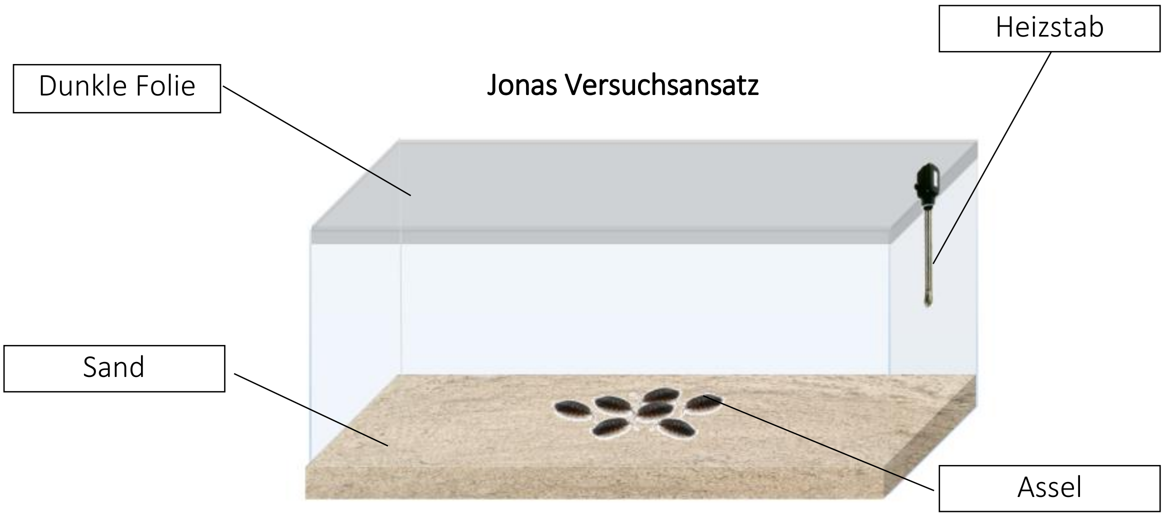

Aufgabe 5

Welche Vermutung möchte Jonas überprüfen? *(E2_Ass_W_MC)*

✎ Kreuze an!

☐ Asseln bevorzugen trockene Lebensräume.

☐ Asseln bevorzugen kalte Lebensräume.

☐ Asseln bevorzugen feuchte Lebensräume

☐ Asseln bevorzugen dunkle Lebensräume.

Aufgabe 6

✎ **Erkläre in 1-2 Sätzen, warum die Asseln gleichzeitig im Gefäß platziert werden müssen.**

(E2_Ass_W_O)

Süße Zuckerrüben

Der Forscher Andreas Sigimund Marggraf (1709-1782) versuchte besonders zuckerhaltige Zuckerrüben zu züchten, indem er verschiedene Bedingungen beim Anbau veränderte. In einer Versuchsreihe verglich er die folgenden Ansätze:

Marggrafs Versuchsansätze

	Ansatz 1	Ansatz 2	Ansatz 3
Bodentyp	Schwarzerde	Schwarzerde	Schwarzerde
Durchschnittliche Tagestemperatur	20°C	20°C	20°C
Wasser	Wöchentliche Bewässerung	Wöchentliche Bewässerung	Tägliche Bewässerung
Pflege	Gering	Intensiv	Intensiv
Sonneneinstrahlung	hoch	hoch	hoch

Aufgabe 7

Welche Fragestellung hat Marggraf mit dem Vergleich von Ansatz 1 und Ansatz 2 untersucht?

✎ Kreuze an! *(E2_Zu_2 _MC)*

☐ Hat die Wassermenge einen Einfluss auf die Zuckerproduktion von Zuckerrüben?

☐ Hat der Bodentyp einen Einfluss auf die Zuckerproduktion von Zuckerrüben?

☐ Hat die Pflege einen Einfluss auf die Zuckerproduktion von Zuckerrüben?

☐ Hat die Temperatur einen Einfluss auf die Zuckerproduktion von Zuckerrüben?

Aufgabe 8

✎ **Erkläre, welche Fragestellung mit Ansatz 2 und 3 untersucht werden kann.**

(E2_Zu_2 _O)

Versuche mit Fischen

Lea führt ein Experiment zur Atmung von Fischen durch. Dazu füllt sie drei Wasserbecken mit je 5l Wasser und je einer Wasserpflanze. In jedes Wasserbecken setzt sie jeweils drei Goldfische. In Wasserbecken 1 beträgt die Wassertemperatur 20°C, in Wasserbecken 2 und 3 beträgt sie 10°C. Nach einiger Zeit beobachtet Lea, wie häufig die Fische pro Minute atmen. Das erkennt sie daran, wie schnell sich die Kiemendeckel der Fische bewegen.

Leas Versuchsansätze

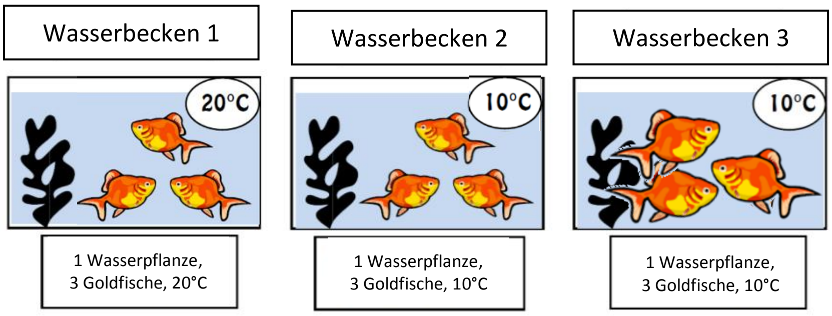

Aufgabe 9

Welche Fragstellung kann Lea mit dem Vergleich von Wasserbecken 1 und 2 untersuchen?

✎ Kreuze an! *(E2_Fi_2 _MC)*

☐ Hat die Wassermenge einen Einfluss auf die Atmung?

☐ Hat die Wassertemperatur einen Einfluss auf die Atmung?

☐ Hat die Körpergröße der Fische einen Einfluss auf die Atmung?

☐ Hat die Anzahl der Fische einen Einfluss auf die Atmung?

Aufgabe 10

✎ **Erkläre, welche Fragestellung mit Ansatz 2 und 3 untersuchen werden kann.** *(E2_Fi_2 _O)*

Brausetabletten

Brausetabletten sprudeln, wenn man sie ins Wasser gibt, da Kohlenstoffdioxid freigesetzt wird. Tim möchte herausfinden, ob die Abgabe von Kohlenstoffdioxid in Wasser temperaturabhängig ist. Er vermutet, dass die Temperatur keinen Einfluss auf die Abgabe von Kohlenstoffdioxid hat. Er nimmt zwei Gefäße. Eines befüllt er mit 20°C warmem Leitungswasser; das andere mit Mineralwasser, das er im Kühlschrank auf 0°C gekühlt hat. Er gibt zur selben Zeit jeweils eine Brausetablette in beide Gefäße und beobachtet die Gasentwicklung. Für beide Gefäße misst er die Zeit, die es braucht, bis sich die Brausetabletten aufgelöst haben.

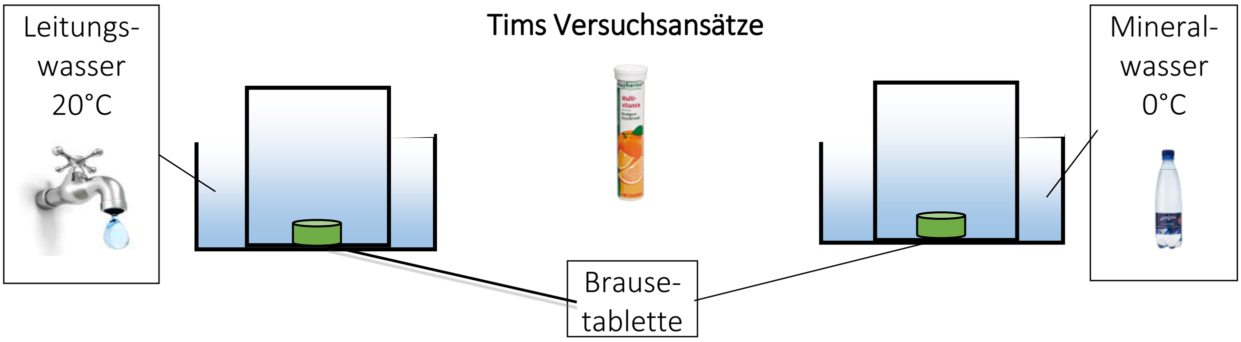

Aufgabe 11

Warum ist das geplante Experiment nicht das Richtige, um Tims Vermutung zu überprüfen?

Kreuze an! *(E2_B_1_MC)*

☐ Die Zugabe der Tabletten in die Gefäße erfolgt nicht gleichzeitig.

☐ Die Art des Wassers hat einen Einfluss auf das Ergebnis.

☐ Die Gefäßformen sind ungeeignet, um Kohlenstoffdioxid aufzufangen.

☐ Die Temperatur in den Gefäßen wurde nicht konstant gehalten.

Aufgabe 12

Gib an, <u>was</u> in Tims Experiment getestet werden soll (Testgröße). *(E2_B_1_O)*

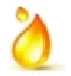 :

(13) Welche Aussage trifft auf die ultraviolette Strahlung in einem See zu? *(E2_UV)*

✎ Kreuze an!

Die ultraviolette Strahlung ist in ...

- ☐ der oberen Wasserschicht am höchsten.
- ☐ der unteren Wasserschicht am höchsten.
- ☐ der mittleren Wasserschicht am höchsten.
- ☐ allen Schichten gleich hoch.

(14) Welche Aussage trifft auf Grünalgen zu? *(E2_Alg)*

✎ Kreuze an!

Grünalgen sind die Nahrung der Wasserflöhe und wachsen ...

- ☐ in allen Wasserschichten.
- ☐ am Grund des Teichs.
- ☐ in der oberen Wasserschicht.
- ☐ in der unteren Wasserschicht.

(15) In welcher Wasserschicht jagen die Fressfeinde der Wasserflöhe? *(E2_Fre)*

✎ Kreuze an!

Die Fressfeinde der Wasserflöhe jagen ...

- ☐ nur am Ufer.
- ☐ in der unteren Wasserschicht.
- ☐ in der oberen Wasserschicht .
- ☐ in allen Wasserschichten.

---

(16) **Wodurch wird der Beutefangreflex der Libellenlarve ausgelöst**? *(E2_Vor2)*

✎ Kreuze an!

Der Beutefangreflex der Libellenlarve wird ausgelöst durch…

- ☐ Duftstoffe der Beute.
- ☐ die Farbe der Beute.
- ☐ alle drei Faktoren (Duftstoffe, Laute, Farbe).
- ☐ Laute der Beute.

(17) Warum wurden mehrere Versuchstiere im Experiment zum Beutefang der Libellenlarve eingesetzt? ✎ Kreuze an! *(E2_CVS_W)*

Es wurden mehrere Versuchstiere eingesetzt,...

- ☐ um zu kontrollieren, dass die Farbe den Beutefangreflex auslöst.
- ☐ da Großlibellenlarven sich allein ganz anders verhalten als in Gruppen.
- ☐ da sich Großlibellenlarven nur in Gruppen wohl fühlen und in der Natur nicht anders anzutreffen sind.
- ☐ um ausschließen, dass die Versuchsergebnisse zufällig zustande kommen.

Zum Schluss….

noch drei kurze Fragen, um einzuschätzen, wie schwierig du die Aufgaben in diesem Testheft findest. Bitte versuche, dich so genau wie möglich einzuschätzen. Mache immer nur ein Kreuz pro Frage.

1. Wie schwierig war es für dich die Aufgaben zu verstehen?

☐	☐	☐	☐	☐	☐
1<br>einfach	2	3	4	5	6<br>sehr schwierig

2. Wie schwierig war es für dich mit diesem Testheft zu arbeiten?

☐	☐	☐	☐	☐	☐
1<br>einfach	2	3	4	5	6<br>sehr schwierig

3. Wie sehr hast du dich bei der Bearbeitung der Aufgaben angestrengt?

☐	☐	☐	☐	☐	☐
1<br>Wenig	2	3	4	5	6<br>sehr

NUN HAST DU ES FAST GESCHAFFT!
ÜBERPRÜFE NOCH EINMAL, OB DU ALLE 8 SEITEN BEARBEITET HAST! VIELEN DANK FÜR DEINE MITARBEIT!
