# Supplementary material for: Self-Generation in the Context of Inquiry-Based Learning
Source: Front Psychol. 2018 Dec 13;9:2440. doi: 10.3389/fpsyg.2018.02440 (PMC6315139; doi:10.3389/fpsyg.2018.02440)
Supplement: FIGURE S7 — Posttest_3. [file Image_7.pdf]

Liebe Schülerinnen und Schüler,

Im Folgenden erhaltet ihr Aufgaben, um zu überprüfen, wie gut ihr euch mit wissenschaftlichen Experimenten auskennt. Versucht die Aufgaben so gut es geht zu lösen. Es ist sehr wichtig, dass ihr euch beim Bearbeiten der Aufgaben große Mühe gebt. Bitte beantwortet jede Aufgabe selbständig und so gut ihr könnt.

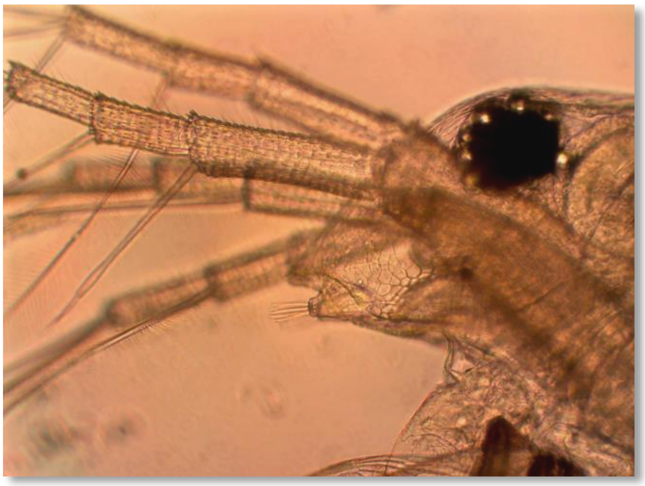

06					

(*hier müsst ihr nichts eintragen*)

Hier findest du kurze Aufgaben zum Experimentieren. Einige der Aufgaben werden dir sicherlich leicht fallen, andere sind etwas schwerer.

Falls du dir bei einer Aufgabe unsicher sein solltest, versuche trotzdem eine Antwort zu geben.

Bitte bearbeitet die Aufgaben so, wie es in den folgenden Beispielen gezeigt wird.

Bei Aufgaben wie in BEISPIEL 1 sollst du immer NUR EIN Kreuz setzen.

BEISPIEL 1

Wasserflöhe besitzen ....

✎ Kreuze an!

- ☐ ein Antennenpaar
- ☒ zwei Antennenpaare
- ☐ drei Antennenpaare
- ☐ vier Antennenpaare

Falls du es dir anders überlegt hast und deine Antwort ausbessern möchtest, dann mache dies folgendermaßen:

Eine Vermutung mit einer passenden Begründung nennt man...

✎ Kreuze an!

- ☐ ein Antennenpaar
- ■ zwei Antennenpaare
- ☒ drei Antennenpaare
- ☐ vier Antennenpaare

Bei einigen Aufgaben in diesem Heft musst du kurze Antwortsätze formulieren. Diese Aufgaben sehen aus wie **BEISPIEL 2**:

BEISPIEL 2

Warum gehören Wasserflöhe zu den Krebstieren und nicht zu den Insekten (Flöhe)?

Erkläre in 2-3 Sätzen

Insekten besitzen nur 6 Beine, Wasserflöhe haben aber 10.
Sie besitzen keine harte Schale.

Nun kannst du umblättern und mit der Bearbeitung der Aufgaben beginnen

Versuche mit Fischen

Lea führt ein Experiment zur Atmung von Fischen durch. Dazu füllt sie drei Wasserbecken mit je 5l Wasser, das 20°C Grad warm ist. In Wasserbecken 1 und 3 setzt sie jeweils einen Goldfisch, in Wasserbecken 2 setzt sie drei Goldfische. Nach einiger Zeit beobachtet Lea, wie häufig die Fische pro Minute atmen. Das erkennt sie daran, wie schnell sich die Kiemendeckel der Fische bewegen.

Leas Versuchsansätze

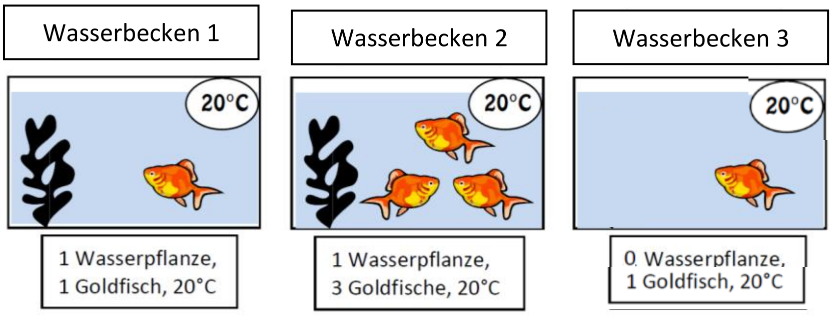

Aufgabe 1

Welche Fragstellung kann Lea mit dem Vergleich von Wasserbecken 1 und 2 untersuchen?

✎ Kreuze an! *(E3_Fi_3 _MC)*

☐ Bei welcher Körpergröße atmen Fische besonders schnell?

☐ Hat die Anzahl der Fische einen Einfluss auf die Atmung?

☐ Hat die Wassermenge einen Einfluss auf die Atmung?

☐ Bei welcher Temperatur atmen Fische besonders schnell?

Aufgabe 2

✎ Erkläre in 1-2 Sätzen, welche Fragestellung mit Ansatz 1 und 3 untersucht werden kann.

(E3_Fi_3 _O)

Versuche mit Rückenschwimmern

Rückenschwimmer sind im Wasser lebende Insekten, die unter anderem Larven anderer Insekten fangen und aussaugen. Laura macht ein Experiment mit Rückenschwimmern in einem Wasserbecken. In einem ersten Versuch präsentiert sie mehrmals (fünfmal) einem Rückenschwimmer gut sichtbar die Attrappe(Nachbildung) einer Insektenlarve. Dabei achtet sie darauf, dass sie stets zuerst die Attrappe und dann erst den Rückenschwimmer ins Wasserbecken setzt. Sie notiert ihre Beobachtungen.

In einem anderen Versuch erzeugt sie durch ein Stück Draht Bewegungen im Wasser. Auch diesen Vorgang wiederholt sie fünfmal und hält ihre Beobachtungen fest.

Sie kommt zu folgenden Ergebnissen:

Versuch	Gegenstand im Wasserbecken	Vorgang	Beobachtetes Verhalten des Rückenschwimmers
1	Attrappe eines Beutetiers	Attrappe wird der Wasserwanze gut sichtbar präsentiert	Rückenschwimmer zeigt bei allen fünf Durchführungen keine Reaktion
2	Draht	Draht ruft Erschütterungen im Wasser hervor	Rückenschwimmer schwimmt bei vier von fünf Durchführungen blitzartig zur Erschütterungsstelle

Aufgabe 3

Welche Frage will Laura mit ihrem Experiment klären?

✎ Kreuze an! *(E3_Rü_2_MC)*

☐ Erkennt der Rückenschwimmer den Unterschied zwischen einer Attrappe und einem echten Beutetier?

☐ Spielt die Größe der Attrappe eine Rolle bei der Reaktion des Rückenschwimmers?

☐ Spielt die Dicke des Drahts eine Rolle bei der Reaktion des Rückenschwimmers?

☐ Bemerkt der Rückenschwimmer seine Beute durch das Sehen oder durch die Wahrnehmung der Wasserbewegung?

Aufgabe 4

✎ **Erkläre in 1-2 Sätzen, warum Laura erst die Attrappe und dann den Rückenschwimmer ins Becken setzt.** *(E3_Rü_2_O)*

Versuche mit Wasserflöhen

Wenn die Sonne auf die Oberfläche eines Sees scheint, tauchen Wasserflöhe in tiefere Schichten ab. Maren vermutet, dass Wasserflöhe Licht meiden. Um ihre Vermutung zu überprüfen, gibt Maren nach der Fütterung der Wasserflöhe zehn in ein Wasserbecken. Das Becken platziert sie auf einem ebenen Untergrund. Im Anschluss dunkelt sie einen Bereich des Beckens mit schwarzer Pappe ab. Die andere Seite des Gefäßes beleuchtet sie mit einer gewöhnlichen Taschenlampe.

Marens Versuchsansatz

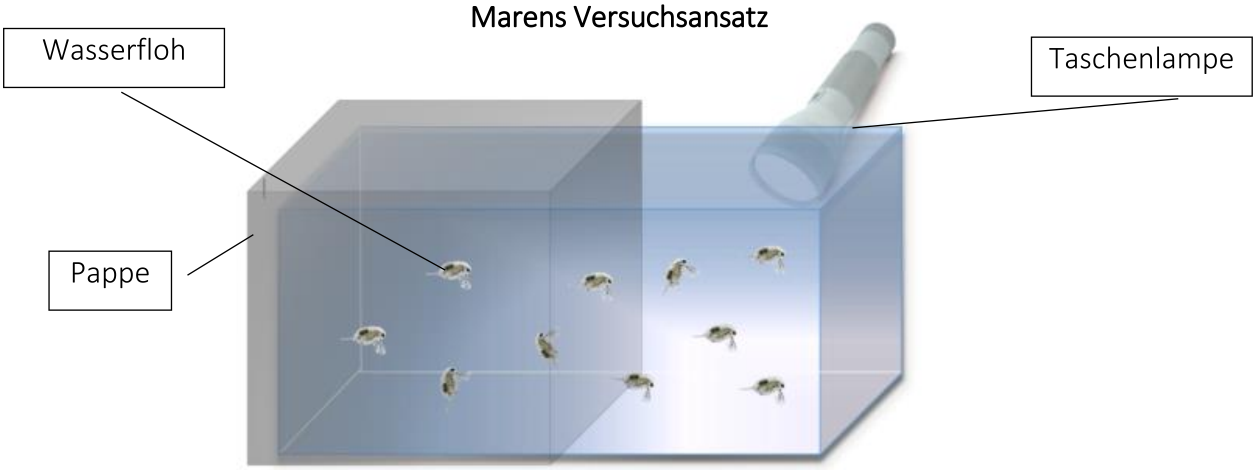

Aufgabe 5

Warum ist das geplante Experiment nicht das Richtige, um Marens Vermutung zu überprüfen?

✎ **Kreuze an!** *(E3_Was_T_MC)*

- ☐ Die Wasserflöhe dürfen alle nicht beleuchtet werden.
- ☐ Die Wasserflöhe dürfen alle nicht gefüttert werden.
- ☐ Die Taschenlampe kann die Reaktion der Wasserflöhe beeinflussen.
- ☐ Die Größe des hellen Bereichs kann die Wasserflöhe in ihrer Reaktion beeinflussen.

Aufgabe 6

✎ **Erkläre in 1-2 Sätzen, warum mehrere Wasserflöhe für das Experiment verwendet werden.**

(E3_Was_T_O)

Brausetabletten

Brausetabletten sprudeln, wenn man sie ins Wasser gibt, da Kohlenstoffdioxid freigesetzt wird. Tim möchte herausfinden, ob die Abgabe von Kohlenstoffdioxid in Wasser durch Schütteln des Gefäßes beeinflusst werden kann. Er vermutet, dass die Wasserbewegung beim Schütteln die Abgabe von Kohlenstoffdioxid beschleunigt. Er nimmt zwei Gefäße und befüllt sie mit Wasser. Er gibt jeweils eine Brausetablette in beide Gefäße und schüttelt eines davon vorsichtig, so dass das Wasser in Bewegung gerät. Er beobachtet die Gasentwicklung. Für beide Gefäße misst er die Zeit, die es braucht, bis sich die Brausetabletten aufgelöst haben.

Tims Versuchsansätze

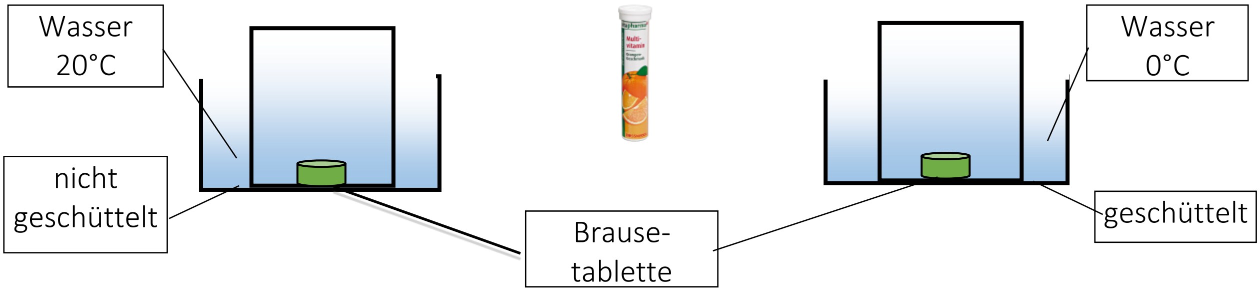

Aufgabe 7

Warum ist das geplante Experiment nicht das Richtige, um Tims Vermutung zu überprüfen?

Kreuze an! *(E3_B_2_MC)*

☐ Das Schütteln hat keinen Einfluss auf das Ergebnis.

☐ Die Gefäßformen sind ungeeignet, um Kohlenstoffdioxid aufzufangen.

☐ Die Temperatur in den Gefäßen wurde nicht konstant gehalten.

☐ Die Bewegung des Wassers hat einen Einfluss auf das Ergebnis.

Aufgabe 8

Gib an, <u>was</u> in Tims Experiment getestet werden soll (Testgröße). *(E3_B_2_O)*

:

Süße Zuckerrüben

Der Forscher Andreas Sigimund Marggraf (1709-1782) versuchte besonders zuckerhaltige Zuckerrüben zu züchten, indem er verschiedene Bedingungen beim Anbau veränderte. In einer Versuchsreihe verglich er die folgenden Ansätze:

Marggrafs Versuchsansätze

	**Ansatz 1**	**Ansatz 2**	**Ansatz 3**
Bodentyp	Schwarzerde	Lehmerde	Lehmerde
Durchschnittliche Tagestemperatur	20°C	20°C	20° C
Wasser	Tägliche Bewässerung	Tägliche Bewässerung	Tägliche Bewässerung
Pflege	Intensiv	Intensiv	Intensiv
Sonneneinstrahlung	hoch	hoch	niedrig

Aufgabe 9

Welche Fragstellung hat Marggraf mit dem Vergleich von Ansatz 1 und Ansatz 2 untersucht?

✎ Kreuze an! *(E3_Zu_3 _MC)*

☐ Hat die Wassermenge einen Einfluss auf die Zuckerproduktion von Zuckerrüben?

☐ Hat die Pflege einen Einfluss auf die Zuckerproduktion von Zuckerrüben?

☐ Hat die Temperatur einen Einfluss auf die Zuckerproduktion von Zuckerrüben?

☐ Hat der Bodentyp einen Einfluss auf die Zuckerproduktion von Zuckerrüben?

Aufgabe 10

✎ **Erkläre in 1-2 Sätzen, warum der Vergleich von Ansatz 1 und 3 nicht sinnvoll ist.**

(E3_Zu_3 _O)

Pflanzenwachstum

Amelie möchte herausfinden, ob Dünger das Wachstum von Bohnen beschleunigt. Sie vermutet, dass Bohnen besser wachsen, wenn sie regelmäßig gedüngt werden. Um ihre Vermutung zu überprüfen, nimmt sie zwei Töpfe und pflanzt in beide Töpfe mehrere Bohnen. Sie stellt sie in ihrem Zimmer auf die Fensterbank über die Heizung. Amelie weiß, dass Pflanzen Wasser für ihr Wachstum benötigen. Daher gießt sie die Bohnen regelmäßig.

Amelies Versuchsansätze

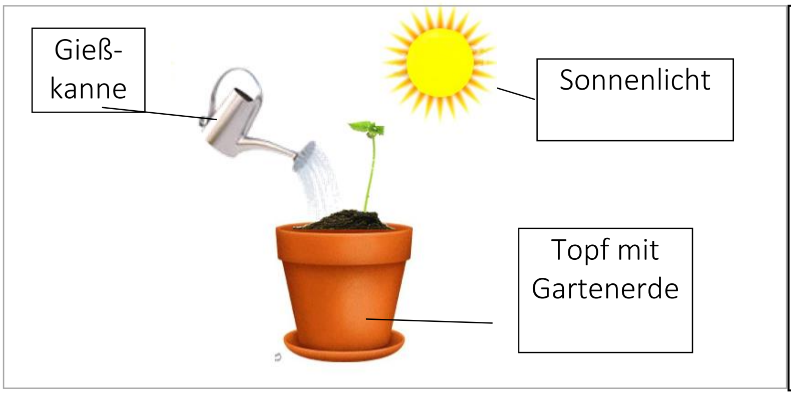

Gießen: täglich
Düngen: 1-mal in der Woche

Aufgabe 11

Welchen Kontrollansatz benötigt Amelie? ✎ Kreuze an! *(E3_Pfl_D_MC)*

- ☐ 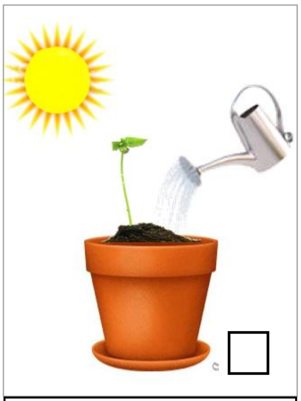
  Gießen: täglich
  Düngen: nie
- ☐ 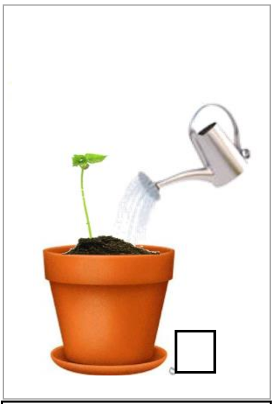
  Gießen: täglich
  Düngen: 1-mal in der Woche
- ☐ 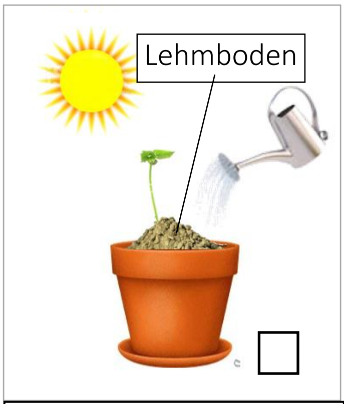

  Gießen: täglich
  Düngen: 1-mal in der Woche
- ☐ 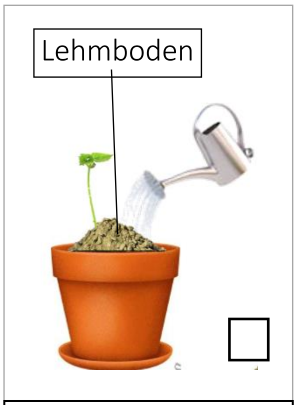

  Gießen: täglich
  Düngen: nie

Aufgabe 12

✎ Gib an, <u>was</u> in dem Experiment gemessen wird (Messgröße). *(E3_Pfl_D_O)*

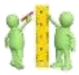 :

(15) Welche Aussage trifft auf die ultraviolette Strahlung in einem See zu? *(E3_UV)*

✏ Kreuze an!

Die ultraviolette Strahlung ist in der...

- ☐ mittleren Wasserschicht am höchsten.
- ☐ unteren Wasserschicht am höchsten.
- ☐ oberen Wasserschicht am höchsten.
- ☐ in allen Schichten gleich hoch.

(16) Welche Aussage trifft auf Grünalgen zu? *(E3_Alg)*

✏ Kreuze an!

Grünalgen sind die Nahrung der Wasserflöhe und wachsen ...

- ☐ in der unteren Wasserschicht.
- ☐ in allen Wasserschichten.
- ☐ am Grund des Teichs.
- ☐ in der oberen Wasserschicht .

(17) In welcher Wasserschicht jagen die Fressfeinde der Wasserflöhe? *(E3_Fre)*

✏ Kreuze an!

Die Fressfeinde der Wasserflöhe jagen...

- ☐ nur am Ufer.
- ☐ in der unteren Wasserschicht.
- ☐ in der oberen Wasserschicht .
- ☐ in allen Wasserschichten.

---

(18) Welche Beutetiere fangen Libellenlarven bevorzugt? *(E3_Vor3)*

✏ Kreuze an!

Libellenlarven fressen ...

- ☐ überwiegend dunkle/farbige Mückenlarven.
- ☐ überwiegend helle/farblose Mückenlarven.
- ☐ farbige und farblose Mückenlarven zu gleichermaßen.
- ☐ Mückenlarven mit bestimmtem Farbmuster.

(19) Welche Störgrößen können die Ergebnisse des Experiments mit den Libellenlarven verfälscht haben? (E3_*CVS_S)*

✏ Kreuze an!

Die Ergebnisse können durch folgende Störgrößen beeinflusst worden sein: Durch ...

- ☐ die Verwendung von zwei Attrappen, die sich in einer Eigenschaft (Farbe) unterscheiden.
- ☐ den Einsatz mehrerer Libellenlarven.
- ☐ die Fütterung einiger Versuchstiere.
- ☐ eine Eingewöhnungszeit im Wasserbecken für alle Versuchstiere.

Zum Schluss….

noch drei kurze Fragen, um einzuschätzen, wie schwierig du die Aufgaben in diesem Testheft findest. Bitte versuche, dich so genau wie möglich einzuschätzen. Mache immer nur ein Kreuz pro Frage.

1. Wie schwierig war es für dich die Aufgaben zu verstehen?					
☐	☐	☐	☐	☐	☐
1<br>einfach	2	3	4	5	6<br>sehr schwierig

2. Wie schwierig war es für dich mit diesem Testheft zu arbeiten?					
☐	☐	☐	☐	☐	☐
1<br>einfach	2	3	4	5	6<br>sehr schwierig

3. Wie sehr hast du dich bei der Bearbeitung der Aufgaben angestrengt?					
☐	☐	☐	☐	☐	☐
1<br>Wenig	2	3	4	5	6<br>sehr

NUN HAST DU ES FAST GESCHAFFT!

ÜBERPRÜFE NOCH EINMAL, OB DU ALLE 7 SEITEN BEARBEITET HAST! VIELEN DANK FÜR DEINE MITARBEIT!
