# Supplementary material for: Self-Generation in the Context of Inquiry-Based Learning
Source: Front Psychol. 2018 Dec 13;9:2440. doi: 10.3389/fpsyg.2018.02440 (PMC6315139; doi:10.3389/fpsyg.2018.02440)
Supplement: FIGURE S8 — Coding_scheme. [file Image_8.pdf]

Kodiermanual Studie 2

- Forscherhefte-

Teil I: Versuchsplanung A

Versuchsplanung und -skizze

Überlege dir einen Versuch, mit dem du Ellies Vermutung überprüfen kannst.
Notiere hierfür deine ersten Überlegungen. **[4 min]**

Meine ersten Überlegungen

Was testet man: Es soll der Einfluss von _________ auf das Verhalten der Wasserflöhe getestet werden.

Was misst man: Der Einfluss auf das Verhalten kann daran beobachtet/ gemessen werden, dass die Wasserflöhe Folgendes tun: ______________________

Wie kontrolliert man: Dass das Verhalten tatsächlich durch die Testgröße ausgelöst wurde, wird folgendermaßen kontrolliert: ______________________

____

Unabhängige Variable

Beispiel 1	Licht (*n* = 56)	**1**
Beispiel 2	UV-Licht (*n* = 1)	**1**
Beispiel 3	Licht und Dunkelheit (*n* = 1)	**1**

Abhängige Variable

Nennung der ***zu messenden Variable (AV)*** (z.B. Wasserflöhe meiden bzw. bevorzugen das Licht) UND/ODER **Quantifizierung** der zu **messenden Variable** (AV) (z.B. Anzahl der Wasserflöhe, die Licht meiden bzw. bevorzugen)		
**Beispiel 1**	Sie meiden das Licht/ verstecken sich davor. (*n*= 18)	**1**
**Beispiel 2**	Sie schwimmen von oder zum Licht. (*n*= 6)	**1**
Formulierung einer Antwort, die sich nicht direkt auf die zu messende Variable bezieht. (= falsche Antwort)		
**Beispiel 3**	Sie schwimmen tagsüber(/morgens) nach unten und nachts(/abends) nach oben. (*n* = 7)<br>→ wenn als unabhängige Variable **(UV-)Licht** genannt, dann	**0**<br>**1**
**Beispiel 4**	Sich oben oder unten aufhalten. (*n* = 1)<br>→ wenn als unabhängige Variable **(UV-)Licht** genannt, dann	**0**<br>**1**
**Beispiel 5**	Sie reagieren darauf. (*n* = 1)	**0**
**Beispiel 6**	Sie wandern. (*n* = 2)	**0**

Kontrolle

Gewährleistung einer **Auswahlmöglichkeit** für das Versuchstier **(z.B. entscheiden, aussuchen, bestimmen) zwischen hell und dunkel.**		
Beispiel 1	Sie können sich **zwischen hell und dunkel** entscheiden./ Sie **suchen sich aus**, wo sie hingehen. (n = 4)	**1**
Beispiel 2	Ein **heller und ein dunkler Raum**. (n = 4)	**1**
Die Antwort zeigt ein Verständnis, das logisch nicht schlüssig oder methodisch nicht korrekt ist. (= falsche Antwort)		
Beispiel 3	Nochmal das Experiment durchführen./ Experiment mehrmals machen (n = 4)	**0**
Beispiel 4	Es werden große und kleine Tiere im Testexperiment genutzt. (n = 1)	**0**

Teil I: Versuchsplanung B

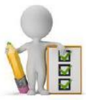

Besprecht eure ersten Überlegungen zur Versuchsplanung.
Legt gemeinsam die einzelnen Schritte eures Plans fest. **[10 min]**

Unser Plan

Schritt 1. Welche Materialien benötigt ihr?
☐ schachtelförmiger Behälter ☐ schwarzer Karton/schwarzes Tuch ☐ Taschenlampe
☐ LED-Lampe ☐ Pipette ☐ Stoppuhr ☐ Wasserflöhe ☐ Wasser

Schritt 2. Wie viele Wasserflöhe braucht ihr? ______________
Warum? ______________________________

Schritt 3. Wie genau kontrolliert ihr, dass das Licht einen Einfluss auf das Verhalten hat?
____
____

Schritt 1: nicht kodieren

Schritt 2:	→genannte Anzahl sollte größer gleich 10 sein	1

Schritt 2: Angemessener Stichprobenumfang

Formulierung einer Antwort, die ein Verständnis darüber zeigt, dass bei der Verwendung von mehreren Tieren die Ergebnisse besser **verallgemeinert (generalisiert)** werden können. Die Ergebnisse sind dadurch eher **repräsentativ** für eine Grundgesamtheit (Wasserflöhe allgemein).		
Beispiel 1	*Solche Experimente und Untersuchungen müssen **immer** mit mehreren Versuchspersonen oder Versuchstieren durchgeführt werden, weil man nur so ein **allgemeines** **und** **eindeutiges Ergebnis** erzielen kann.*	**2**
Beispiel 2	*Damit wir testen können, wo die **Mehrheit** hingeht. (n = 6)*	**2**
Formulierung einer Antwort, die ein Verständnis darüber zeigt, dass sich durch Untersuchung mehrerer Tiere die **Genauigkeit der Messung/Beobachtung** erhöht.		
Beispiel 3	*Damit das Ergebnis **eindeutig/ genau/ klarer** ist. (n = 21)*	**1**
Beispiel 4	*Weil es sein kann, dass es **Wasserflöhe gibt**, die gerade **keinen Hunger** **haben.*** **(Störgröße (Hunger) ausschließen)**	**1**
Die Antwort zeigt ein Verständnis, das logisch nicht schlüssig oder methodisch nicht korrekt ist. (= falsche Antwort)		
Beispiel 5	*Damit man sie sieht. (n = 7)*	**0**
Beispiel 6	*Damit man den Unterschied sieht. (n = 1)*	**0**

Schritt 3: Kontrolle

Gewährleistung einer **Auswahlmöglichkeit** für das Versuchstier **(z.B. entscheiden, aussuchen, bestimmen) zwischen hell und dunkel.**		
Beispiel 1	Die Wasserflöhe entscheiden lassen (heller vs. dunkler Raum) (*n* = 14)	**1**
Beispiel 2	Wir machen zwei verschiedene Hälften (hell/dunkel) und die Flöhe in die Mitte (*n*= 9)	**1**
Beispiel 3	Dass man das Gefäß mit einem Tuch auf einer Seite abdeckt. (*n* = 1)	**1**
Die Antwort zeigt ein Verständnis, das logisch nicht schlüssig oder methodisch nicht korrekt ist. (= falsche Antwort)		
Beispiel 4	Auf die Reaktionen bei Licht achten./ Gucken wie die Wasserflöhe auf Licht reagieren. (*n* = 9)	**0**
Beispiel 5	Einfach am Ende gucken. (*n* = 1)	**0**
Beispiel 6	Indem wir das Licht an und ausmachen. (*n* = 1)	**0**

Schritt 4. Was messt/ beobachtet ihr?

____

Und wie geht ihr dabei vor?

____

____

Schritt 5. Wie vermeidet ihr Störungen/ andere Einflüsse?

____

____

Schritt 4: Abhängige Variable

Was in dem Experiment gemessen wird.

Nennung der ***zu messenden Variable (AV)*** (z.B. Wasserflöhe meiden bzw. bevorzugen das Licht, Verhalten/ Reaktion der Wasserflöhe) UND/ODER **Quantifizierung** der zu **messenden Variable** (AV) (z.B. Anzahl der Wasserflöhe, die Licht meiden bzw. bevorzugen)		
Beispiel 1	Wir beobachten das Verhalten der Flöhe/ die Reaktion. (*n* = 13) ***(Reaktion als abhängige Variable)***	1
Beispiel 2	Ob sie zum hellen oder dunklen Bereich gehen/schwimmen. (*n* = 13)	1
Beispiel 3	Wo sie hinschwimmen. (n = 9)	1
Formulierung einer Antwort, die sich auf die variierte Variable oder eine Kontrollvariable bezieht. (= falsche Antwort)		
Beispiel 4	Wieso sie tagsüber nach unten gehen. (*n* = 1)	0
Beispiel 5	Das Verhalten messen, wie schnell sie nach oben kommen. (*n* = 1)	0

Wie gemessen werden kann.

Quantifizierung der zu messenden Variable (AV) (z.B. Anzahl der Wasserflöhe, die Licht meiden bzw. bevorzugen)		
Beispiel 1	Wie viele Wasserflöhe befinden sich in welchem Bereich (zu welcher Zeit).	1
Beispiel 2	Wir geben denen 1 Minute Zeit und schauen wo sie hin schwimmen.	1
Beispiel 3	"Wie Schritt drei" (n = 6)<br>→ wenn in Schritt 3 Quantifizierung angegeben wurde	1
Formulierung einer Antwort, die sich nicht direkt auf die zu messende Variable bezieht. (= falsche Antwort)		
Beispiel 4	Erstmal mit der Taschenlampe ins Wasser leuchten. (n = 1)	0
Beispiel 5	Man installiert eine Kamera oder schaut einfach. (n = 1)	0

Schritt 5: Kontrolle

Nennung von **Störvariablen** (z.B. Lärm, Erschütterungen, andere Lichtquellen) **bzw. Nicht-Konstanthaltung bestimmter Kontrollvariablen** (z.B. Gemütszustand, Hungerzustand der Tiere, Abstand, Größe des hellen und dunklen Bereichs)		
Beispiel 1	Sie nicht stören/in Ruhe lassen und nicht zu nahe kommen. (n=11)<br>Keine ruckartigen Bewegungen/keine Erschütterungen. (n=14)	1
Beispiel 2	Alle gleichzeitig füttern. (n=3)	1
Beispiel 3	Licht konstant halten (n=6) /keine anderen Lichtquellen/das kein anderes Licht scheint. (n=9)	1
Formulierung einer Antwort, die sich nicht direkt auf Störvariablen bezieht/ logisch nicht schlüssig ist. (= falsche Antwort)		
Beispiel 4	Alle mit Licht bestrahlen.	0
Beispiel 5	Gucken, ob sie nach unten schwimmen oder ob sie, wenn wir die Taschenlampe wieder aus machen, nach oben schwimmen werden. (n=1)	0
Beispiel 6	Wir stellen uns dahin, wo Licht ist. (n=5)	0

Teil II: Fehleranalyse

Rückblick auf das Experiment

Geht die folgende Checkliste durch und hakt all die Punkte ab, die ihr bei eurer Durchführung beachtet habt. **[7 min]**

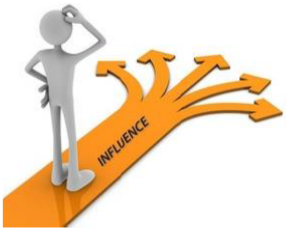

Unsere Analyse

Tierischer Einfluss:

☐ Eingewöhnungszeit der Wasserflöhe (ca. 2 min) beachtet

☐ Eigenheiten eines Lebewesens beachtet (Hunger, Gemütszustand usw.)

WARUM?

____

____

Kontrolle Vgl. *(E2_Was_F_O)*

Nennung der **Konstanthaltung** der Versuchsbedingungen **(z.B. gleiche Ausgangslage)**		
Beispiel 1	Es wäre sinnvoll (Essen zu geben), weil sonst suchen sie sich Essen. (n = 3) (***Auswirkung bei Nicht-Konstanthaltung)***	2
Beispiel 2	Wenn sie Hunger haben, schwimmen sie zu der helleren Seite. (n = 1) (***Auswirkung bei Nicht-Konstanthaltung)***	2
**INDIREKTE** Nennung der **Konstanthaltung** der Versuchsbedingungen <u>ODER</u> Gewährleistung einer **Auswahlmöglichkeit** für das Versuchstier **(z.B. entscheiden, aussuchen, bestimmen)**		
Beispiel 3	Manche hatten Hunger, manche nicht. Algen wachsen im Licht. ***(Konstanthaltung indirekt)***	1
Beispiel 4	Weil sie sich an die Umgebung gewöhnen müssen, um nicht durch sie beeinflusst zu werden. ***(Konstanthaltung)***	1
Formulierung einer Antwort, die kein Verständnis für die Notwendigkeit der Kontrolle der Versuchsbedingung „Raum/Größe" zeigt. Die Antwort zeigt ein Verständnis, das logisch nicht schlüssig oder methodisch nicht korrekt ist. (= falsche Antwort)		
Beispiel 5	Weil man es nicht feststellen/messen konnte. (n = 5)	0
Beispiel 6	Um das Experiment richtig durchzuführen. (n = 1)	0

Äußerer Einfluss:

☐ äußeren Lichteinfall beachtet (Fenster, Raumlicht)

☐ Ruhe im Klassenraum

☐ möglichst geringe (bis keine) Lichtspiegelung in der Schachtel

Menschlicher Einfluss:

☐ **dunklen und hellen Wasserbereich gleich groß gehalten** → **WARUM?**

Weil____

☐ **ausreichend Wasserflöhe (ca. 10 oder mehr) verwendet** → **WARUM?**

Weil____

☐ **LED-Lampe statt Taschenlampe verwendet** → **WARUM?**

Weil____

☐ keine Veränderungen nach Durchführungsbeginn

☐ keine Stöße gegen den Tisch

Kontrolle

Vgl. *(E2_Was_F_O)*

Nennung der **Konstanthaltung** der Versuchsbedingungen **(z.B. Abstand/Raum/Größe/Platz)**		
Beispiel 1	*Damit es **gleiche Chancen** für beide Seiten sind, sonst befinden sich mehr im größeren Teil. **(Konstanthaltung)***	2
Beispiel 2	*Wenn mehr als die Hälfte abgedunkelt wäre, dann sind die Flöhe eher im dunklen Bereich, weil **dort mehr Platz** ist und weniger Licht rein scheint.**(Auswirkung bei Nicht-Konstanthaltung)***	2
**INDIREKTE** Nennung der **Konstanthaltung** der Versuchsbedingungen ODER Gewährleistung einer **Auswahlmöglichkeit** für das Versuchstier **(z.B. entscheiden, aussuchen, bestimmen)**		
Beispiel 3	*Damit die **Reaktion der Wasserflöhe nicht beeinflusst** wird. **(Konstanthaltung indirekt)***	1
Beispiel 4	*Damit man sehen kann, wo sie sich **wohler fühlen**, im Hellen oder im Dunklen.* **(Gewährleistung einer Auswahlmöglichkeit für das Versuchstier durch Ausprobieren)**	1
Formulierung einer Antwort, die kein Verständnis für die Notwendigkeit der Kontrolle der Versuchsbedingung „Raum/Größe" zeigt. Die Antwort zeigt ein Verständnis, das logisch nicht schlüssig oder methodisch nicht korrekt ist. (= falsche Antwort)		
Beispiel 5	*Weil wir Tag und Nacht simulieren wollten.*	0
Beispiel 6	*Weil wir gucken wollten, wie viele im Dunkeln sind und im Hellen.*	0

Angemessener Stichprobenumfang — Vgl. *(E3_Was_T_O)*

Formulierung einer Antwort, die ein Verständnis darüber zeigt, dass bei der Verwendung von mehreren Tieren die Ergebnisse besser **verallgemeinert (generalisiert)** werden können. Die Ergebnisse sind dadurch eher **repräsentativ** für eine Grundgesamtheit (Wasserflöhe allgemein).		
Beispiel 1	*Damit er bessere Ergebnisse hat und **mehr Bestätigungen**. / Desto mehr man da rein macht, desto besser kann man die **Mehrheit** erkennen./ Weil man dann ein `**vielfältiges'** Ergebnis hat.*	2
Beispiel 2	*Es werden mehrere Asseln eingesetzt, damit das Experiment **nicht aus Zufall** entschieden wird./ Es kann sein, dass eine keine Reaktion Zeit und deswegen werden mehrere benutzt, damit die eine zeigen.*	2
Formulierung einer Antwort, die ein Verständnis darüber zeigt, dass sich durch Untersuchung mehrerer Tiere die **Genauigkeit der Messung/Beobachtung** erhöht.		
Beispiel 3	*Damit das Ergebnis **eindeutiger** ist.*	1
Beispiel 4	*Bei mehreren ist das Ergebnis **genauer**./ Damit man **genauer** weiß, ob die Wasserflöhe dunkle oder helle Gebiete bevorzugen.*	1
Die Antwort zeigt ein Verständnis, das logisch nicht schlüssig oder methodisch nicht korrekt ist. (= falsche Antwort)		
Beispiel 5	*Dass es gleichmäßig ist.*	0
Beispiel 6	*Weil sich Wasserflöhe in Gruppen besser fühlen als alleine./ Weil es mal sein kann, dass ein Floh krank ist und nur so im Licht schwimmt.*	0

Kontrolle — Vgl. *(E1_Was_P_O)*

Nennung der **Störgröße (Wärme/Hitze)** UND der möglichen **Beeinflussung der AV** (Verhalten/ Bewegung der Wasserflöhe) **als solche** im Experiment		
Beispiel 1	*Gewöhnliche Taschenlampen **erhitzen** sich bei längerer Benutzung. Das könnte das **Verhalten der Flöhe beeinflussen**. **(Auswirkung:** Beeinflussung der Ergebnisse durch Faktor Wärme **wird genannt)***	2
Beispiel 2	*Eine gewöhnliche Taschenlampe **erzeugt Wärme**, somit könnte das ein **Störfaktor** sein. **(Als Störgröße erkannt)***	2
Nennung der **Störgröße (Wärme/Hitze)** ODER der möglichen **Beeinflussung der AV** (Verhalten/ Bewegung der Wasserflöhe) **als solche** im Experiment		
Beispiel 3	*Weil die LED Lampe keine **Wärme** erzeugt. **(Auswirkung:** Beeinflussung der Ergebnisse durch Faktor Wärme **wird nicht genannt)***	1
Beispiel 4	*Eine normale Taschenlampe **erwärmt** das Licht, die LED Lampe tut das nicht. Darum wurde die LED Lampe benutzt.*	1
Die Antwort zeigt ein Verständnis, das logisch nicht schlüssig oder methodisch nicht korrekt ist. (= falsche Antwort)		
Beispiel 5	*Weil die LED Lampe heller ist.*	0
Beispiel 6	*Weil die LED Lampe weißes Licht hat und die normale Taschenlampe ist etwas gelber.*	0

Teil III: Lückentext

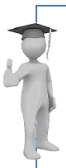

Ich habe über das Experimentieren folgendes gelernt:

Mit unserem Experiment haben wir den Einfluss von Licht auf das Verhalten der Wasserflöhe untersucht (Testgröße). Dass das Verhalten tatsächlich durch das Licht ausgelöst wurde, haben wir kontrolliert, indem wir den Wasserflöhen einen hellen und einen dunklen Bereich zur Verfügung stellten. Die Größe beider Bereiche war gleich.

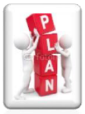

Die Wasserflöhe hatten dadurch die Möglichkeit zwischen Licht und Dunkelheit zu wählen.
Beobachtet wurde, in welchen Bereich die Wasserflöhe geschwommen sind.
Hierbei haben wir notiert, wie viele Wasserflöhe sich in einer bestimmten Zeitspanne im hellen und dunklen Bereich befanden (Messgröße).
Wir haben möglichst viele Wasserflöhe eingesetzt, um einen Zufall unserer Ergebnisse auszuschließen.

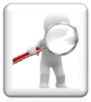

Störgrößen, die die tatsächliche Wirkung des Lichts auf das Verhalten der Wasserflöhe überdeckt haben könnten, sind z.B. folgende:
Tierischer Einfluss: z.B. Eingewöhnungszeit und Hungerzustand der Wasserflöhe
Äußerer Einfluss: z.B. Lichtspiegelungen, Lautstärke im Klassenraum
Menschlicher Einfluss: z.B. keine Veränderungen nach Durchführungsbeginn (wie Stöße gegen den Tisch/ Änderung der Position der Taschenlampe).

Ich habe über das Experimentieren folgendes gelernt:

Mit unserem Experiment haben wir den Einfluss von ________________auf das Verhalten der Wasserflöhe untersucht (Testgröße). Dass das Verhalten tatsächlich durch das Licht ausgelöst wurde, haben wir ________________, indem wir den Wasserflöhen einen ________________ und einen ________________Bereich zur Verfügung stellten.

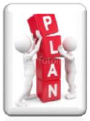

Die Größe beider Bereiche war ________________.
Die Wasserflöhe hatten dadurch die Möglichkeit zwischen Licht und ________________zu wählen.
Beobachtet wurde, in welchen Bereich die Wasserflöhe ________________ sind.
Hierbei haben wir notiert, ________________Wasserflöhe sich in einer bestimmten Zeitspanne im ________________ und ________________Bereich befanden (Messgröße).
Wir haben möglichst ________________ Wasserflöhe eingesetzt, um einen Zufall unserer Ergebnisse auszuschließen.

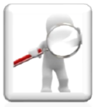

________________, die die tatsächliche Wirkung des Lichts auf das Verhalten der Wasserflöhe überdeckt haben könnten, sind z.B. folgende:
Tierischer Einfluss: z.B. ________________________________
Äußerer Einfluss: z.B. ________________________________
Menschlicher Einfluss: z.B. ________________________________
____

L1	Licht/UV-Licht/Licht und Dunkelheit	1
L2	kontrolliert/getestet/gesehen	1
L3	hellen bzw. dunklen	1
L4	dunklen bzw. hellen	1
L5	gleich/gleich groß/ halbe halbe/zur Hälfte dunkel/hell	1
L6	Dunkel/Dunkelheit/Schatten/Finsternis/kein Licht	1
L7	geschwommen/hingegangen/ gehen/ gewandert/ gegangen	1
L8	wie viele	1
L9	dunklen bzw. hellen	1
L10	hellen bzw. dunklen	1
L11	Vvele/mehrere/eine große Anzahl	1
L12	Störgrößen/Störungen/Einflüsse/Einflussgrößen	1
L13	Eingewöhnungszeit/Hunger bzw. gefüttert und ungefüttert/ Kommunikation/ Gemütszustand bzw. Krankheit	Je 1
L14	Lichtspiegelungen/ Lautstärke im Klassenraum bzw. Geräusche/unterschiedlich große Bereiche von hell und dunkel	Je 1
L15	Stöße/ Taschenlampenposition verändert/ Eingewöhnungszeit/ Lautstärke/ unterschiedlich große Bereiche von hell und dunkel	Je 1
