# Supplementary material for: Self-Generation in the Context of Inquiry-Based Learning
Source: Front Psychol. 2018 Dec 13;9:2440. doi: 10.3389/fpsyg.2018.02440 (PMC6315139; doi:10.3389/fpsyg.2018.02440)

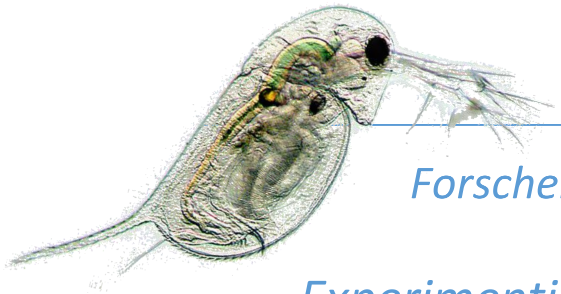

Forschen wie ein Biologe!

- Experimentieren mit Wasserflöhen

Name: ________________________________ Schülercode: ________________________________

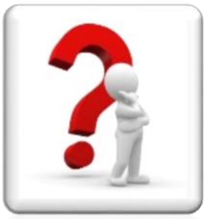

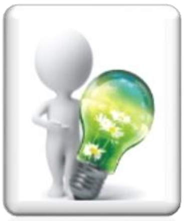

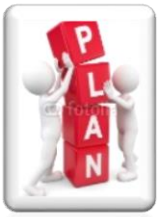

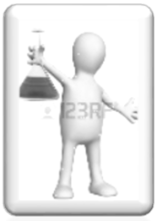

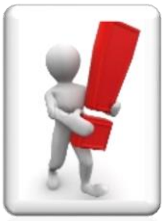

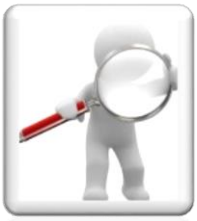

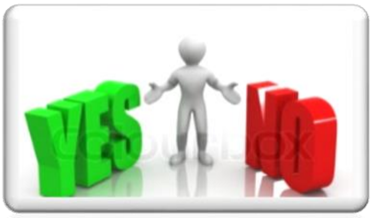

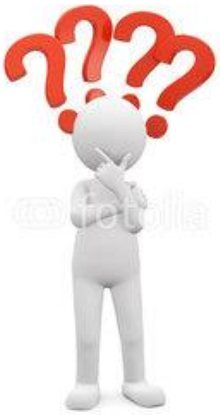

Forschungsfrage

Forschungsfrage:

Wie reagieren Wasserflöhe auf helle und dunkle Lichtverhältnisse?

[10 min]

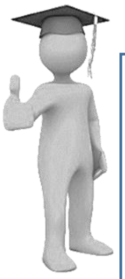

Ich habe über das Experimentieren folgendes gelernt:

Mit unserem Experiment haben wir den Einfluss von ________________auf das Verhalten der Wasserflöhe untersucht (Testgröße). Dass das Verhalten tatsächlich durch das Licht ausgelöst wurde, haben wir ________________, indem wir den Wasserflöhen einen ________________ und einen ________________Bereich zur Verfügung stellten.

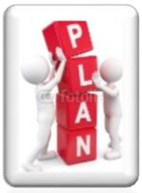

Die Größe beider Bereiche war ________________.
Die Wasserflöhe hatten dadurch die Möglichkeit zwischen Licht und ________________zu wählen.
Beobachtet wurde, in welchen Bereich die Wasserflöhe ________________ sind.
Hierbei haben wir notiert, ________________Wasserflöhe sich in einer bestimmten Zeitspanne im ________________ und ________________Bereich befanden (Messgröße).
Wir haben möglichst ________________ Wasserflöhe eingesetzt, um einen Zufall unserer Ergebnisse auszuschließen.

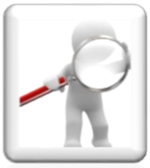

________________, die die tatsächliche Wirkung des Lichts auf das Verhalten der Wasserflöhe überdeckt haben könnten, sind z.B. folgende:
Tierischer Einfluss: z.B. ____
Äußerer Einfluss: z.B. ____
Menschlicher Einfluss: z.B. ____
____

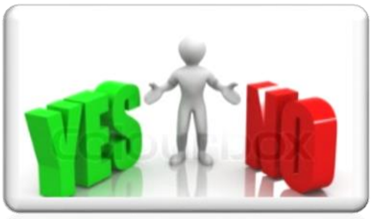

Vermutungsüberprüfung

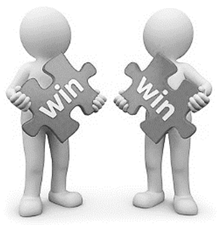

Durch den Versuch weiß ich nun, dass Ellies Vermutung

- ○ zutrifft
- ○ nicht zutrifft

Informiert euch kurz über das Phänomen: **[3 min]**

Die geheimnisvolle Wanderung

Jeden Tag machen die Wasserflöhe im Teich eine geheimnisvolle Wanderung. Am Abend wandern sie aus der Tiefe des Teichs nach oben, am nächsten Morgen verschwinden sie wieder nach unten.
Ein Rätsel, das die Wissenschaftler lange Zeit ratlos gemacht hat.
Heute ist es an euch, dieses Rätsel zu lüften!

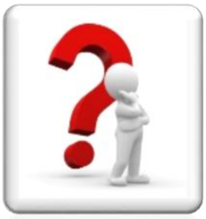

Vermutung mit Begründung

Ellie und Tom überlegen, wie Wasserflöhe auf Hell und Dunkel reagieren. **[6 min]**

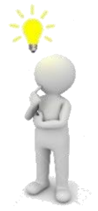

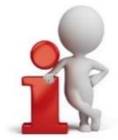

Ellies und Toms Vermutungen:

Ellie ist der festen Überzeugung, dass Wasserflöhe Licht meiden, Tom hat da seine Zweifel. Er glaubt, dass Wasserflöhe Licht bevorzugen.

Tom: „Ellie, das macht doch gar keinen Sinn, dass Wasserflöhe das Licht meiden! Wasserflöhe fressen Grünalgen. Und diese wachsen nur in hellen Wasserschichten. Warum sollten Wasserflöhe dunkle Wasserschichten bevorzugen, in denen es keine Algen gibt, von denen sie sich ernähren können?"

Ellie: „Aber Licht ist total schädlich für Wasserflöhe. Weißt du denn nicht, dass sie eine durchsichtige Schale haben? Sie können sich doch gar nicht vor UV-Licht schützen und außerdem jagen Fische und andere Fressfeinde in den hellen Wasserschichten nach Wasserflöhen. Im Hellen können sie ihre Beute viel besser erkennen! Damit besteht doppelte Gefahr!"

Äußerer Einfluss:

☐ äußeren Lichteinfall beachtet (Fenster, Raumlicht)

☐ Ruhe im Klassenraum

☐ möglichst geringe (bis keine) Lichtspiegelung in der Schachtel

Menschlicher Einfluss:

☐ **dunklen und hellen Wasserbereich gleich groß gehalten** → **WARUM?**

Weil____

☐ **ausreichend Wasserflöhe (ca. 10 oder mehr) verwendet** → **WARUM?**

Weil____

☐ **LED-Lampe statt Taschenlampe verwendet** → **WARUM?**

Weil____

☐ keine Veränderungen nach Durchführungsbeginn

☐ keine Stöße gegen den Tisch

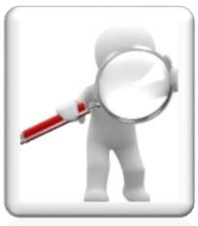

Rückblick auf das Experiment

Geht die folgende Checkliste durch und hakt all die Punkte ab, die ihr bei eurer Durchführung beachtet habt. **[7 min]**

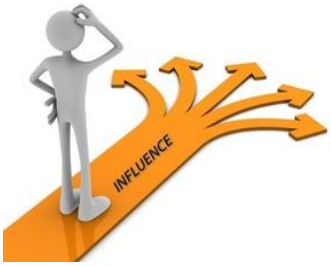

Unsere Analyse

Tierischer Einfluss:

☐ Eingewöhnungszeit der Wasserflöhe (ca. 2 min) beachtet

☐ Eigenheiten eines Lebewesens beachtet (Hunger, Gemütszustand usw.)

WARUM?

____

____

Ellie und Tom einigen sich auf eine Vermutung: **[4 min]**

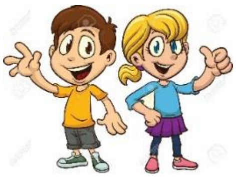

Gemeinsame Vermutung:

Da Ellie und Tom gemeinsam ein Experiment planen möchten, welches sie am nächsten Tag unabhängig voneinander durchführen, wollen sie versuchen, sich auf eine Vermutung zu einigen:

Tom: „ Also gut Ellie, an deiner Vermutung ist etwas dran. Wenn UV-Licht für Wasserflöhe so schädlich ist und sich Fressfeinde in der oberen hellen Wasserschicht befinden, halten sich Wasserflöhe bestimmt nicht allzu gerne im Hellen auf. Doch so ganz überzeugt bin ich nicht, denn auf ihre Nahrung können sie auch nicht verzichten. Also will ich erst Beweise sehen!“

Ellie: „Dann lass es uns über ein Experiment herausfinden!“

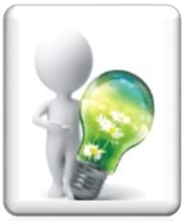

Versuchsplanung und -skizze

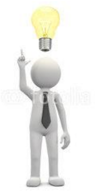

Überlege dir einen Versuch, mit dem du Ellies Vermutung überprüfen kannst.
Notiere hierfür deine ersten Überlegungen. **[4 min]**

Meine ersten Überlegungen

Was testet man: Es soll der Einfluss von _________ auf das Verhalten der Wasserflöhe getestet werden.

Was misst man: Der Einfluss auf das Verhalten kann daran beobachtet/ gemessen werden, dass die Wasserflöhe Folgendes tun: ______________________________

Wie kontrolliert man: Dass das Verhalten tatsächlich durch die Testgröße ausgelöst wurde, wird folgendermaßen kontrolliert: ______________________________

____

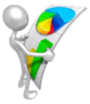

Findet heraus, zu wessen Ergebnissen eure Ergebnisse passen und setzt ein Kreuz. **[10 min]**

**Ellie** fand heraus, dass sich die gesamte Zeit über die Mehrheit der Wasserflöhe im Dunkeln befand. Mit diesen Ergebnissen konnte sie ihre anfängliche Vermutung bestätigen.<br>Ellie: „Ha, ich hab es Tom doch gleich gesagt! Wasserflöhe meiden das Licht natürlich, da ihre Schale durchsichtig ist und somit keine Farbstoffe enthält, die sie vor ultravioletter Strahlung, einem bestimmten Anteil des Sonnenlichts, schützt. Die UV-Strahlung ist vor allem an der Wasseroberfläche besonders hoch und nimmt mit zunehmender Tiefe ab. So gehen Wasserflöhe im Sommer, wenn die Sonneneinstrahlung zunimmt, auf Tauchstation: Tagsüber sind sie dann in den tieferen Wasserschichten verborgen und können dadurch der schädlichen Strahlung und den Fressfeinden auszuweichen.“<br>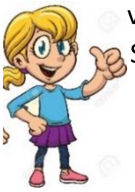<br>ELLIES DEUTUNG ☐	**Tom** fand heraus, dass sich die gesamte Zeit über die Mehrheit der Wasserflöhe im Hellen befand. Mit diesen Ergebnissen konnte er seine anfängliche Vermutung bestätigen und Ellies Vermutung widerlegen.<br>Tom: „Ich hab`s doch gleich gewusst! Wie sollen Wasserflöhe auch ohne Licht auskommen? Sie ernähren sich von winzigen schwebenden Grünalgen, die Licht für ihr Wachstum benötigen und deshalb nur in den hellen Schichten eines Sees oder Teichs vorkommen. Über ihr Komplexauge, das Hell und Dunkel unterscheiden kann, können Wasserflöhe ihre Nahrung nur in den oberen, hellen Schichten finden. Folglich dient Licht den Wasserflöhen als Orientierung bei der Nahrungssuche.“<br>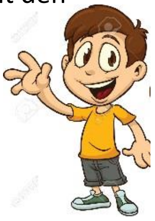<br>☐ TOMS DEUTUNG

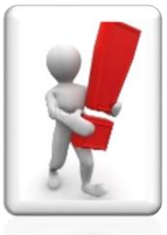

Versuchsauswertung und Ergebnisdeutung

Notiere deine Beobachtungen und versuche diese möglichst einfach zu veranschaulichen.
[10 min]

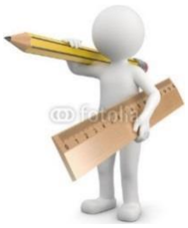

Meine Auswertung:

Anzahl der Wasserflöhe	*hell*	*dunkel*
nach … min		
nach …min		
nach … min		
nach …min		
nach … min		
nach … min		

Präsentiere den anderen Gruppenmitgliedern deine Versuchsauswertung. **[5 min]**

Verschaffe dir einen Überblick über das Material, das euch für den Versuch zur Verfügung steht. **[3 min]**

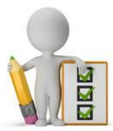

Besprecht eure ersten Überlegungen zur Versuchsplanung.
Legt gemeinsam die einzelnen Schritte eures Plans fest. **[10 min]**

Unser Plan

Schritt 1. Welche Materialien benötigt ihr?

☐ schachtelförmiger Behälter ☐ schwarzer Karton/schwarzes Tuch ☐ Taschenlampe

☐ LED-Lampe ☐ Pipette ☐ Stoppuhr ☐ Wasserflöhe ☐ Wasser

Schritt 2. Wie viele Wasserflöhe braucht ihr? ____________________

Warum? ____

Schritt 3. Wie genau kontrolliert ihr, dass das Licht einen Einfluss auf das Verhalten hat?

____

____

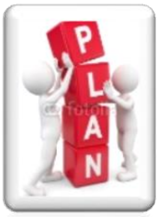

Versuchsplanung & -skizze

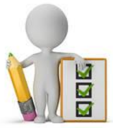

Schritt 4. Was messt/ beobachtet ihr?

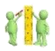

____

Und wie geht ihr dabei vor?

____

____

Schritt 5. Wie vermeidet ihr Störungen/ andere Einflüsse?

____

____

Überarbeitet eure Versuchsplanung und fertigt nun gemeinsame eine Skizze an, die alle wichtigen Eigenschaften eurer Versuchsplanung abbildet. **[5 min]**

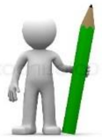

Unsere Skizze

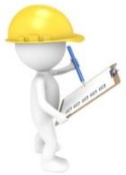

Führt euren Versuch nun wie geplant durch. Achtet auf mögliche Störquellen! **[20 min]**

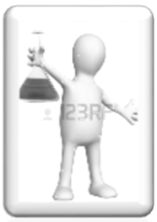

Supplement: FIGURE S9 — Research workbook_GF. [file Image_9.pdf]
