# Supplementary material for: Self-Generation in the Context of Inquiry-Based Learning
Source: Front Psychol. 2018 Dec 13;9:2440. doi: 10.3389/fpsyg.2018.02440 (PMC6315139; doi:10.3389/fpsyg.2018.02440)

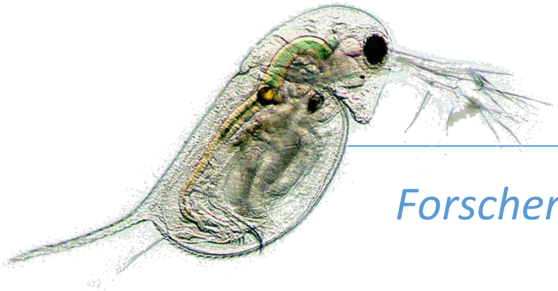

Forschen wie ein Biologe!

- Experimentieren mit Wasserflöhen

Name: ________________________________ Schülercode: ________________________________

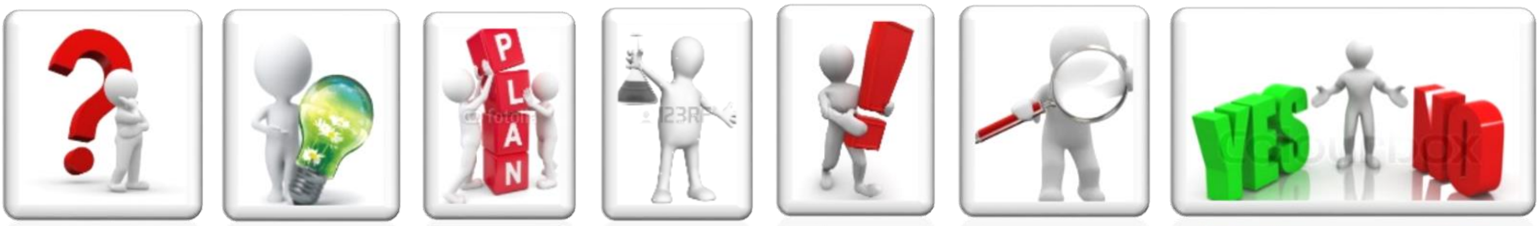

Forschungsfrage

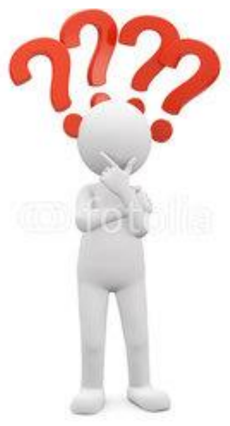

Forschungsfrage:

Wie reagieren Wasserflöhe auf helle und dunkle Lichtverhältnisse?

[10 min]

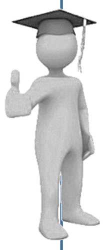

Ich habe über das Experimentieren folgendes gelernt:

Mit unserem Experiment haben wir den Einfluss von Licht auf das Verhalten der Wasserflöhe untersucht (Testgröße). Dass das Verhalten tatsächlich durch das Licht ausgelöst wurde, haben wir kontrolliert, indem wir den Wasserflöhen einen hellen und einen dunklen Bereich zur Verfügung stellten. Die Größe beider Bereiche war gleich.

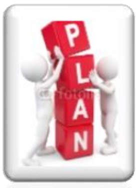

Die Wasserflöhe hatten dadurch die Möglichkeit zwischen Licht und Dunkelheit zu wählen.

Beobachtet wurde, in welchen Bereich die Wasserflöhe geschwommen sind.

Hierbei haben wir notiert, wie viele Wasserflöhe sich in einer bestimmten Zeitspanne im hellen und dunklen Bereich befanden (Messgröße).

Wir haben möglichst viele Wasserflöhe eingesetzt, um einen Zufall unserer Ergebnisse auszuschließen.

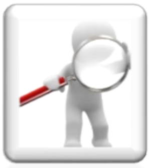

Störgrößen, die die tatsächliche Wirkung des Lichts auf das Verhalten der Wasserflöhe überdeckt haben könnten, sind z.B. folgende:

Tierischer Einfluss: z.B. Eingewöhnungszeit und Hungerzustand der Wasserflöhe

Äußerer Einfluss: z.B. Lichtspiegelungen, Lautstärke im Klassenraum

Menschlicher Einfluss: z.B. keine Veränderungen nach Durchführungsbeginn (wie Stöße gegen den Tisch/ Änderung der Position der Taschenlampe).

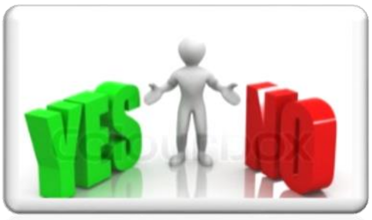

Stimmt Ellies Vermutung?

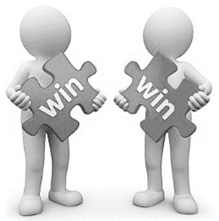

Durch den Versuch weiß ich nun, dass Ellies Vermutung

- ○ zutrifft
- ○ nicht zutrifft

Informiert euch kurz über das Phänomen: **[3 min]**

Die geheimnisvolle Wanderung

Jeden Tag machen die Wasserflöhe im Teich eine geheimnisvolle Wanderung. Am Abend wandern sie aus der Tiefe des Teichs nach oben, am nächsten Morgen verschwinden sie wieder nach unten.
Ein Rätsel, das die Wissenschaftler lange Zeit ratlos gemacht hat.
Heute ist es an euch, dieses Rätsel zu lüften!

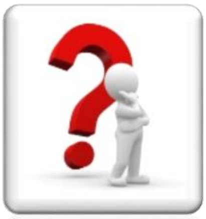

Vermutung

Ellie und Tom überlegen, wie Wasserflöhe auf Hell und Dunkel reagieren. **[6 min]**

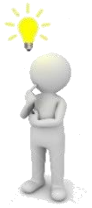

Ellies und Toms Vermutungen:

Ellie ist der festen Überzeugung, dass Wasserflöhe Licht meiden, Tom hat da seine Zweifel. Er glaubt, dass Wasserflöhe Licht bevorzugen.

Tom: „ Ellie, das macht doch gar keinen Sinn, dass Wasserflöhe das Licht meiden! Wasserflöhe fressen Grünalgen. Und diese wachsen nur in hellen Wasserschichten. Warum sollten Wasserflöhe dunkle Wasserschichten bevorzugen, in denen es keine Algen gibt, von denen sie sich ernähren können? “

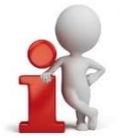

Ellie: „ Aber Licht ist total schädlich für Wasserflöhe. Weißt du denn nicht, dass sie eine durchsichtige Schale haben? Sie können sich doch gar nicht vor UV-Licht schützen und außerdem jagen Fische und andere Fressfeinde in den hellen Wasserschichten nach Wasserflöhen. Im Hellen können sie ihre Beute viel besser erkennen! Damit besteht doppelte Gefahr!“

Äußerer Einfluss:

…. wenn z.B. der äußere Lichteinfall (Fenster, Raumlicht), möglichst geringe(bis keine) Lichtspiegelung in der Schachtel, Ruhe im Klassenraum nicht sichergestellt wurden.

Menschlicher Einfluss:

…. wenn z.B. die Gleichhaltung des dunklen und hellen Wasserbereich, Verwendung ausreichend vieler Wasserflöhe (ca. 10 oder mehr), keine Veränderungen nach Durchführungsbeginn (wie Stöße gegen den Tisch, Änderung der Position der Taschenlampe) nicht garantiert werden können.

Gibt es einen solchen Einfluss auf eure Versuchsergebnisse, müsst ihr eure Ergebnisse hinterfragen. Denn diese Einflüsse können die wahre Wirkung der Testgröße überlagern.

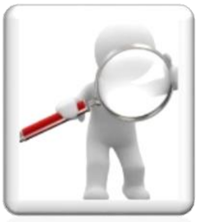

Rückblick auf das Experiment

Man kann sich auf die Ergebnisse eines Experiments nicht immer verlassen. Denn manchmal gibt es kleine Unaufmerksamkeiten, die eure Ergebnisse schnell verfälschen können: **[7 min]**

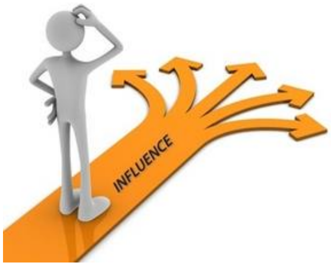

Ergebnisprüfung:

Weitere Größe, die die Ergebnisse prägen können, sind Störgrößen. Das sind alle Einflüsse, die neben der Testgröße auf das Verhalten der Wasserflöhe einwirken können. Es gibt verschiedene Arten von Störgrößen:

Tierischer Einfluss:

…. wenn z.B. eine Eingewöhnungszeit der Wasserflöhe (ca. 2 min), Eigenheiten eines Lebewesens (Hunger, Gemütszustand usw.) nicht beachtet wurden.

Ellie und Tom einigen sich auf eine Vermutung: **[4 min]**

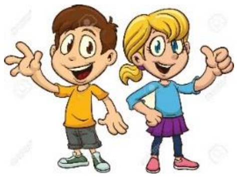

Gemeinsame Vermutung:

Da Ellie und Tom gemeinsam ein Experiment planen möchten, welches sie am nächsten Tag unabhängig voneinander durchführen, wollen sie versuchen, sich auf eine Vermutung zu einigen:

Tom: „ Also gut Ellie, an deiner Vermutung ist etwas dran. Wenn UV-Licht für Wasserflöhe so schädlich ist und sich Fressfeinde in der oberen hellen Wasserschicht befinden, halten sich Wasserflöhe bestimmt nicht allzu gerne im Hellen auf. Doch so ganz überzeugt bin ich nicht, denn auf ihre Nahrung können sie auch nicht verzichten. Also will ich erst Beweise sehen!“

Ellie: „Dann lass es uns über ein Experiment herausfinden!“

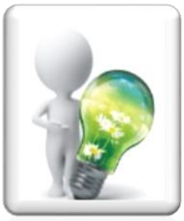

Versuchsplanung & -skizze

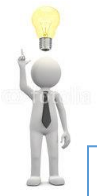

Ellie und Tom haben die Test- und Messgröße sowie den Kontrollansatz für ihren Versuch folgendermaßen bestimmt: **[4 min]**

Ellies und Toms ersten Überlegungen

Was testet man: Es soll der Einfluss von Licht auf das Verhalten der Wasserflöhe getestet werden.

Was misst man: Der Einfluss auf das Verhalten kann daran beobachtet/ gemessen werden, dass die Wasserflöhe Folgendes tun: Sie wandern vom Licht weg.

Wie kontrolliert man: Dass das Verhalten tatsächlich durch die Testgröße ausgelöst wurde, wird folgendermaßen kontrolliert: Die Wasserflöhe können zwischen einem hellen und einem gleichgroßen dunklen Bereich wählen.

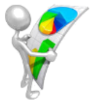

Findet heraus, zu wessen Ergebnissen eure Ergebnisse passen und setzt ein Kreuz. **[10 min]**

Ellie fand heraus, dass sich die gesamte Zeit über die Mehrheit der Wasserflöhe im Dunkeln befand. Mit diesen Ergebnissen konnte sie ihre anfängliche Vermutung bestätigen.

Ellie: „Ha, ich hab es Tom doch gleich gesagt! Wasserflöhe meiden das Licht natürlich, da ihre Schale durchsichtig ist und somit keine Farbstoffe enthält, die sie vor ultravioletter Strahlung, einem bestimmten Anteil des Sonnenlichts, schützt. Die UV-Strahlung ist vor allem an der Wasseroberfläche besonders hoch und nimmt mit zunehmender Tiefe ab. So gehen Wasserflöhe im Sommer, wenn die Sonneneinstrahlung zunimmt, auf Tauchstation: Tagsüber sind sie dann in den tieferen Wasserschichten verborgen und können dadurch der schädlichen Strahlung und den Fressfeinden auszuweichen."

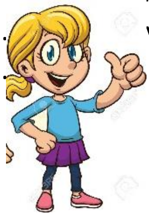

ELLIES DEUTUNG ☐

Tom fand heraus, dass sich die gesamte Zeit über die Mehrheit der Wasserflöhe im Hellen befand. Mit diesen Ergebnissen konnte er seine anfängliche Vermutung bestätigen und Ellies Vermutung widerlegen.

Tom: „Ich hab`s doch gleich gewusst! Wie sollen Wasserflöhe auch ohne Licht auskommen? Sie ernähren sich von winzigen schwebenden Grünalgen, die Licht für ihr Wachstum benötigen und deshalb nur in den hellen Schichten eines Sees oder Teichs vorkommen. Über ihr Komplexauge, das Hell und Dunkel unterscheiden kann, können Wasserflöhe ihre Nahrung nur in den oberen, hellen Schichten finden. Folglich dient Licht den Wasserflöhen als Orientierung bei der Nahrungssuche."

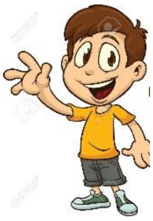

☐ TOMS DEUTUNG

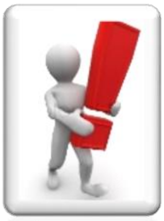

Versuchsauswertung und Ergebnisdeutung

Notiere deine Beobachtungen in der vorgegeben Tabelle. **[10 min]**

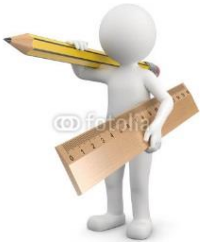

Meine Auswertung:

Anzahl der Wasserflöhe	*hell*	*dunkel*
nach ... min		
nach ...min		
nach ... min		
nach ... min		
nach ... min		
nach ... min		

Präsentiere den anderen Gruppenmitgliedern deine Versuchsauswertung. **[5 min]**

Verschafft euch einen Überblick über das Material, das euch für euren Versuch zur Verfügung steht. **[10 min]**

Lest euch die Versuchsplanung aufmerksam durch. **[4 min]**

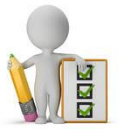

Ellies und Toms Plan

Schritt 1. Folgende Materialien beschaffen: schachtelförmiger Behälter, LED-Lampe, Pipette, schwarzer Karton, Stoppuhr, 10 Wasserflöhe, Wasser. ☐

Schritt 2. Behälter zu $^{3}/_{4}$ mit Wasser füllen und 10 Wasserflöhe hineinsetzen. ☐

Schritt 3. Genau die Hälfte des Behälters mit schwarzem Karton abdecken (Testgröße: Licht, Kontrollansatz: $\frac{1}{2}$ dunkler Bereich). ☐
Andere Hälfte des Behälters 10 min seitlich mit der Taschenlampe (Abstand: ca. eine Fingerlänge) bestrahlen.

Schritt 4. Notieren, wie viele Wasserflöhe sich pro Minute im abgedunkelten bzw. hellen Bereich befinden.
(Messgröße: Anzahl der Wasserflöhe pro min im Hellen bzw. Dunklen) ☐

Schritt 5. Auf eine Eingewöhnungszeit von ca. 2 min achten! ☐

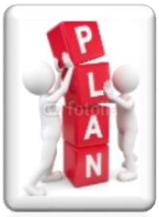

Versuchsplanung & -skizze

Betrachte die Skizze, die eure Versuchsplanung in ihren wichtigsten Schritten und Eigenschaften darstellt. **[5 min]**

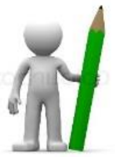

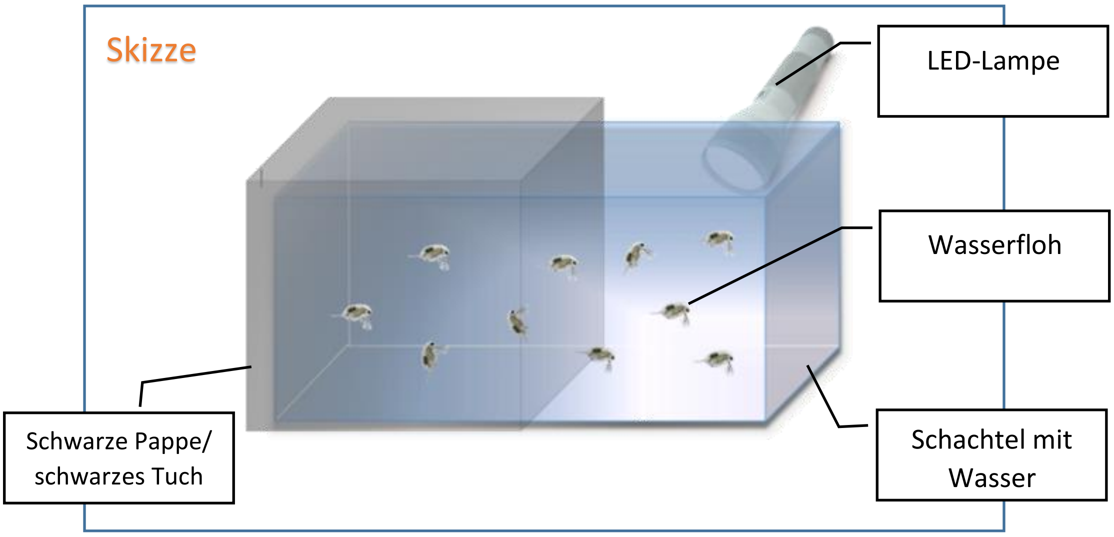

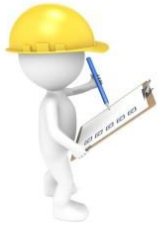

Führt euren Versuch nun durch. Achtet auf mögliche Störungen! **[20 min]**

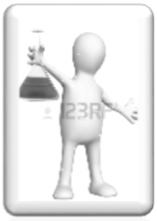

Supplement: FIGURE S10 — Research workbook_R. [file Image_10.pdf]
