# Supplementary material for: Self-Generation in the Context of Inquiry-Based Learning
Source: Front Psychol. 2018 Dec 13;9:2440. doi: 10.3389/fpsyg.2018.02440 (PMC6315139; doi:10.3389/fpsyg.2018.02440)
Supplement: FIGURE S11 — Supervisor workbook for instructions_GF. [file Image_11.pdf]

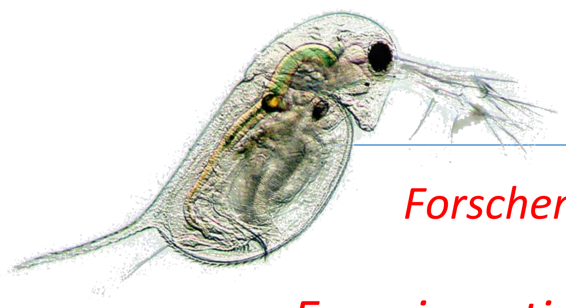

Forschen wie ein Biologe!

- Experimentieren mit Wasserflöhen

SKRIPT (Generieren)

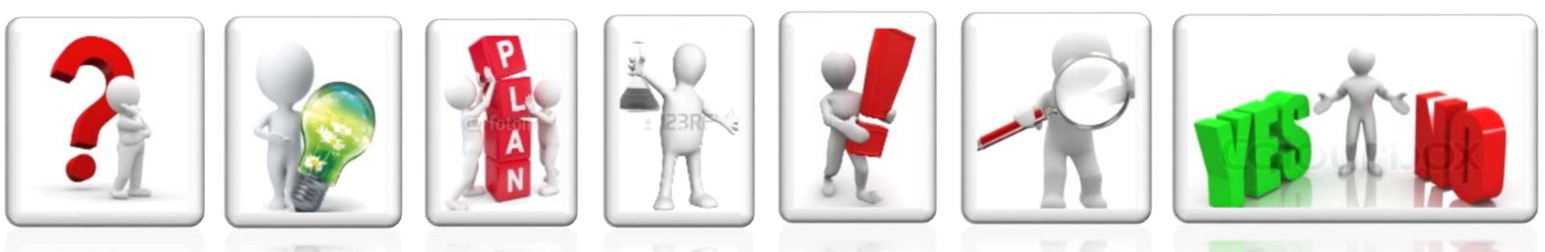

Vermutungsüberprüfung

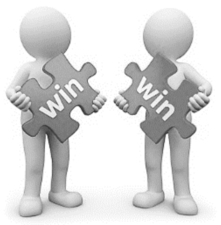

Durch den Versuch weiß ich nun, dass Ellies Vermutung

- ○ zutrifft
- ○ nicht zutrifft

Informiert euch kurz über das Phänomen: **[3 min]**

Forschungsfrage:

- ○ *Anschließend lässt er die Forscherfrage und die Beschreibung des Phänomens laut vorlesen:*

„Wie reagieren Wasserflöhe auf helle und dunkle Lichtverhältnisse?“

Die geheimnisvolle Wanderung

Jeden Tag machen die Wasserflöhe im Teich eine geheimnisvolle Wanderung. Am Abend wandern sie aus der Tiefe des Teichs nach oben, am nächsten Morgen verschwinden sie wieder nach unten. Ein Rätsel, das die Wissenschaftler lange Zeit ratlos gemacht hat. Heute ist es an euch, dieses Rätsel zu lüften!

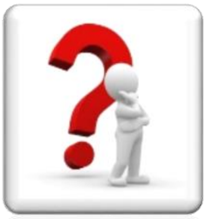

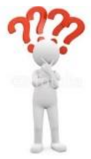

Forschungsfrage

[10 min]

Vorbereitung:

- *Der Betreuer versammelt sich mit seinem Team und lässt die Namensschildchen ausfüllen.*
- *Er lässt die Namen der SuS auf die Forscherhefte schreiben.*
- *Er erfragt, welche SuS bereits (im Unterricht) experimentiert haben.*
- *Er lässt die 7 Phasen des naturwissenschaftlichen Erkenntnisweges aufzählen und teilt anschließend die Ipads aus. Er erklärt, dass die SuS sich mit dem Ipad durch die einzelnen Phasen klicken können und zu jeder Phase eine Kurzinfo erhalten (→ Infos aus Vorinstruktion)* ***4 min*** *von 10 min hierfür verwenden*

- *Die SuS fassen das Gelernte im Anschluss an die Fehleranalyse noch einmal zusammen, indem sie den Lückentext in Einzelarbeit ausfüllen.* **[5 min]**

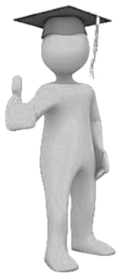

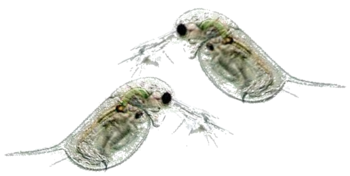

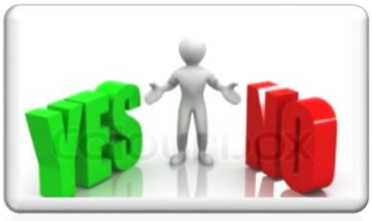

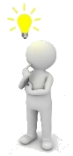

Vermutung mit Begründung

Ellie und Tom überlegen, wie Wasserflöhe auf Hell und Dunkel reagieren. **[6 min]**

Vermutung:

VORLESEN: Ellie und Tom sind zwei Schüler in eurem Alter, die sich beide überlegt haben, wie die Antwort auf eure Forscherfrage lauten könnte. Sie sind zu unterschiedlichen Vermutungen gelangt. Ein guter Forscher rät allerdings nicht einfach ins Blaue! Als Forscher muss man sein Vorwissen nutzen, um die eigene Vermutung bestmöglich zu begründen. Ellie und Tom versuchen beide ihre Vermutung so gut es geht zu begründen, um sich letztendlich auf die Vermutung mit der überzeugendsten Begründung zu einigen.

- *Die SuS lesen Ellies und Toms Vermutung zuerst im Stillen für sich und dann noch einmal laut in verteilten Rollen.*
- *Der Betreuer achtet darauf, dass alle SuS zuhören.*

VORLESEN: **Eine weitere Größe, die die Ergebnisse prägen kann, ist die Störgröße. Das sind alle Einflüsse, die neben der Testgröße auf das Verhalten der Wasserflöhe einwirken können. Es gibt verschiedene Arten von Störgrößen: Tierisch, äußere und menschliche. Prüft, welche ihr beachtet habt.**

FEEDBACK → siehe eigenes Protokoll. bei der Planung und Durchführung

Protokoll → Diskussion der SuS

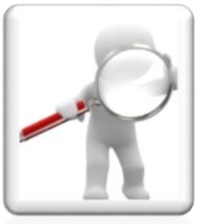

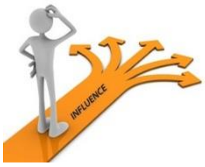

Rückblick auf das Experiment

Geht die folgende Checkliste durch und hakt all die Punkte ab, die ihr bei eurer Durchführung beachtet habt. **[7 min]**

Unsere Analyse:

- *An dieser Stelle sollen Fehler bei der Durchführung besprochen und diskutiert werden **(es soll nicht von Fehler gesprochen werden, eher von Unerwartetem, Auffälligem, Unbedachtem etc.)**.*
- *Hierzu gehen die SuS die vorgegebene Checkliste durch und haken die Punkte ab, an die sie bei der Planung/Durchführung gedacht haben.*
- *Der Betreuer achtet auf eine ehrliche Beantwortung.*
- *Den SuS diskutieren, inwieweit das Unbedachte die Aussagekraft ihrer Ergebnisse einschränkt. Der Betreuer führt Protokoll. Bitte auf Einhaltung der Zeit achten!!! (für Diskussion im Anschluss an die Checkliste: **[4min]**)*

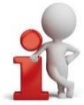

Ellie und Tom einigen sich auf eine Vermutung: **[4 min]**

Begründung:

VORLESEN: Ellie und Tom müssen sich nun einigen. Lasst uns herausfinden wie.

- *Die SuS lesen Ellies und Toms Einigung zuerst im Stillen für sich und dann erneut laut und in verteilten Rollen.*
- *Der Betreuer achtet darauf, dass alle SuS zuhören.*

Fachliche Fragen der SuS können in dieser Phase beantwortet werden(z.B. Können Wasserflöhe sehen? Was fressen Wasserflöhe? etc.)! Sie sind incl. Der Antwort, die gegeben wurde, zu notieren. *Protokoll.*

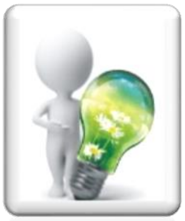

Versuchsauswertung und Ergebnisdeutung

Notiere deine Beobachtungen in der vorgegeben Tabelle. **[10 min]**

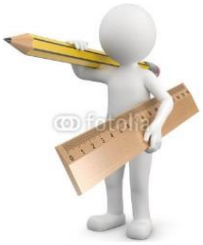

Meine Auswertung:

- *Die SuS tragen ihre erhobenen Werte in die Tabelle ein.*
- *Der Betreuer achtet darauf, dass die SuS dies eigenständig durchführen und ihre Ergebnisse an dieser Stelle noch nicht interpretieren.*
- *Anschließend werden die Ergebnisse vorgestellt.*

Präsentiere den anderen Gruppenmitgliedern deine Versuchsauswertung. **[5 min]**

- *Die SuS legen die 5 Schritte zur Versuchsplanung fest. FEEDBACK*

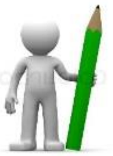

Überarbeitet eure Versuchsplanung und fertigt nun gemeinsame eine Skizze an, die alle wichtigen Eigenschaften eurer Versuchsplanung abbildet. **[5 min]**

Führt euren Versuch nun wie geplant durch. Achtet auf mögliche Störquellen! **[20 min]**

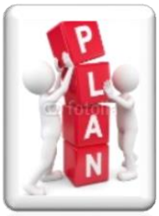

Versuchsplanung & -skizze

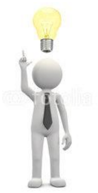

Ellie und Tom haben die Test- und Messgröße sowie den Kontrollansatz für ihren Versuch folgendermaßen bestimmt: **[4 min]**

VORLESEN: Ihr seid nun ein Forscherteam und werdet gemeinsam ein Experiment durchführen.
Mit dem Experiment könnt ihr Ellies und Toms gemeinsame Vermutung überprüfen.
Bevor ihr mit der Planung startet, müsst ich euch drei Dinge genau überlegen:

Der Betreuer lässt sie SuS die Test-, Messgröße und den Kontrollansatz bestimmen. *FEEDBACK*

*Anschließend dürfen sich die SuS einen Überblick über das Material verschaffen (***[3 min]***).*

Findet heraus, zu wessen Ergebnissen eure Ergebnisse passen und setzt ein Kreuz. **[10 min]**

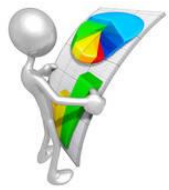

Ellies und Toms Deutung:

Die SuS lesen beide Deutungen zuerst im Stillen für sich und dann noch einmal laut und in verteilten Rollen. Sie entscheiden dann welche Deutung zu ihren Ergebnissen passt (entweder Ellies oder Toms).Dort setzen sie ein Kreuz.

Der Betreuer zieht sich weitestgehend zurück.

Fachliche Fragen (z.B. zum Aufbau der Wasserflöhe, Körperteile etc.) der SuS dürfen in dieser Phase beantwortet werden! Fragen und Antworten bitte wieder notieren. *Protokoll.*

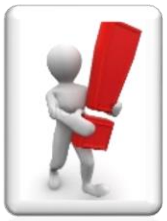

Durchführung

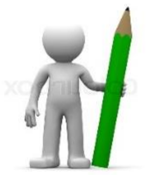

Unsere Skizze

- *Die SuS versuchen ihren Plan in einer Skizze zu veranschaulichen.*
- *Der Betreuer achtet darauf, dass jede/r SuS eine saubere Skizze anfertigt und diese im Detail beschriften.*

Führt euren Versuch nun wie geplant durch. Achtet auf mögliche Störungen! **[20 min]**

- *Der Versuch wird in* ***zwei Teilgruppen*** *parallel durchgeführt*
- *Der Betreuer unterstützt die SuS bei der Durchführung des Versuchs und gibt kleine Hilfestellungen. KEIN METHODISCHER INPUT!!!*
- *Fehler und Ungenauigkeiten sollen von dem Betreuer beobachtet und notiert werden, um diese mit den SuS bei der Fehleranalyse zu diskutieren.* *Protokoll.*
- *Der Betreuer koordiniert das Aufräumen im Anschluss an das Experiment.*

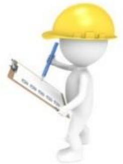

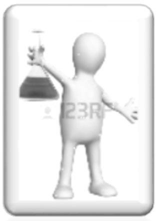

FEEDBACK

Erste Überlegungen

Testgröße: Es soll der Einfluss von Licht auf das Verhalten der Wasserflöhe getestet werden.

Messgröße: Der Einfluss auf das Verhalten kann daran beobachtet/ gemessen werden, dass die Wasserflöhe Folgendes tun: Sie wandern vom Licht weg.

Kontrollansatz: Dass das Verhalten tatsächlich durch die Testgröße ausgelöst wurde, wird folgendermaßen kontrolliert: Die Wasserflöhe können zwischen einem hellen und einem gleichgroßen dunklen Bereich wählen.

Was wurde falsch ausgefüllt? Wie oft? Wie?

	Testgröße	Messgröße	Kontrollansatz
Was?			
Wie oft?			
Wie?			

Plan

Schritt 1. Folgende Materialien beschaffen: **schachtelförmiger Behälter, LED-Lampe, Pipette, schwarzer Karton, Stoppuhr, 10 Wasserflöhe, Wasser**.

Schritt 2. Behälter zu ¾ mit Wasser füllen und **10 Wasserflöhe** hineinsetzen. →WARUM mehrere?

Schritt 3. **Genau die Hälfte** des Behälters mit **schwarzem Karton** abdecken (*Testgröße: Licht, Kontrollansatz: $\frac{1}{2}$ dunkler Bereich)*.
Andere Hälfte des Behälters **10 min** seitlich mit der **Taschenlampe** (Abstand: ca. eine Fingerlänge) bestrahlen.

Schritt 4. Notieren, **wie viele Wasserflöhe sich pro Minute** im abgedunkelten bzw. hellen Bereich befinden. (*Messgröße: Anzahl der Wasserflöhe pro min im Hellen bzw. Dunklen*)

Schritt 5. Auf eine **Eingewöhnungszeit** von ca. 2 min achten!

Was wurde falsch ausgefüllt? Wie oft? Wie?

	Schritt 1	Schritt 2	Schritt 3	Schritt 4	Schritt 5
Was?					
Wie oft?					
Wie?					

PROTOKOLL: RÜCKBLICK AUF DAS EXPERIMENT/ FEHLERANALYSE

Beobachtete Fehler bei Planung/Durchführung:

- ..
- ..
- ..
- ..
- ..

- ..
- ..
- ..

Rückblick auf das Experiment

FEEDBACK

HUNGER:

Wenn nur einige der Tiere gefüttert wurden, können sie sich unterschiedlich verhalten. Hungrige Wasserflöhe schwimmen ins Licht, die anderen ins Dunkle.

GLEICHGROẞE Bereiche:

Man hält diese gleich, damit man eindeutig erkennen kann, ob tatsächlich das Licht das Verhalten der Wasserflöhe auslöst und nicht etwa die Größe des hellen Bereichs.
Es könnte nämlich auch sein, dass sie ins Dunkle schwimmen, weil sie sich eingeengt fühlen, wenn man ihnen einen kleinen hellen Bereich zur Verfügung stellt. (*oder andersherum*)

MEHRERE Versuchstiere:

Je mehr Versuchstiere man nimmt, desto wahrscheinlicher ist es, dass das Ergebnis des Experiments nicht durch puren Zufall zustande kommt.

LED:

Würde man eine Taschenlampe verwenden, wüsste man letztendlich nicht, ob das Licht oder die Wärme der Taschenlampe das Verhalten der Libellenlarve beeinflusst hat!

Diskussion im Anschluss an das Experiment:

Allgemeine Übersicht

Schule:________________________________ Klasse: _______ Datum: _________

**Zuordnung**		Schüler
	1	
	2	
	3	
	4	
	5	
	6	
**Besondere Vorkommnisse**	Allgemeine Probleme:	
	Besondere Fragen der Schüler:	
	Störungen durch Schüler:	
	Tipps:	

Skript Experimentiermodul Wasserfloh Licht (Generieren)

	Zeit (Min)	Inhalt	Anweisung
	5	Begrüßung und Übersicht über die Einheit, Verhaltensregeln ansprechen, falls nötig	
Predict	10-12	Film und Fragestellung, Gruppenaufteilung	
	10	Einführung in experimentelles Arbeiten und Wdh. der Fragestellung	In allen Gruppen einheitlich!
	8	Hypothesenfindung	In allen Gruppen einheitlich!
Observe	12 – (15)	Planung des Experimentes	Unterschiedlich in den Gruppen!<br>Methodische Fragen (z.B. Wie viele Wasserflöhe? Wie viele Minuten? Welches Material? Etc.) der SchülerInnen sollen in dieser Phase nicht beantwortet werden!<br>Fehlerhafte Planung soll zunächst nicht korrigiert werden!!<br>ACHTUNG: Im Anschluss an Planung → Feedback!!
	Pause 15 min		
	20	Durchführung des Experiments	In allen Gruppen einheitlich!
	5	Aufräumen	
Explain	8	Auswertung der Ergebnisse	In allen Gruppen einheitlich!
	10	Interpretation der Ergebnisse und Rückbindung zum Phänomen	In allen Gruppen einheitlich!
	7- (10)		Unterschiedlich in den Gruppen!<br>Methodische Fragen (z.B. Wie viele Wasserflöhe? Wie viele Minuten? Welches Material? Etc.) der SchülerInnen sollen in dieser Phase nicht beantwortet werden!<br>Fehlerhafte Planung soll zunächst nicht korrigiert werden!!<br>ACHTUNG: Im Anschluss an Checkliste → Feedback durch Diskussion!!
	5	Zusammenfassen der Ergebnisse	Unterschiedlich in den Gruppen!<br>*Die wichtigsten Ergebnisse werden in Einzelarbeit in Rahmen eines Lückentextes festgehalten.*
			ZEITLICHER PUFFER in Lesegruppe<br>• *Binos/ Mikroskope (inklusive Pipetten, Schälchen, Becherglas) sind bereits vorbereitet*<br>• *Die Hilfskraft hat die Aufgabe, die SchülerInnen beim Mikroskopieren zu unterstützen.*
	ca. 2 Std		
