# Supplementary material for: Self-Generation in the Context of Inquiry-Based Learning
Source: Front Psychol. 2018 Dec 13;9:2440. doi: 10.3389/fpsyg.2018.02440 (PMC6315139; doi:10.3389/fpsyg.2018.02440)
Supplement: FIGURE S12 — Supervisor workbook for instructions_R. [file Image_12.pdf]

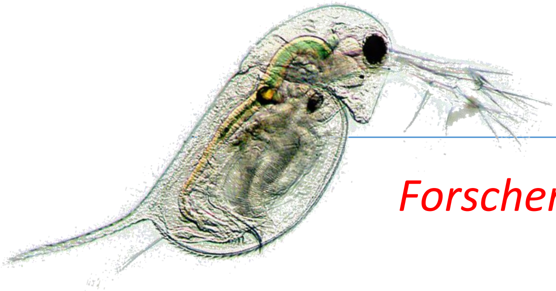

Forschen wie ein Biologe!

- Experimentieren mit Wasserflöhen

SKRIPT (LESEN)

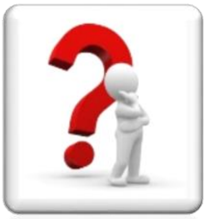

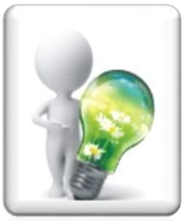

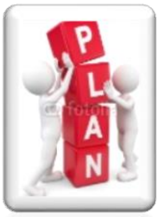

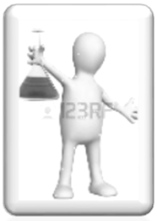

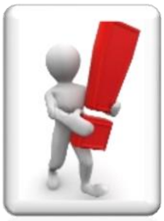

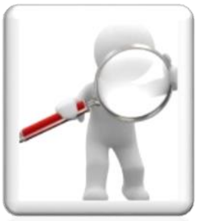

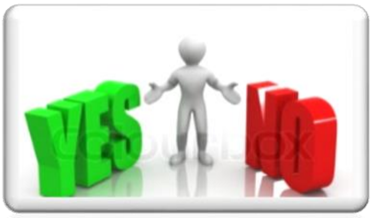

Vermutungsüberprüfung

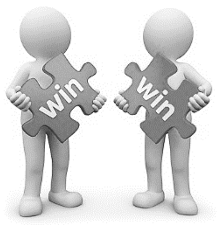

Durch den Versuch weiß ich nun, dass Ellies Vermutung

- ○ zutrifft
- ○ nicht zutrifft

Informiert euch kurz über das Phänomen: **[3 min]**

Forschungsfrage:

- ○ *Anschließend lässt er die Forscherfrage und die Beschreibung des Phänomens laut vorlesen:*

„Wie reagieren Wasserflöhe auf helle und dunkle Lichtverhältnisse?“

Die geheimnisvolle Wanderung

Jeden Tag machen die Wasserflöhe im Teich eine geheimnisvolle Wanderung. Am Abend wandern sie aus der Tiefe des Teichs nach oben, am nächsten Morgen verschwinden sie wieder nach unten. Ein Rätsel, das die Wissenschaftler lange Zeit ratlos gemacht hat. Heute ist es an euch, dieses Rätsel zu lüften!

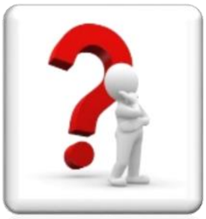

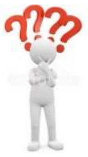

Forschungsfrage

[10 min]

Vorbereitung:

- *Der Betreuer versammelt sich mit seinem Team und lässt die Namensschildchen ausfüllen.*
- *Er lässt die Namen der SuS auf die Forscherhefte schreiben.*
- *Er erfragt, welche SuS bereits (im Unterricht) experimentiert haben.*
- *Er lässt die 7 Phasen des naturwissenschaftlichen Erkenntnisweges aufzählen und teilt anschließend die Ipads aus. Er erklärt, dass die SuS sich mit dem Ipad durch die einzelnen Phasen klicken können und zu jeder Phase eine Kurzinfo erhalten (→ Infos aus Vorinstruktion)* ***4 min*** *von 10 min hierfür verwenden*

- *Die SuS fassen das Gelernte im Anschluss an die Fehleranalyse noch einmal zusammen.*
  - *jeder liest im Stillen für sich!*

[5 min]

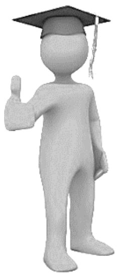

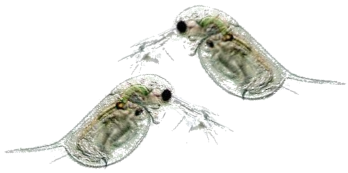

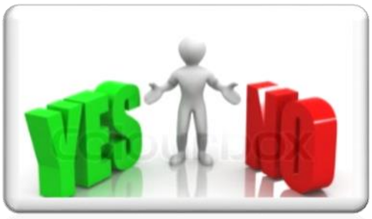

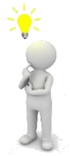

Vermutung mit Begründung

Ellie und Tom überlegen, wie Wasserflöhe auf Hell und Dunkel reagieren. **[6 min]**

Vermutung:

VORLESEN: Ellie und Tom sind zwei Schüler in eurem Alter, die sich beide überlegt haben, wie die Antwort auf eure Forscherfrage lauten könnte. Sie sind zu unterschiedlichen Vermutungen gelangt. Ein guter Forscher rät allerdings nicht einfach ins Blaue! Als Forscher muss man sein Vorwissen nutzen, um die eigene Vermutung bestmöglich zu begründen. Ellie und Tom versuchen beide ihre Vermutung so gut es geht zu begründen, um sich letztendlich auf die Vermutung mit der überzeugendsten Begründung zu einigen.

- *Die SuS lesen Ellies und Toms Vermutung zuerst im Stillen für sich und dann noch einmal laut in verteilten Rollen.*
- *Der Betreuer achtet darauf, dass alle SuS zuhören.*

Typische Fehler bei der Durchführung:

Tierischer Einfluss:

…. z.B. Eingewöhnungszeit der Wasserflöhe(ca. 2 min),
Eigenheiten eines Lebewesens beachtet (Hunger, Gemütszustand usw.)

Äußerer Einfluss:

…. z.B. äußeren Lichteinfall (Fenster, Raumlicht), möglichst geringe(bis keine) Lichtspiegelung in der Schachtel, Ruhe im Klassenraum

Menschlicher Einfluss:

…. z.B. Gleichhaltung des dunklen und hellen Wasserbereich, Verwendung ausreichend vieler Wasserflöhe (ca. 10 oder mehr), keine Veränderungen nach Durchführungsbeginn (wie Stöße gegen den Tisch, Änderung der Position der Taschenlampe)

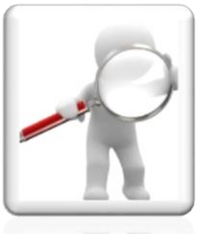

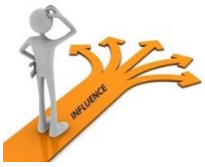

Rückblick auf das Experiment

Man kann sich auf die Ergebnisse eines Experiments nicht immer verlassen. Denn manchmal gibt es kleine Unaufmerksamkeiten, die eure Ergebnisse schnell verfälschen können: **[7 min]**

Unsere Analyse:

- *An dieser Stelle sollen Fehler bei der Durchführung besprochen werden.* ***(Es soll nicht von Fehler gesprochen werden, eher von Unerwartetem, Auffälligem, Unbedachtem etc.)****.*
- *Hierzu lesen die SuS die kurzen Abschnitte zu möglichen Einflüssen.*

VORLESEN: …

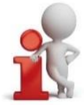

Ellie und Tom einigen sich auf eine Vermutung: **[4 min]**

Begründung:

VORLESEN: Ellie und Tom müssen sich nun einigen. Lasst uns herausfinden wie.

- *Die SuS lesen Ellies und Toms Einigung zuerst im Stillen für sich und dann erneut laut und in verteilten Rollen.*
- *Der Betreuer achtet darauf, dass alle SuS zuhören.*

Fachliche Fragen der SuS können in dieser Phase beantwortet werden(z.B. Können Wasserflöhe sehen? Was fressen Wasserflöhe? etc.)! Sie sind incl. Der Antwort, die gegeben wurde, zu notieren. *Protokoll.*

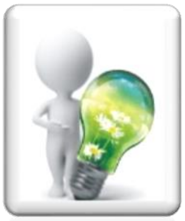

Versuchsauswertung und Ergebnisdeutung

Notiere deine Beobachtungen in der vorgegeben Tabelle. **[10 min]**

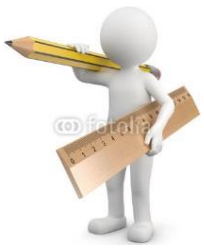

Meine Auswertung:

- *Die SuS tragen ihre erhobenen Werte in die Tabelle ein.*
- *Der Betreuer achtet darauf, dass die SuS dies eigenständig durchführen und ihre Ergebnisse an dieser Stelle noch nicht interpretieren.*
- *Anschließend werden die Ergebnisse vorgestellt.*

Präsentiere den anderen Gruppenmitgliedern deine Versuchsauswertung. **[5 min]**

Verschafft euch einen Überblick über das Material, das euch für euren Versuch zur Verfügung steht. **[10 min]**

Lest euch die Versuchsplanung aufmerksam durch. **[4 min]**

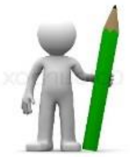

- Der Betreuer lässt die SuS den Versuchsplan in Ruhe lesen und erkundigt sich, ob es Nachfragen gibt. Diese werden inkl. der Antwort notiert.
- Der Betreuer achtet darauf, dass die SuS die einzelnen Schritte im Detail verstanden haben.

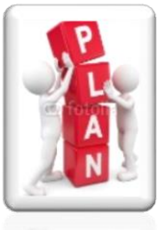

Versuchsplanung & -skizze

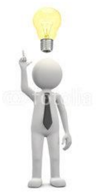

Ellie und Tom haben die Test- und Messgröße sowie den Kontrollansatz für ihren Versuch folgendermaßen bestimmt: **[4 min]**

VORLESEN: Ihr seid nun ein Forscherteam und werdet gemeinsam ein Experiment durchführen. Mit dem Experiment könnt ihr Ellies und Toms gemeinsame Vermutung überprüfen. Bevor Ellie und Tom mit der Planung starten, stellen sie erste wichtige Überlegungen an:

Der Betreuer lässt sie SuS Ellies und Toms erste Überlegungen lesen.

*Anschließend dürfen sich die SuS einen Überblick über das Material verschaffen (***[3 min]***).*

→ *SuS mikroskopieren Wasserflöhe und zählen Herzschlag pro Minute. (***[5-10 min]***)*

Der Betreuer lässt die SuS den Versuchsplan in Ruhe lesen und erkundigt sich, ob es Nachfragen gibt. Diese werden inkl. der Antwort notiert.

Findet heraus, zu wessen Ergebnissen eure Ergebnisse passen und setzt ein Kreuz. **[10 min]**

Ellies und Toms Deutung:

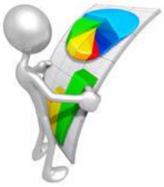

Die SuS lesen beide Deutungen zuerst im Stillen für sich Sie entscheiden dann welche Deutung zu ihren Ergebnissen passt (entweder Ellies oder Toms).Dort setzen sie ein Kreuz und lesen die passende Deutung dann noch einmal laut und in verteilten Rollen.

Der Betreuer zieht sich weitestgehend zurück.

Fachliche Fragen (z.B. zum Aufbau der Wasserflöhe, Körperteile etc.) der SuS dürfen in dieser Phase beantwortet werden! Fragen und Antworten bitte wieder notieren. *Protokoll.*

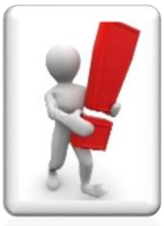

Durchführung

Betrachte die Skizze, die eure Versuchsplanung in ihren wichtigsten Schritten und Eigenschaften darstellt. **[5 min]**

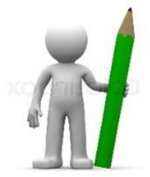

Unsere Skizze

- *Die SuS betrachten die Skizze bevor sie mit dem Experiment loslegen.*

Führt euren Versuch nun wie geplant durch. Achtet auf mögliche Störungen! **[20 min]**

- *Der Versuch wird in* ***zwei Teilgruppen*** *parallel durchgeführt*
- *Der Betreuer unterstützt die SuS bei der Durchführung des Versuchs und gibt kleine Hilfestellungen. KEIN METHODISCHER INPUT!!!*
- *Fehler und Ungenauigkeiten sollen von dem Betreuer beobachtet und notiert werden, um diese mit den SuS bei der Fehleranalyse zu diskutieren.* *Protokoll.*
- *Der Betreuer koordiniert das Aufräumen im Anschluss an das Experiment.*

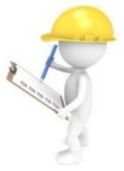

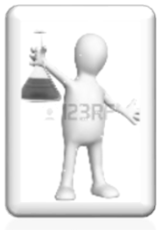

Allgemeine Übersicht

Schule:________________________________ Klasse: ________ Datum: __________

**Zuordnung**		Schüler
	1	
	2	
	3	
	4	
	5	
	6	
**Besondere Vorkommnisse**	Allgemeine Probleme:	
	Besondere Fragen der Schüler:	
	Störungen durch Schüler:	
	Tipps:	

Protokoll:

PLANUNG

Durchführung

Rückblick auf das Experiment

HUNGER:

Wenn nur einige der Tiere gefüttert wurden, können sie sich unterschiedlich verhalten. Hungrige Wasserflöhe schwimmen ins Licht, die anderen ins Dunkle.

GLEICHGROßE Bereiche:

Man hält diese gleich, damit man eindeutig erkennen kann, ob tatsächlich das Licht das Verhalten der Wasserflöhe auslöst und nicht etwa die Größe des hellen Bereichs.
Es könnte nämlich auch sein, dass sie ins Dunkle schwimmen, weil sie sich eingeengt fühlen, wenn man ihnen einen kleinen hellen Bereich zur Verfügung stellt. (*oder andersherum*)

MEHRERE Versuchstiere:

Je mehr Versuchstiere man nimmt, desto wahrscheinlicher ist es, dass das Ergebnis des Experiments nicht durch puren Zufall zustande kommt.

LED:

Würde man eine Taschenlampe verwenden, wüsste man letztendlich nicht, ob das Licht oder die Wärme der Taschenlampe das Verhalten der Libellenlarve beeinflusst hat!

UNI KASSEL VERSITÄT

FLOX

Skript Experimentiermodul Wasserfloh Licht (Lesen)

	Zeit (Min)	Inhalt	Anweisung
	5	Begrüßung und Übersicht über die Einheit, Verhaltensregeln ansprechen, falls nötig	
Predict	10-12	Film und Fragestellung, Gruppenaufteilung	
	10	Einführung in experimentelles Arbeiten und Wdh. der Fragestellung	In allen Gruppen einheitlich!
	8	Hypothesenfindung	In allen Gruppen einheitlich!
Observe	12 – (15)	Planung des Experimentes	Unterschiedlich in den Gruppen!<br>Methodische Fragen (z.B. Wie viele Wasserflöhe? Wie viele Minuten? Welches Material? Etc.) der SchülerInnen sollen in dieser Phase nicht beantwortet werden!<br>Fehlerhafte Planung soll zunächst nicht korrigiert werden!!
	Pause 15 min		
	20	Durchführung des Experiments	In allen Gruppen einheitlich!
	5	Aufräumen	
Explain	8	Auswertung der Ergebnisse	In allen Gruppen einheitlich!
	10	Interpretation der Ergebnisse und Rückbindung zum Phänomen	In allen Gruppen einheitlich!
	7- (10)	Rückblick auf das Experiment	Unterschiedlich in den Gruppen!<br>Methodische Fragen (z.B. Wie viele Wasserflöhe? Wie viele Minuten? Welches Material? Etc.) der SchülerInnen sollen in dieser Phase nicht beantwortet werden!<br>Fehlerhafte Planung soll zunächst nicht korrigiert werden!!
	5	Zusammenfassen der Ergebnisse	Unterschiedlich in den Gruppen!<br>*Die wichtigsten Ergebnisse werden in Einzelarbeit in Rahmen eines Lückentextes festgehalten.*
			ZEITLICHER PUFFER<br>• *Binos/ Mikroskope (inklusive Pipetten, Schälchen, Becherglas) sind bereits vorbereitet*<br>• *Die Hilfskraft hat die Aufgabe, die SchülerInnen beim Mikroskopieren zu unterstützen.*
	ca. 2 Std		

UNI KASSEL
VERSITÄT

FLOX
