# Supplementary material for: Self-Generation in the Context of Inquiry-Based Learning
Source: Front Psychol. 2018 Dec 13;9:2440. doi: 10.3389/fpsyg.2018.02440 (PMC6315139; doi:10.3389/fpsyg.2018.02440)
Supplement: TABLE S1 — Example_anchor item. [file Table_1.pdf]

Pretest:

Plant growth

Amelie wants to find out which soil is best suited to support the growth of beans. She assumes that beans grow better in garden soil than in clay soil. To verify her suspicion, she takes two pots and fills them with two kinds of soil. She plants several beans in both pots and places them on the windowsill above the heating in her room. Amelie knows that plants need water and minerals to grow. That´s why she waters and fertilizes the beans weekly.

Experimental approaches used by Amelie

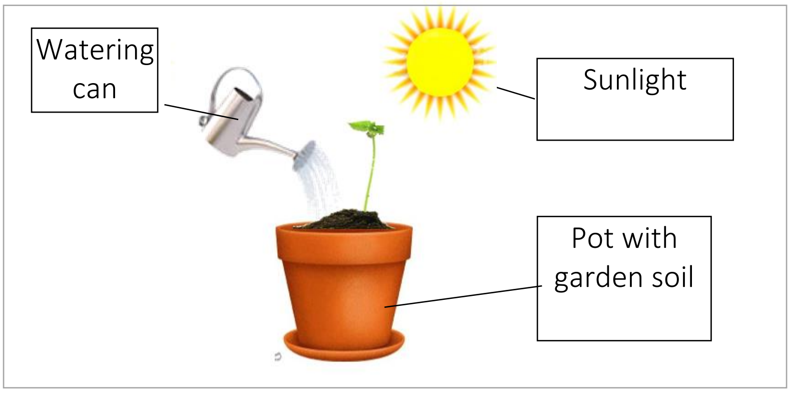

Task

Which second experimental approach does Amelie need? Mark with a cross! *(P_Pfl_B_MC)*

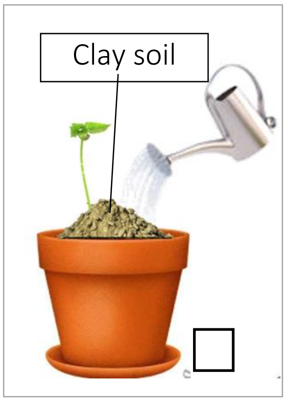

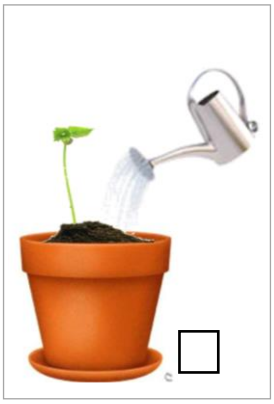

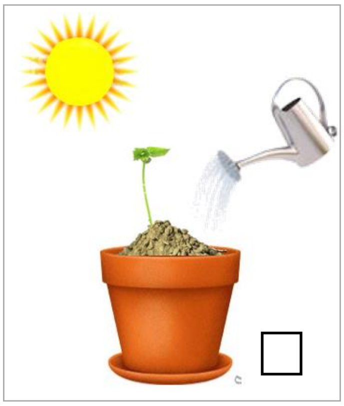

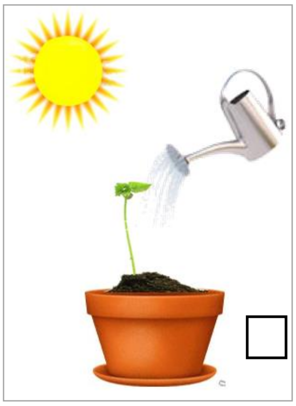

Posttest 1

Plant growth

Amelie wants to find out which lighting conditions are best suited to support the growth of beans. She suspects that beans grow better in sunlight than in shade. To verify her suspicion, she plants several beans in two pots with garden soil. She puts a pot on the windowsill in the sunlight and a pot in a shady place on her bookshelf. Amelie knows that plants need water and minerals to grow. That´s why she waters and fertilizes the beans weekly.

Experimental approaches used by Amelie

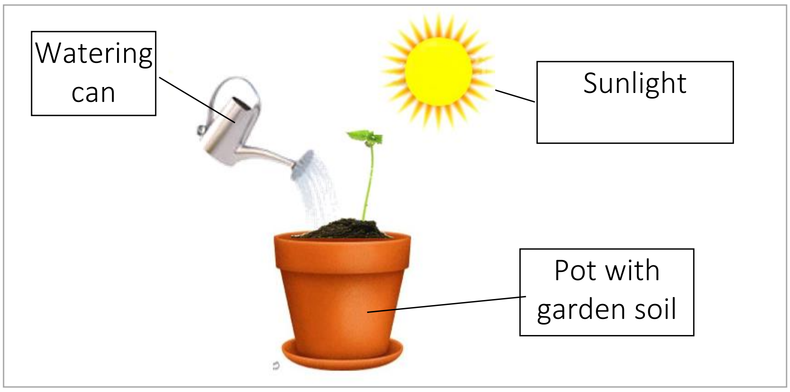

Task

Which second experimental approach does Amelie need? Mark with a cross! *(E1_Pfl_L_MC)*

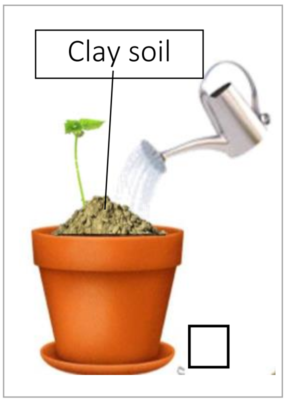

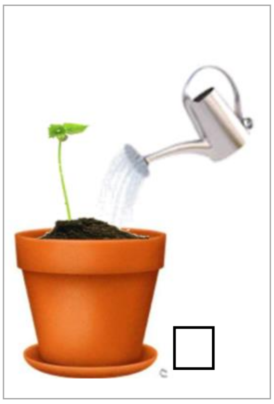

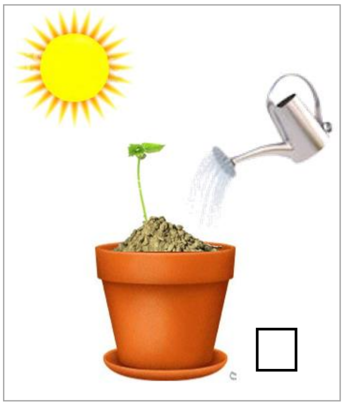

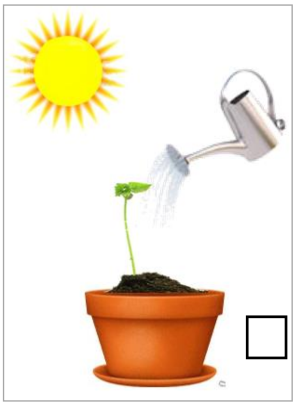

Posttest 2:

Plant growth

Amelie wants to find out how much water is best suited to support the growth of beans. She suspects that beans grow better if they are watered frequently. To verify her suspicions, she takes two pots and plants several beans in both pots. She puts them on the windowsill above heating in her room. Amelie knows that plants needs minerals for their growth. That´s why she fertilizes the beans weekly.

Experimental approaches used by Amelie

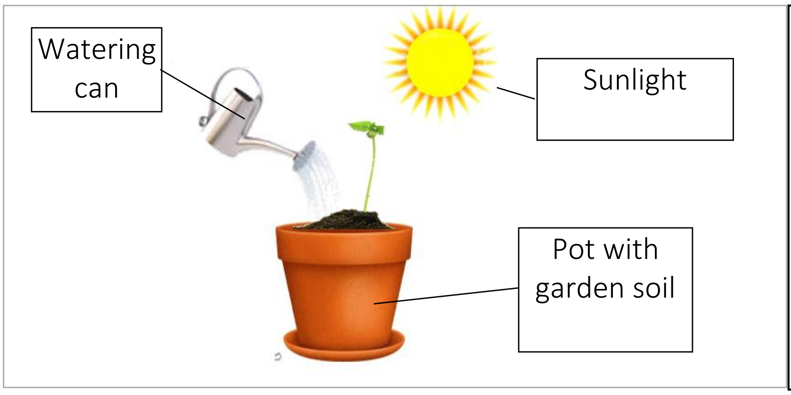

To water: daily
To fertilize: once a week

Task

Which second experimental approach does Amelie need? Mark with a cross! *(E2_Pfl_W_MC)*

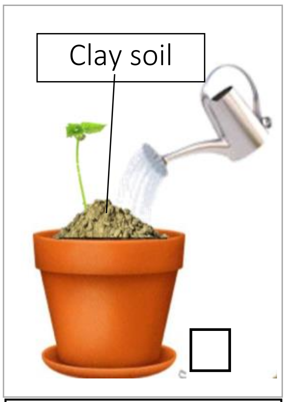

To water: daily
To fertilize: once a week

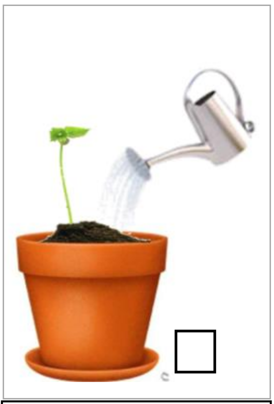

To water: daily
To fertilize: once a week

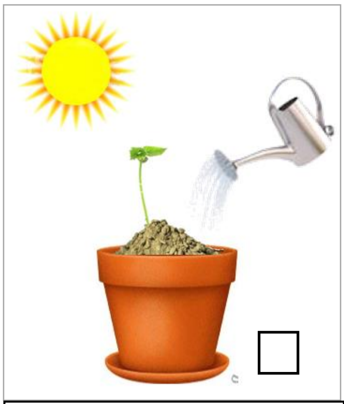

To water and fertilize: once a week

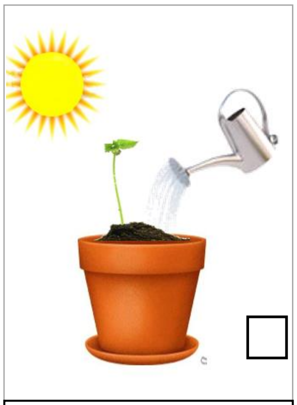

To water and fertilize: once a week

Follow up:

Plant growth

Amelie wants to find out if fertilizer accelerates the growth of beans. She suspects that beans grow better when they are fertilized regularly. To verify her presumption, she takes two pots and plants several beans in both pots. She puts them on the windowsill above the heating in her room. Amelie knows that plants need water to grow. That's why she waters the beans regularly.

Experimental approaches used by Amelie

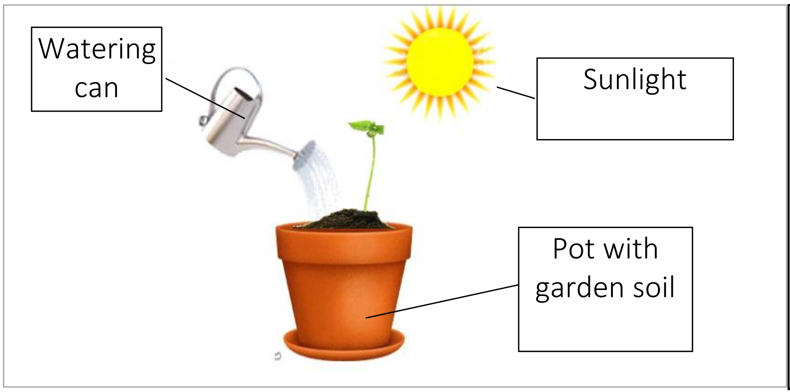

To water: daily
To fertilize: once a week

Task

Which second experimental approach does Amelie need? Mark with a cross! *(E3_Pfl_D_MC)*

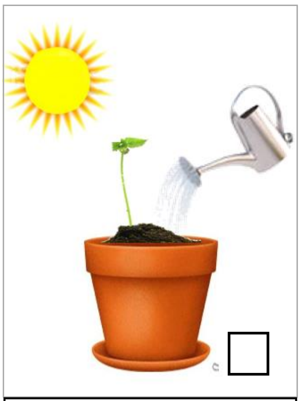

To water: daily
To fertilize: never

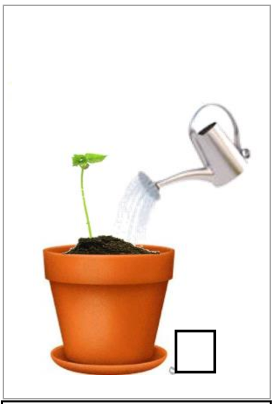

To water: daily
To fertilize: once a week

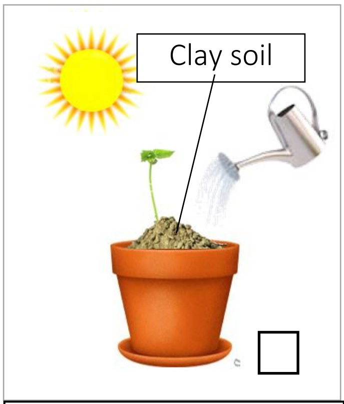

To water: daily
To fertilize: once a week

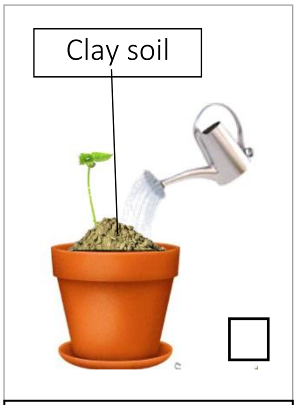

To water: daily
To fertilize: never
