# Supplementary material for: Self-Generation in the Context of Inquiry-Based Learning
Source: Front Psychol. 2018 Dec 13;9:2440. doi: 10.3389/fpsyg.2018.02440 (PMC6315139; doi:10.3389/fpsyg.2018.02440)
Supplement: TABLE S2 — Example 1, Example 2, Example 3, and Example 4. [file Table_2.pdf]

Example 1. Self-generation prompt.

Experiment Plan and Outline

Try to come up with an experiment that you can use to test Ellie's hypothesis.
As part of this, mark down your initial thoughts. **[4 min]**

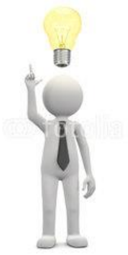

My initial thoughts

What will be tested: The influence of __________ on water fleas' behavior will be tested.

What will be measured: The influence on behavior can be observed/measured when the water fleas do the following: ____________________________________

What controls will be made: We will control in the following way for whether the behavior was actually triggered by the test variable:

____

Example 2. Self-generation prompts in a cloze section.

[10 min]

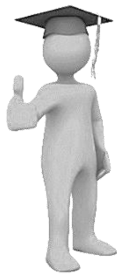

I learned the following things about experimentation:

In our experiment, we investigated the influence of ___________________on water fleas' behavior (test dimension). We ___________________ for whether their behavior was actually triggered by the light by providing the water fleas with a ___________________ and a ________________________area.
The size of the two areas was ___________________.

…

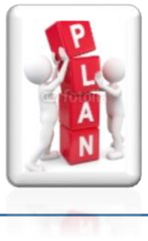

Example 3. Feedback given by the supervisor.

Initial thoughts

Test dimension: The influence of **light** on water fleas' behavior will be tested.

Measurement dimension: The influence on behavior can be observed/measured when the water fleas do the following: **They migrate away from the light.**

Control approach: We will control in the following way for whether the behavior was actually triggered by the test variable: **The water fleas can choose between a light area and a dark area of the same size.**

What was filled in incorrectly? How often? How?

	Test dimension	Measurement dimension	Control approach
What?			
How often?			
How?			

Example 4. Feedback.

Mistakes observed in planning/conducting the experiment:

- ..
- ..
- ..
- ..
- ..

Looking back on the experiment: Discussion of individual confounding factors

HUNGER:

If only some of the animals are fed, they can behave differently. Hungry water fleas swim towards light, while the others swim towards darkness.

Areas of the SAME SIZE:

These should be held constant in order to unequivocally determine whether it is actually light that is triggering the water fleas' behavior and not the size of the light area. They might also swim towards darkness because they feel constricted when provided with a small light area (*or vice versa*).

MULTIPLE test animals:

The more test animals considered, the more likely it is that the results of the experiment did not arise purely by chance.

LED:

If a flashlight is used, it is impossible to know in the end whether the light or the warmth of the flashlight influenced the water fleas' behavior!

Discussion following the experiment:

- ..
- ..
- ..
- ..
- ..
- ..
- ..
